# Recent developments in enantioselective photocatalysis

**DOI:** 10.3762/bjoc.16.197

**Published:** 2020-09-29

**Authors:** Callum Prentice, James Morrisson, Andrew D Smith, Eli Zysman-Colman

**Affiliations:** 1 Organic Semiconductor Centre, EaStCHEM, School of Chemistry, University of St Andrews, North Haugh, Fife, Scotland, KY16 9ST, United Kingdom; 2 Pharmaceutical Sciences, IMED Biotech Unit, AstraZeneca, Macclesfield SK102NA, United Kingdom

**Keywords:** enantioenrichment, enantionselective catalysis, enantioselective photocatalysis, photocatalysis, photochemistry

## Abstract

Enantioselective photocatalysis has rapidly grown into a powerful tool for synthetic chemists. This review describes the various strategies for creating enantioenriched products through merging enantioselective catalysis and photocatalysis, with a focus on the most recent developments and a particular interest in the proposed mechanisms for each. With the aim of understanding the scope of each strategy, to help guide and inspire further innovation in this field.

## Introduction

Enantioselective catalysis has become a central focus for organic synthetic chemistry, particularly since the Nobel prize was awarded to Sharpless, Knowles, and Noyori for their pioneering work in the field. In the last 15 years, photocatalysis has become a transformative synthetic strategy, including in enantioselective synthesis. From the pioneering work by MacMillan [1] and Bach [2], enantioselective photocatalysis has grown into a well-established field of its own. A large proportion of photocatalysis focuses on photoredox catalysis, which involves single electron transfer (SET) steps photoinitiated using visible light as the energy source, often leading to the generation of radicals and subsequent reaction of these radicals with the ground-state substrates [3]. Energy transfer catalysis is another significant branch of photocatalysis, in which photocatalysts (PCs) generate excited state substrates that can then undergo reactions that would be impossible in the ground state [4]. A challenge for enantioselective catalysis is stifling the racemic background reaction, which is generally achieved through a lower activation energy for the catalysed process relative to the non-catalysed. However, this is particularly difficult for enantioselective photocatalysis as the intermediates generated are highly reactive and activation energies are typically already low [5]. Even so, there are now a large number of reactions that have been developed that address these issues, with many different strategies and types of photocatalysts employed.

This review aims to cover the seminal work within enantioselective photocatalysis but with a focus on the most recent developments. There have been a number of reviews on or closely related to this topic, so this review will not contain an exhaustive list of all enantioselective photocatalytic reactions; however, this review does aim to cover the different strategies that have been developed [5–16]. There is a subset of reactions that achieve asymmetry via a stepwise photochemical process followed by a separate enantioselective catalysis step that will not be covered in this review [17–19]. Examples using cage complexes or other supramolecular reagents also lie outside the scope of this review. As all enantioselective photocatalysis requires a secondary mode of catalysis to induce enantioselectivity, the review will be organized according to these strategies. Mechanistic understanding is vital to furthering development of any field of organic chemistry, so the mechanisms proposed by the authors are included for many examples, although the level of mechanistic investigation that accompanies them is varied. The nature of the light source and its wavelength (λ_exc_) can have significant effects on the outcome of the reaction; therefore, this information is indicated in the reaction schemes when it is disclosed by the authors. As with many reviews on enantioselective reactions, the percentage chemical yield and enantioselectivity (all values converted to nearest per cent enantiomeric ratio (er) for clarity) will be the common data for comparison; however, where possible the quantum yields of the photochemical reactions will also be provided as this latter metric provides the most accurate quantifier of the efficiency of a photochemical process. The quantum yield (Φ) of a reaction is often used as a mechanistic tool to probe whether a chain reaction is active (Φ > 1) or not (Φ < 1), although it should be noted that a reaction with Φ < 1 could also still include a radical chain process with an inefficient initiation step (i.e., Φ_initiation_ << 1) (Equation 1). A less common use of quantum yields by organic synthetic chemists is as a measure of how efficiently the reaction uses the light source. Considering photocatalysis is often purported as a green chemistry because it uses light, a more efficient use of light would result in a greener reaction.

[1] $$\begin{array}{l} \text{a)}\;\;\;\;\;\;\Phi =\frac{\text{product}\;\text{formed}}{\text{photons}\;\text{absorbed}}\\ \text{b)}\;\;\;\;\;\;\Phi =\Phi_{\text{initiation}}\times \text{chain}\;\text{lenght} \end{array}$$

where (a) is the definition of quantum yield and (b) is the quantum yield of a radical chain reaction.

## Review

### Enantioselective photocatalysis

#### Amine catalysis

**Scheme 1:**
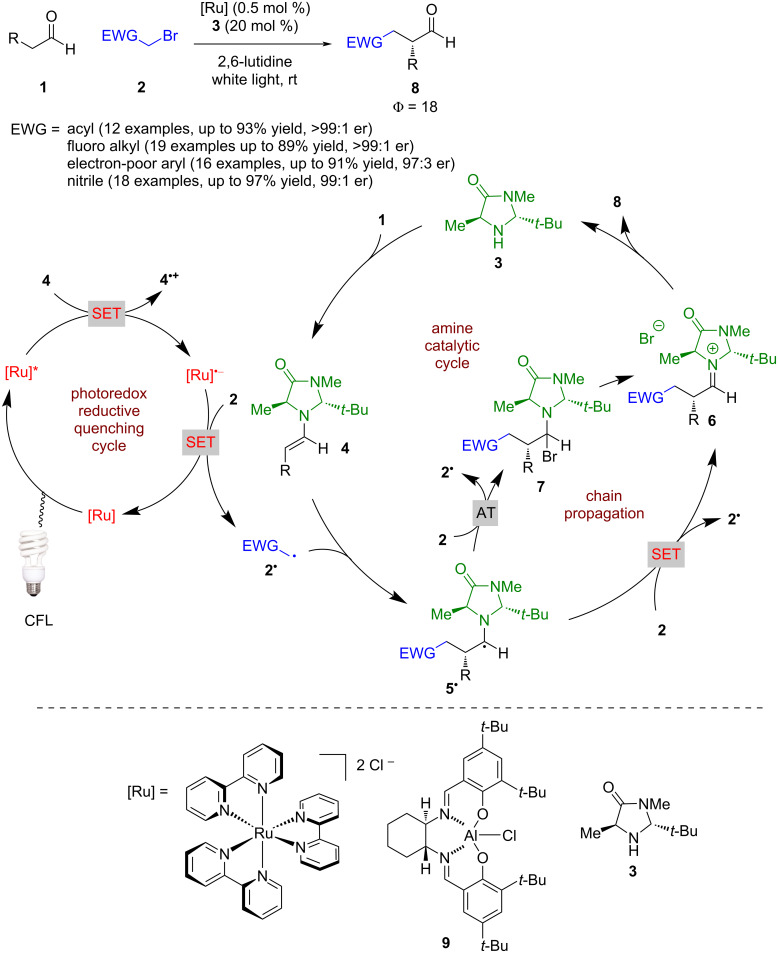
Amine/photoredox-catalysed α-alkylation of aldehydes with alkyl bromides bearing electron-withdrawing groups.

Much of the history of amine catalysis used in photochemical reactions can be found in a review published by Zou and Bach [12], so the following are selected examples and recent developments of the field. Amine catalysis can be broadly split into enamine and iminium catalysis, both of which have been utilised in combination with photocatalysis. The first example of enamine catalysis in combination with photoredox catalysis was reported by Nicewicz and MacMillan [1] for the alpha alkylation of aldehydes **1** with various alkyl bromides bearing an electron-withdrawing substituent **2**, which while seemingly trivial, was not possible with enamine catalysis alone (Scheme 1). The proposed mechanism suggests a closed catalytic cycle is in operation; however, subsequent investigations by Yoon [20] found this reaction has a quantum yield >1 (Φ = 18), which signifies a chain propagation process is dominant. Therefore, according to Yoon’s proposed mechanism, the reaction proceeds with the condensation of **1** with amine catalyst **3** to give enamine intermediate **4**. The initiation step is proposed to be a reductive quench of the photocatalyst using **4** as a sacrificial reductant to give [Ru]^•−^, which can then reduce **2** to give electrophilic radical **2^•^**. Addition of **2^•^** to another molecule of **4** generates α-amino radical **5^•^**, which (depending on the EWG) can either reduce another molecule of **2** via a SET process or via an atom transfer (AT) process and propagate the chain reaction [21]. The SET route directly generates **2^•^** and iminium ion intermediate **6**, but the AT goes through alkyl bromide **7**, before generating **6**. Hydrolysis of **6** furnishes the desired α-functionalised aldehydes **8** in excellent yields and enantioselectivities (12 examples, up to >99:1 er). Further work on this system expanded the scope to ketones [22] instead of aldehydes and varied the electron-withdrawing group to include fluorinated alkyl groups [23], electron-deficient arenes [24], and nitriles [25]. Additionally, Cozzi recently applied a novel aluminium-based photocatalyst **9** to this reaction, as an earth-abundant metal alternative albeit with slightly reduced enantioselectivities (8 examples, up to 96:4 er) [26]. Interestingly, as with some other photocatalysts used for this reaction, it is proposed the excited state of **9** is sufficiently reducing to initiate the chain mechanism through an oxidative quench.

**Scheme 2:**
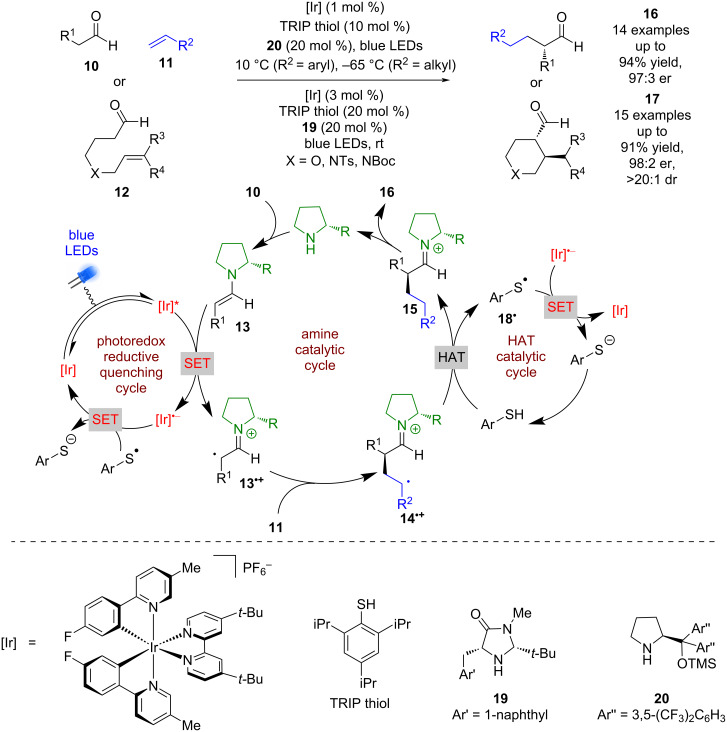
Amine/HAT/photoredox-catalysed α-functionalisation of aldehydes using alkenes.

Moving away from electronically activated halides, MacMillan et al. investigated a tricatalytic system, utilising enamine, photoredox, and HAT (hydrogen atom transfer) catalysis to allow the use of alkenes as the alkylating agent either in an intermolecular process using aldehydes **10** and alkenes **11** or intramolecularly using aldehydes **12** (Scheme 2) [27]. The proposed mechanism again proceeds via the formation of an enamine intermediate **13** that then reductively quenches the photocatalyst to form enaminyl radical **13^•+^**. However, in this reaction **13^•+^** can then add to the alkene to give an alkyl radical **14^•+^**, followed by hydrogen atom abstraction from the thiol, acting as a HAT catalyst, to give iminium ion intermediate **15**. Hydrolysis of **15** generates the desired α-functionalised aldehydes **16** (14 examples up to 97:3 er) or cyclization products **17** (15 examples up to 98:2 er) in excellent yields and enantioselectivities. A SET process between thiyl radical **18^•^** and [Ir]^•−^ is proposed to complete both catalytic cycles.

**Scheme 3:**
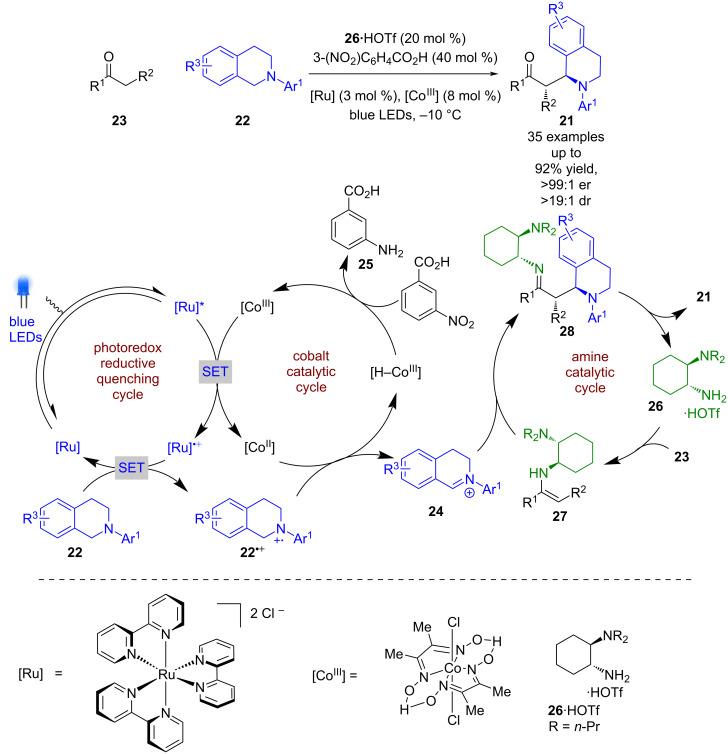
Amine/cobalt/photoredox-catalysed α-functionalisation of ketones and THIQs.

Another tricatalytic system developed by Tung et al. merged photoredox, cobalt, and amine catalysis towards the synthesis of α-functionalised ketones **21** from tetrahydroisoquinolines (THIQs) **22** and ketones **23** (Scheme 3) [28]. The proposed mechanism involves an oxidative quenching cycle using the [Co^III^] catalyst to generate [Co^II^] and [Ru]^•+^, with the latter oxidising **22** to give radical cation **22^•+^** and turn over the photocatalytic cycle. The radical cation **22^•+^** is then proposed to participate in a two-step electron and proton exchange process with [Co^II^] to give [H–Co^III^] and iminium ion **24**, likely via a [Co^I^] intermediate. [H–Co^III^] can then reduce 3-nitrobenzoic acid to the corresponding aniline **25** to turn over the cobalt cycle. Simultaneously, **23** condenses with the chiral primary amine catalyst **26** to give enamine intermediate **27**, which can be intercepted by **24** to generate imine intermediate **28**, which is finally hydrolysed to turn over the amine catalytic cycle and releases the desired products **21** in excellent yields and enantioselectivities (35 examples, up to >99:1 er). Notably, if **23** is acyclic the product enantioselectivities are poorer (2 examples up to 87:13 er).

**Scheme 4:**
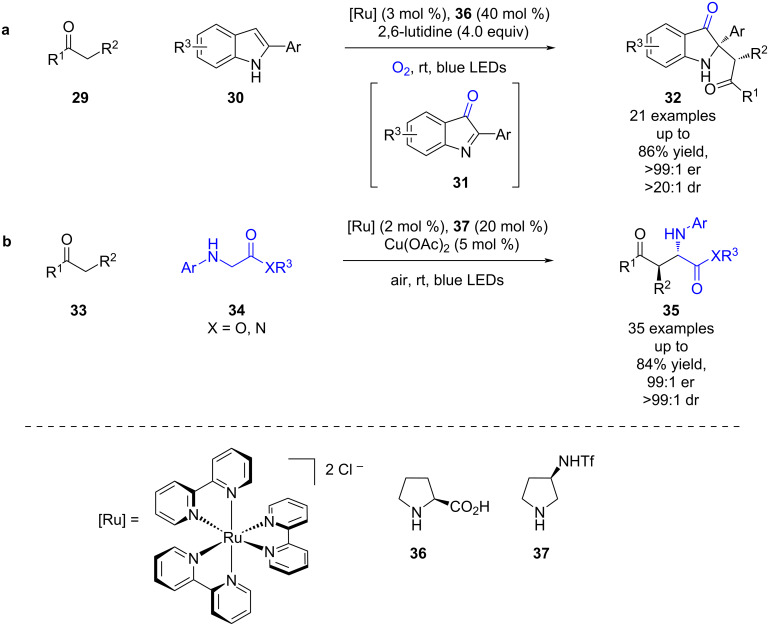
Amine/photoredox-catalysed α-functionalisation of aldehydes or ketones with imines. (a) Using ketones with 2-substituted indoles. (b) Using ketones or aldehydes with glycine derivatives.

A similar reaction was later reported by Guan et al. using ketones **29** with 2-substituted indoles **30** as a precursor to imine **31**, for the synthesis of indolin-3-ones **32** in good yields and excellent enantioselectivities (21 examples, up to >99:1 er) (Scheme 4a) [29]. Zhang et al. recently added to the scope of this family of reactions with their use of aldehydes/ketones **33** with glycine derivatives **34** to synthesise the corresponding products **35** in good yields and excellent enantioselectivities (35 examples, up to 99:1 er) (Scheme 4b) [30].

**Scheme 5:**
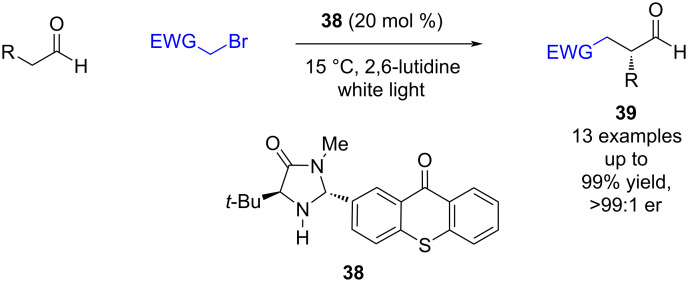
Bifunctional amine/photoredox-catalysed enantioselective α-functionalisation of aldehydes.

The previous examples of enamine/photoredox catalysis have all required two or more separate catalysts. Currently, three different approaches have been developed that use a single catalyst. Alemán et al.’s approach combines the two catalytic motifs into a single bifunctional catalyst **38**, using thioxanthone as the chromophore (Scheme 5) [31]. The catalyst **38** was then applied to known reactions such as the α-functionalization of aldehydes and gave excellent yields and enantioselectivities (13 examples, up to >99:1 er).

**Scheme 6:**
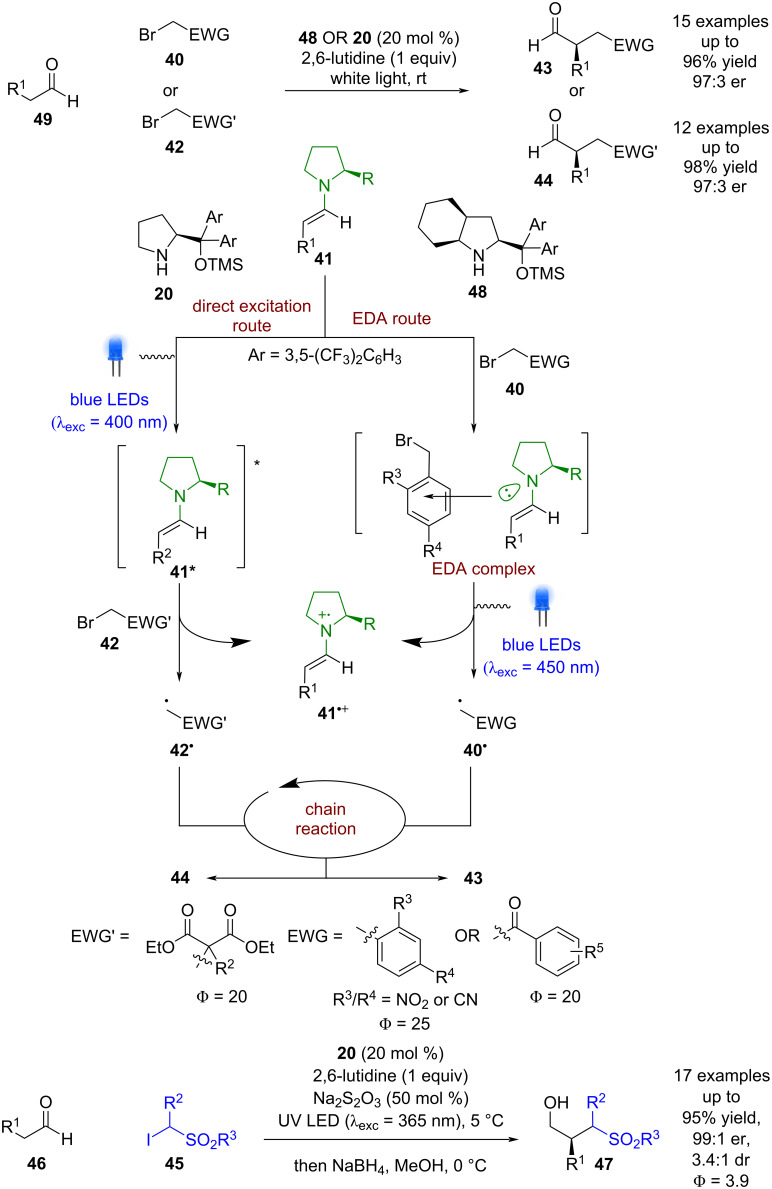
Bifunctional amine/photoredox-catalysed α-functionalisation of aldehydes using amine catalysts via EDA complexes and direct excitation of intermediates.

Another approach that used a single catalyst was developed by Melchiorre et al., where they discovered that a conventional photocatalyst was not necessary to achieve similar transformations (Scheme 6) [32–33]. When an alkylating agent containing an aryl ring **40** (e.g., phenacyl/benzyl bromides) is used, the enamine intermediate **41** forms a coloured electron-donor acceptor (EDA) complex that can absorb visible light via an intermolecular charge-transfer state (EDA route) [14,34]. Mechanistic investigations [21] showed that after excitation of the EDA complex, the electrophilic radical **40^•^** that is formed enters the same chain propagation cycle as in Scheme 1, whereas the radical cation **41^•+^** is proposed to be unstable and decomposes. The third approach, also developed by Melchiorre et al., was based on their observation of similar reactivity when using bromomalonates **42** as substrates (direct excitation route) [35]. As no aryl ring is present, no EDA complex is formed, and thus direct excitation of the photoactive enamine intermediate affords an excited state enamine 41* that can reduce **42** to initiate the chain reaction that produces the desired products **43** and **44** in excellent yields and enantioselectivities (15 examples, up to 97:3 er and 12 examples, up to 97:3 er, respectively). Subsequent progress using this methodology expanded the scope to the use of α-iodo sulfones **45**, which is proposed to proceed via the excited-state enamine [36].

**Scheme 7:**
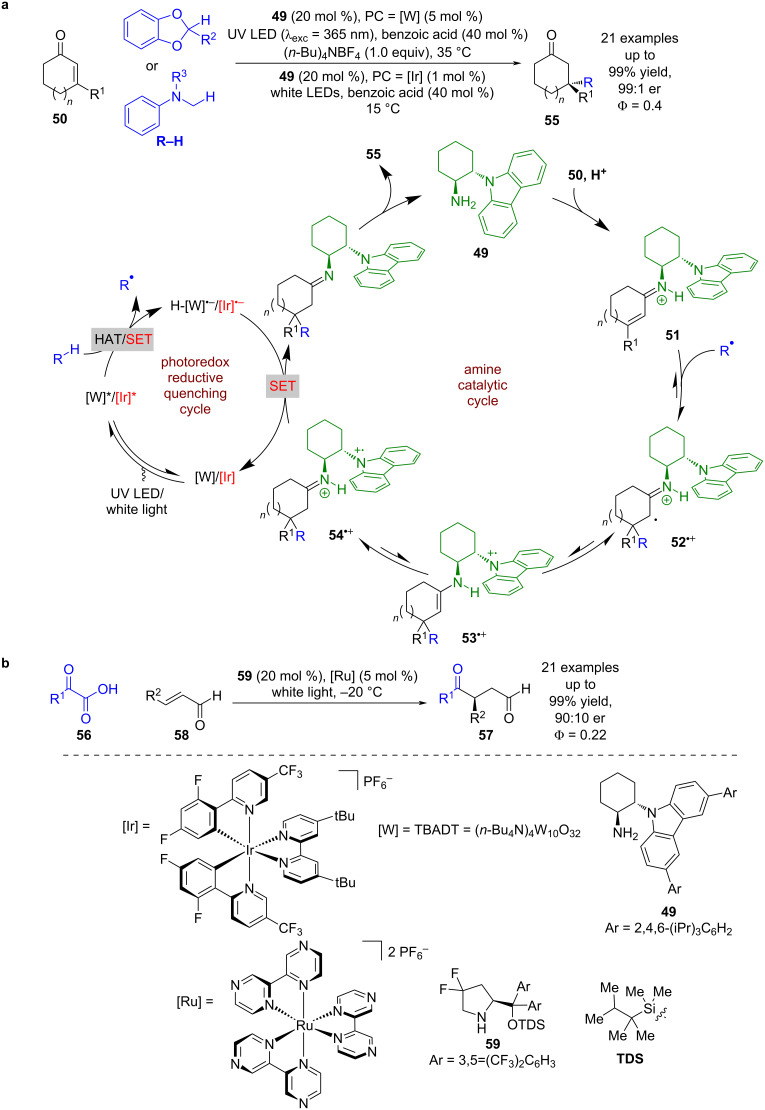
Amine/photoredox-catalysed RCA of iminium ion intermediates. (a) Synthesis of quaternary stereocentres using benzodioxole derivatives or tertiary amines. (b) Synthesis of 1,4-dicarbonyls using carboxylic acids.

While there is an abundance of known photoredox reactions that generate iminium ions catalytically either as a side product [37], or for further transformations [38], the use of iminium ions generated by chiral amines in combination with photoredox catalysts has been less explored. The first example was developed by Melchiorre et al. using a unique secondary amine catalyst containing a carbazole group **49** in combination with either tetrabutylammonium decatungstate (TBADT = [W]) or an iridium-based photocatalyst (Scheme 7a) [39]. The proposed mechanism begins with the condensation of **49** with enone **50** to form the iminium ion intermediate **51**. Concomitantly, the excited-state photocatalyst generates an alkyl radical R^•^ from R–H, either through HAT ([W] with a benzodioxole derivative) or SET ([Ir] with a tertiary amine). This radical then adds to the β-position of the iminium ion generating an unstable iminyl radical **52^•+^** that is quickly quenched by the nearby carbazole to form a more stable carbazole centred radical **53^•+^**. Rapid tautomerisation to imine **54^•+^** precludes the undesired back electron transfer. Single electron reduction of **54^•+^** by PC^•–^ and hydrolysis provides the radical conjugate addition (RCA) products **55** with a quaternary stereocentre in excellent yields and enantioselectivities (21 examples, up to 99:1 er). The quantum yield was measured to be <1 (Φ = 0.4) for the iridium-catalysed reaction, suggesting that a radical chain process is not dominant. Subsequent investigations by Yu expanded the scope to acyl radicals generated from carboxylic acids **56**, affording enantioenriched 1,4-carbonyls **57** in excellent yields and good enantioselectivities (21 examples, up to 90:10 er) (Scheme 7b) [40]. The quantum yield was also measured to be <1 (Φ = 0.22), so a chain mechanism is likewise unlikely.

**Scheme 8:**
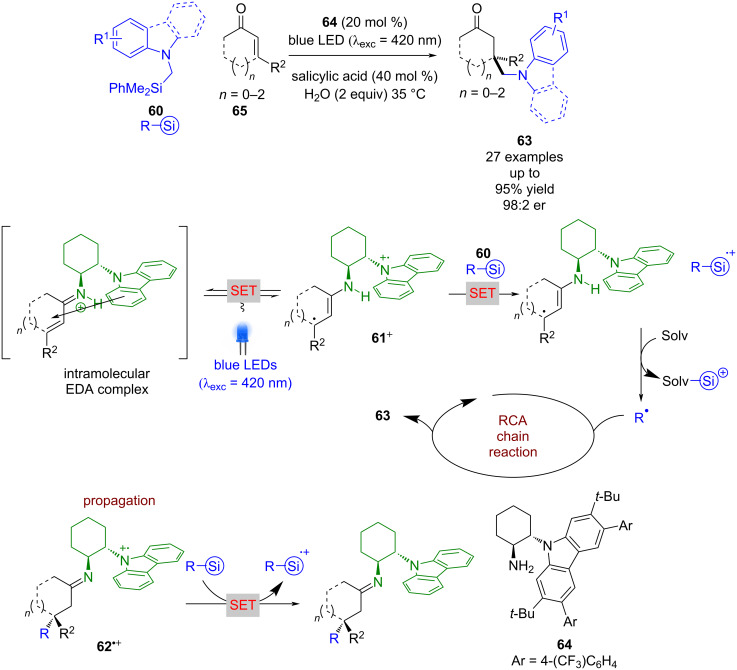
Bifunctional amine/photoredox-catalysed RCA of enones in a radical chain reaction initiated by an intramolecular EDA complex.

Interestingly, Melchiorre later found that with α-amino silanes **60**, no photocatalyst was required (Scheme 8) [41]. This was proposed to be due to the formation of an intramolecular EDA complex, which upon excitation can form cation **61^+^** that can subsequently oxidise **60** to give a nucleophilic radical R**^•^** that enters a similar RCA cycle as for Scheme 7. However, in the absence of an external photocatalyst, radical cation intermediate **62^•+^** is reduced by another molecule of **60**, thus propagating a radical chain mechanism that leads to the formation of ketones **63** in good yields and excellent enantioselectivities (27 examples, up to 98:2 er). The authors were unable to measure the quantum yield in this case due to difficulties with the high-power LED that is typically required.

**Scheme 9:**
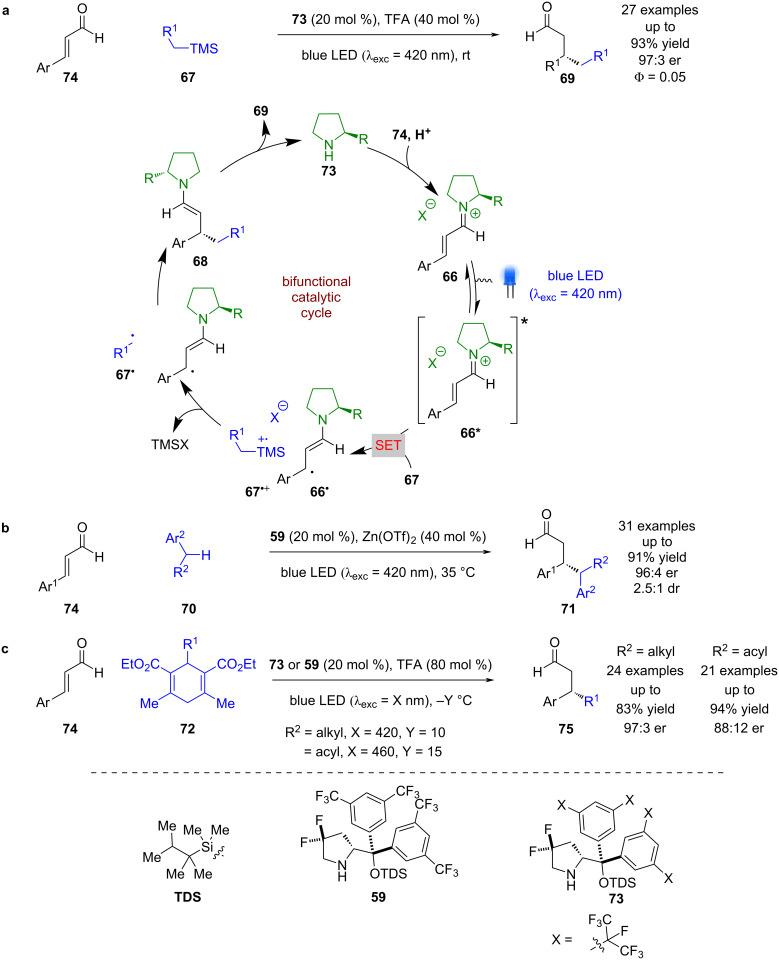
Bifunctional amine/photoredox-catalysed RCA reactions of iminium ions with different radical precursors. (a) Silanes. (b) Toluene derivatives. (c) Alkyl and acyl DHP derivatives.

As with the enamine intermediates, Melchiorre et al. also demonstrated that iminium ions **66** can be excited directly without formation of an EDA complex (Scheme 9a) [42]. The excited state iminium ion 66* can oxidise silanes **67** via a SET process to give radical cation **67^•+^** and alkyl radical **66^•^**. Loss of the TMS group generates alkyl radicals **67^•^** that can couple with **66^•^** enantioselectively to give enamine intermediate **68**, which after hydrolysis completes the catalytic cycle and releases the desired RCA products **69** in good yields and enantioselectivities (27 examples, up to 97:3 er). The quantum yield was determined to be <1 (Φ = 0.05) so a chain reaction is unlikely to be dominant. This reactivity was later extended to the use of toluene derivatives **70** to generate the corresponding RCA products **71** in good yields and enantioselectivities (31 examples, up to 96:4 er) (Scheme 9b) [43]. Melchiorre et al. also discovered that dihydropyridine (DHP) derivatives **72** could act as efficient radical precursors in this system, allowing for both alkyl [44] and acyl [45] RCA reactions. In the case of acyl DHPs, they propose that direct excitation of the DHP leads to radical generation rather than the iminium intermediate (Scheme 9c).

**Scheme 10:**
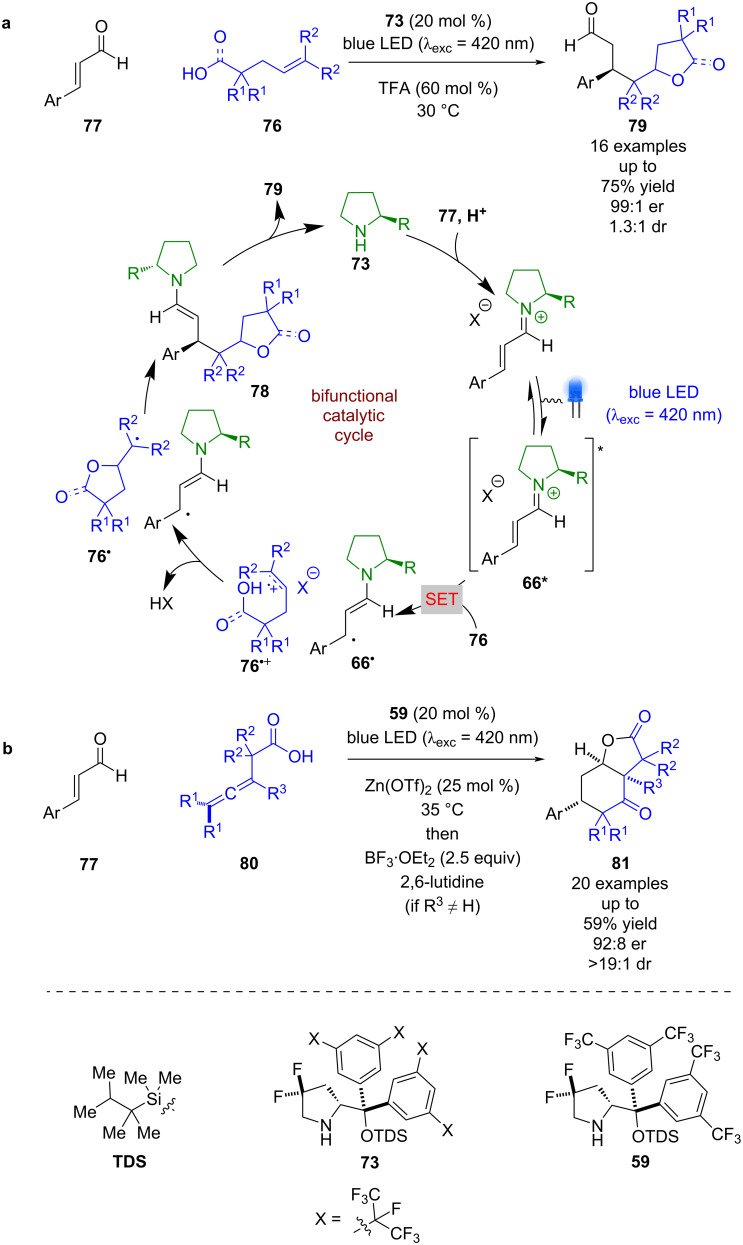
Bifunctional amine/photoredox-catalysed radical cascade reactions between enones and alkenes with attached nucleophilic groups. (a) Alkenes with attached carboxylic acid or alcohol. (b) Allenes with attached carboxylic acid.

The same system was used for the radical cascade reaction between carboxylic acid/alcohol **76** and enal **77** (Scheme 10a) [46]. Analogously to the mechanism outlined in Scheme 9, it was proposed that formation of excited state iminium ion 66* is used to oxidise **76** to give radical cation **76^•+^** and alkyl radical **66^•^**. Nucleophilic addition from the carboxylic acid or alcohol gives neutral radical **76^•^**, which couples enantioselectively with **66^•^** to give enamine intermediate **78**. Subsequent condensation releases the photocatalyst and the desired products **79** in good yields and excellent enantioselectivities (16 examples, up to 99:1 er). Recently, this process was extended to allenes **80** to give complex bicyclic products **81** in moderate yields and good enantioselectivities (20 examples, up to 92:8 er) (Scheme 9b) [47].

**Scheme 11:**
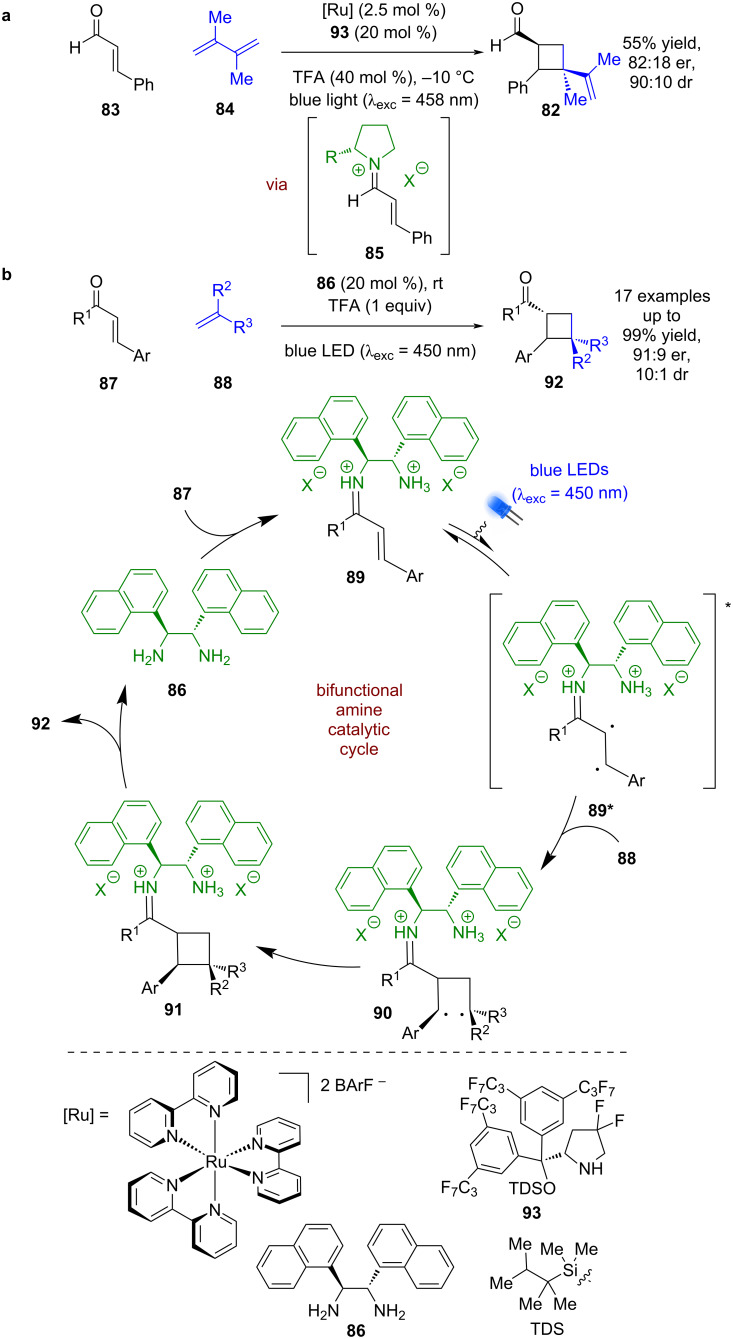
Amine/photocatalysed photocycloadditions of iminium ion intermediates. (a) External photocatalyst used as triplet sensitiser with enals and dienes. (b) Bifunctional amine catalyst used with enones and alkenes.

The Bach group recently developed an enantioselective synthesis of cyclobutane **82** from enal **83** and diene **84** (Scheme 11a) [48]. Most of the examples proceed via a stepwise approach with preformation of the corresponding iminium ion **85**. The only catalytic example achieves the same transformation by generating **85** in situ but has reduced yields and enantioselectivities (82:18 er vs 92:8 er for stepwise). Alemán et al. reported that a similar reaction could proceed catalytically and with a broad scope using amine catalyst **86** with enones **87** and alkenes **88** without the need for an external photocatalyst (Scheme 11b) [49]. The mechanism proposed by Alemán begins with the condensation of **86** with **87** to generate iminium ion **89**, which has a suitably low energy charge transfer state that can be photoexcited to generate singlet intermediate 89*. Subsequent enantioselective photocycloaddition with **88** via diradical **90** gives iminium ion intermediate **91**, which after hydrolysis affords the desired cyclobutane products **92** in excellent yields and good enantioselectivities (17 examples, up to 91:9 er). Bach proposes for their reaction that an external ruthenium photocatalyst generates the triplet excited state iminium ion through an energy transfer process, which is also observed by Alemán when using an external transition metal-based sensitiser.

**Scheme 12:**
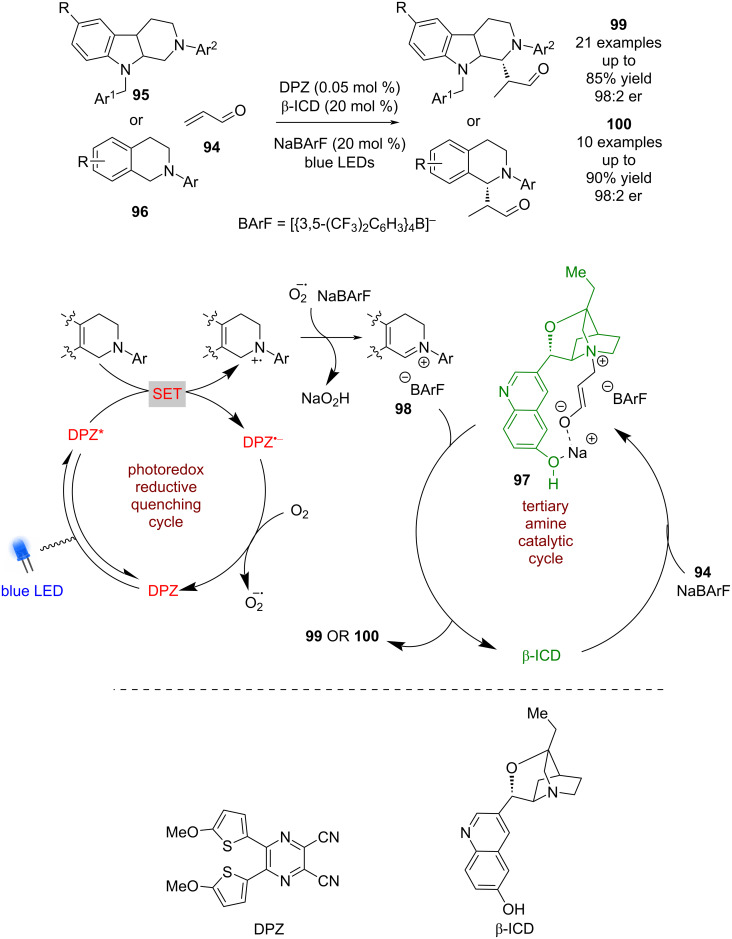
Amine/photoredox-catalysed addition of acrolein (**94**) to iminium ions.

Tertiary amine catalysts such as β-isocupreidine (β-ICD) have found limited use in combination with photoredox catalysis, likely due to their tendency to oxidise to form iminium ions. However, Jiang et al. have developed a process using acrolein (**94**) in the presence of tetrahydro-β-carbolines (THCs) **95** or THIQs **96** and a dicyanopyrazine-derived (DPZ) photocatalyst (Scheme 12) [50]. They propose that addition of β-ICD to acrolein is assisted by NaBArF to give a zwitterionic intermediate **97**, which is then intercepted by the photocatalytically generated iminium ion **98**, followed by loss of β-ICD to give enantioenriched products **99** or **100** in good yields and enantioselectivities (21 examples for THCs, up to 98:2 er and 10 examples for THIQs, up to 98:2 er).

### N-Heterocyclic carbene catalysis

**Scheme 13:**
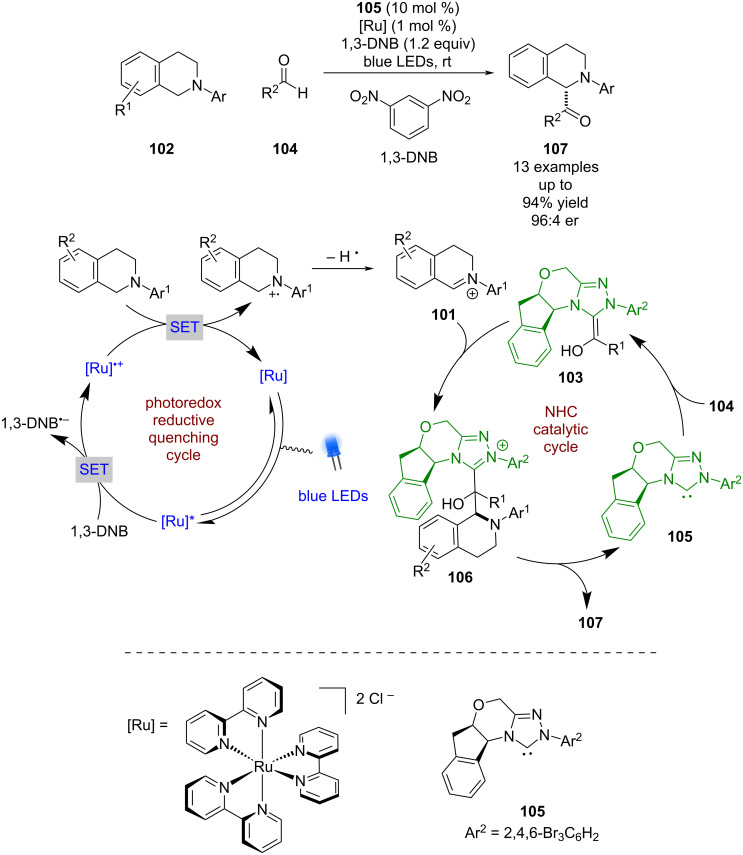
Dual NHC/photoredox-catalysed acylation of THIQs.

N-Heterocyclic carbene (NHC) catalysis was first used in combination with photoredox catalysis by Rovis in 2012. They showed that iminium ions **101** could be generated in an oxidative quenching cycle from THIQs **102** using a ruthenium-based photocatalyst and 1,3-dinitrobenzene (DNB) as a sacrificial oxidant (Scheme 13) [51]. These iminium ions could then be intercepted by a Breslow intermediate **103**, formed between aldehydes **104** and the NHC catalyst **105**, to generate intermediate **106**, which can then turn over the NHC and release the desired acylated products **107** in good yields and enantioselectivities (13 examples, up to 96:4 er).

**Scheme 14:**
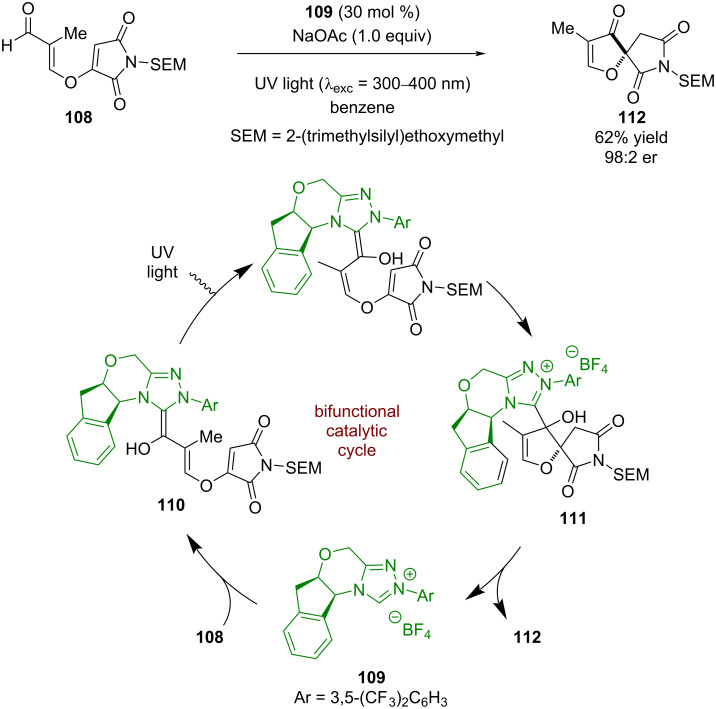
NHC/photocatalysed spirocyclisation via photoisomerisation of an extended Breslow intermediate.

There has been little development of enantioselective reactions using NHCs in photocatalysis since this work. Another example was reported by Rovis in 2013 using enal **108** in the presence of chiral NHC **109** to form extended Breslow intermediate **110** (Scheme 14) [52]. Photoisomerisation of **110** is then required for the following spirocyclisation reaction to intermediate **111** to proceed, which then releases the NHC catalyst and intermediate **112** for the synthesis of (–)-cephalimysin A in moderate yield and excellent enantioselectivity (98:2 er). Interestingly, there have been multiple reports of racemic reactions that combine photoredox and NHC catalysis [53–56], but few enantioselective examples, suggesting there is much progress yet to be made.

#### Brønsted acid catalysis

**Scheme 15:**
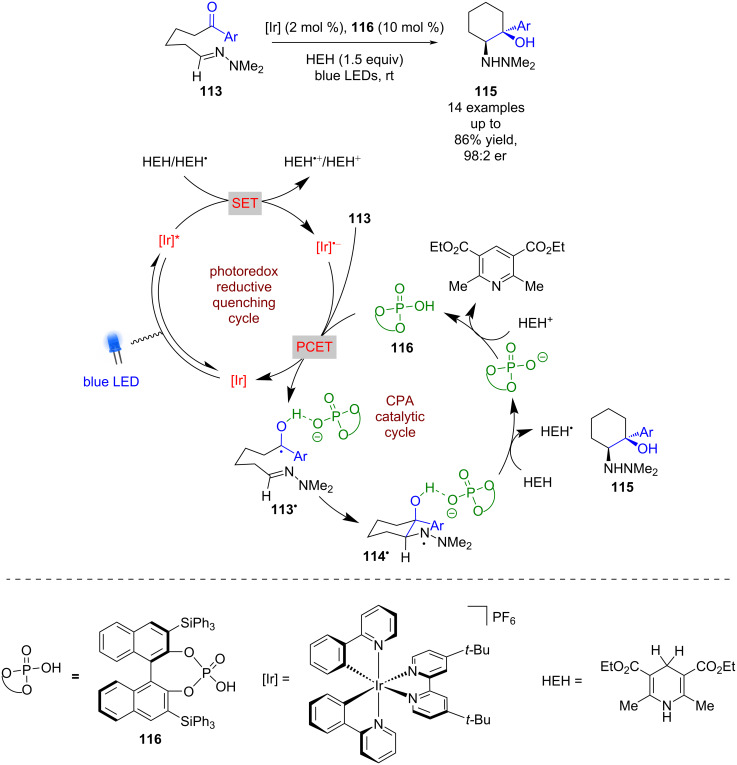
CPA/photoredox-catalysed aza-pinacol cyclisation.

Using chiral amines and NHCs as catalysts to generate asymmetry relies upon the formation of covalently bonded intermediates such as enamines, iminium ions or Breslow intermediates within the catalytic cycle. The first example of merging non-covalent catalysis with photoredox catalysis was reported by Rono and Knowles in 2013 (Scheme 15) [57]. They showed that using a chiral phosphoric acid (CPA), a photoredox catalyst and Hantzsch ester (HEH) as a HAT reagent, a concerted proton-coupled electron transfer (PCET) process is promoted to form ketyl radicals **113^•^**, which, in the presence of a hydrazone, cyclises to give *N*-centred radical **114^•^**. Subsequent HAT from HEH furnishes aza-pinacol product **115** in good yields and excellent enantioselectivities (14 examples, up to 98:2 er).

**Scheme 16:**
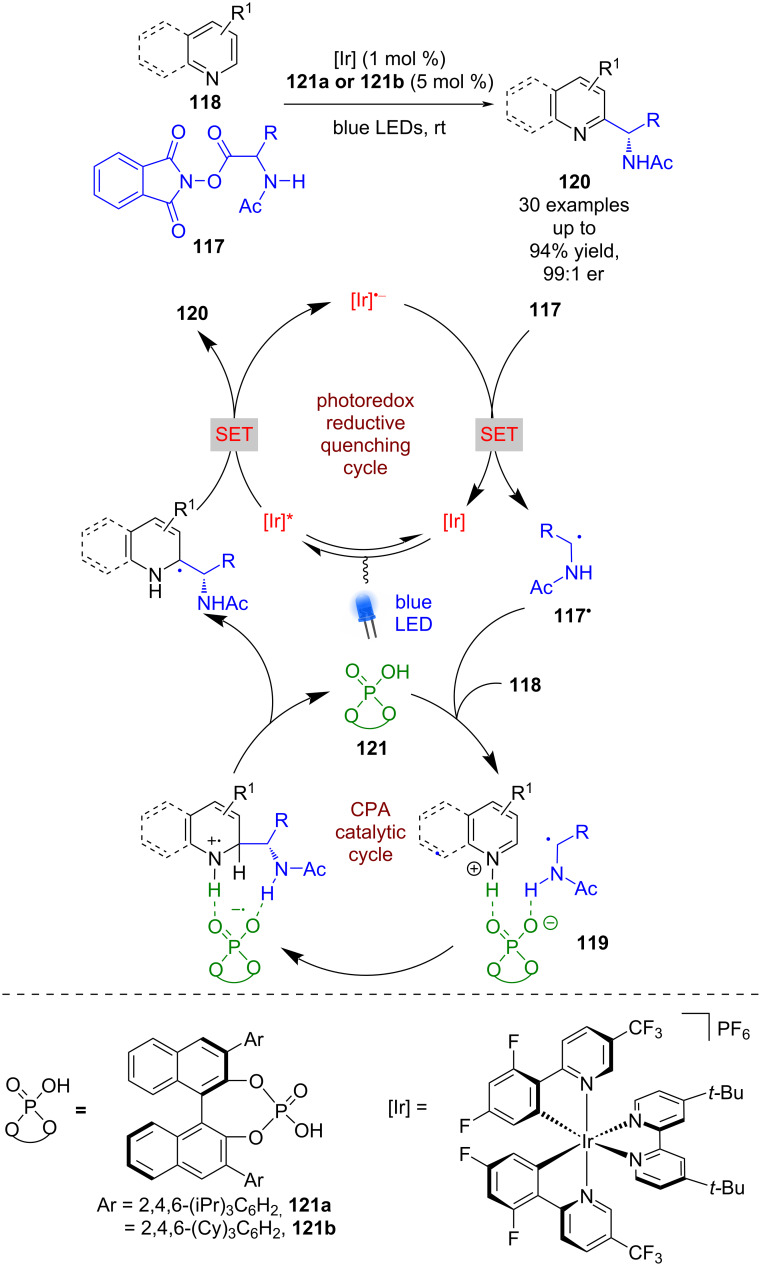
CPA/photoredox-catalysed Minisci-type reaction between azaarenes and α-amino radicals.

Since this initial report, a variety of processes have been developed using CPAs in combination with photoredox catalysis [58]. A significant contribution came from the Phipps group with their work on enantioselective Minisci-type reactions (Scheme 16) [59]. Here, the CPA acts as a sacrificial reductant, with the photocatalyst proceeding through a reductive quenching cycle, generating [Ir]^•−^, which reduces the phthalimide ester **117** to give α-amino radicals **117^•^** after decarboxylation. The CPA then activates the azaarene **118** through protonation and brings the two reactive species together in a hydrogen bonded complex **119**, which facilitates radical addition. After deprotonation and oxidation via SET, both catalysts are regenerated and Minisci-type products **120** are released in excellent yields and enantioselectivities (30 examples, up to 99:1 er). Pyridyl substrates are tolerated, but generally required the presence of electron-withdrawing groups for the reaction to proceed.

**Scheme 17:**
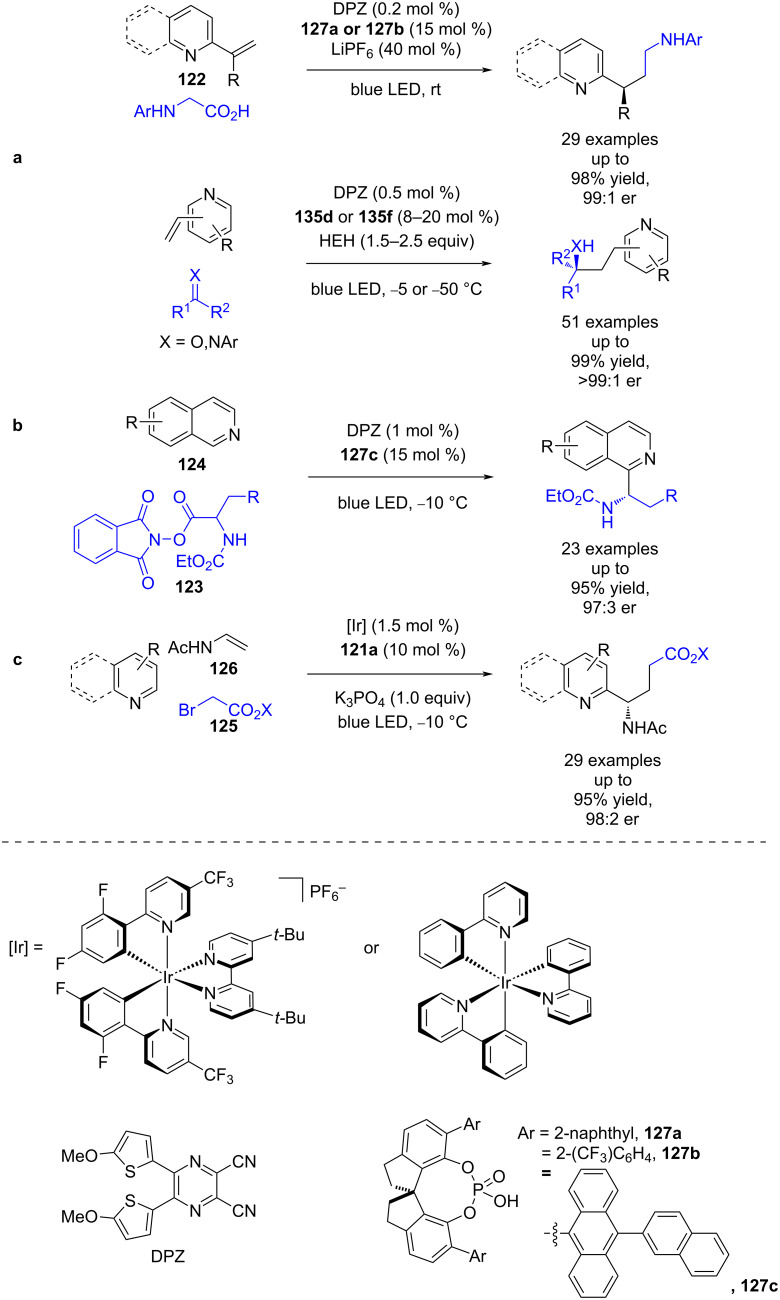
CPA/photoredox-catalysed radical additions to azaarenes. (a) α-Amino radical or ketyl radical addition to 2-vinylazaarenes. (b) Minisci-type reaction using redox-active esters and isoquinolines. (c) Radical cascade reaction using α-carbonyl radicals with enamides.

Jiang et al. developed a similar system for the enantioselective radical addition into 2-vinylazaarenes **122** using DPZ and either α-amino radicals [60] or ketyl radicals [61], with pyridines being well tolerated as substrates in this latter case (Scheme 17a). The same group also separately developed a Minisci-type reaction using phthalimide esters **123**; however, their system did not extend past isoquinoline substrates **124** (Scheme 17b) [62]. Zheng and Studer expanded the scope of this type of reactivity to a three-component cascade reaction with alkyl bromides **125** and enamides **126** (Scheme 17c) [63]. Interestingly, they found some of their examples required the more strongly reducing photocatalyst Ir(ppy)_3_ to achieve high yields, although the reason for this is unclear.

**Scheme 18:**
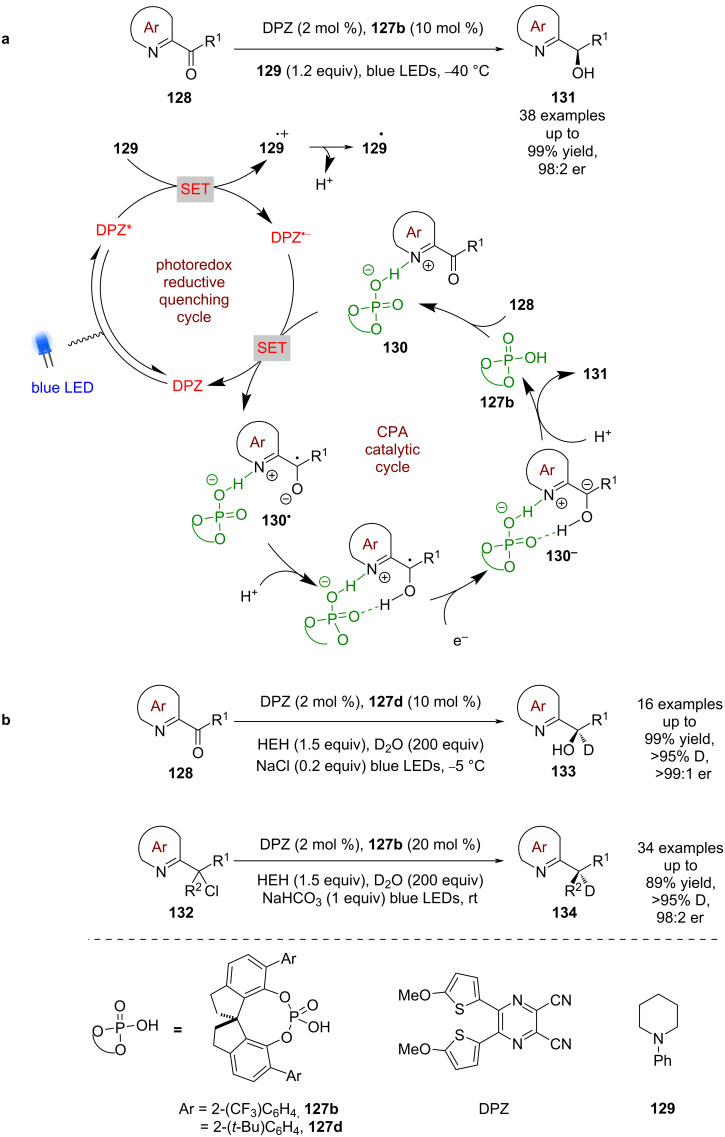
CPA/photoredox-catalysed reduction of azaarene-derived substrates. (a) Reduction of ketones. (b) Extension to deuteration of ketones and alkyl halides.

Jiang et al. continued to explore the reactivity landscape of CPAs and azaarenes, demonstrating that ketone **128** could be reduced enantioselectively (Scheme 18a) [64]. The proposed mechanism in this case also follows a reductive quenching cycle using DPZ, but this time includes a tertiary amine **129** as a sacrificial reductant to generate DPZ^•−^. As in the previous examples, the azaarene nitrogen is proposed to be protonated by the CPA to form chiral ion pair **130**, which is then reduced by DPZ^•−^ to give ketyl radical **130^•^**. The radical **130^•^** is then reduced further to the carbanion **130^−^**, which is protonated enantioselectively to give alcohols **131** in excellent yields and enantioselectivities (38 examples, up to 98:2 er). It is notable that pyridyl ketones do not perform well in this reaction (1 example, 70:30 er). A further extension of this methodology includes the deuteration of **128** and alkyl halides **132** using D_2_O to afford **133** (18 examples, up to >99:1 er) and **134** (34 examples, up to 98:2 er), respectively (Scheme 18b) [65].

**Scheme 19:**
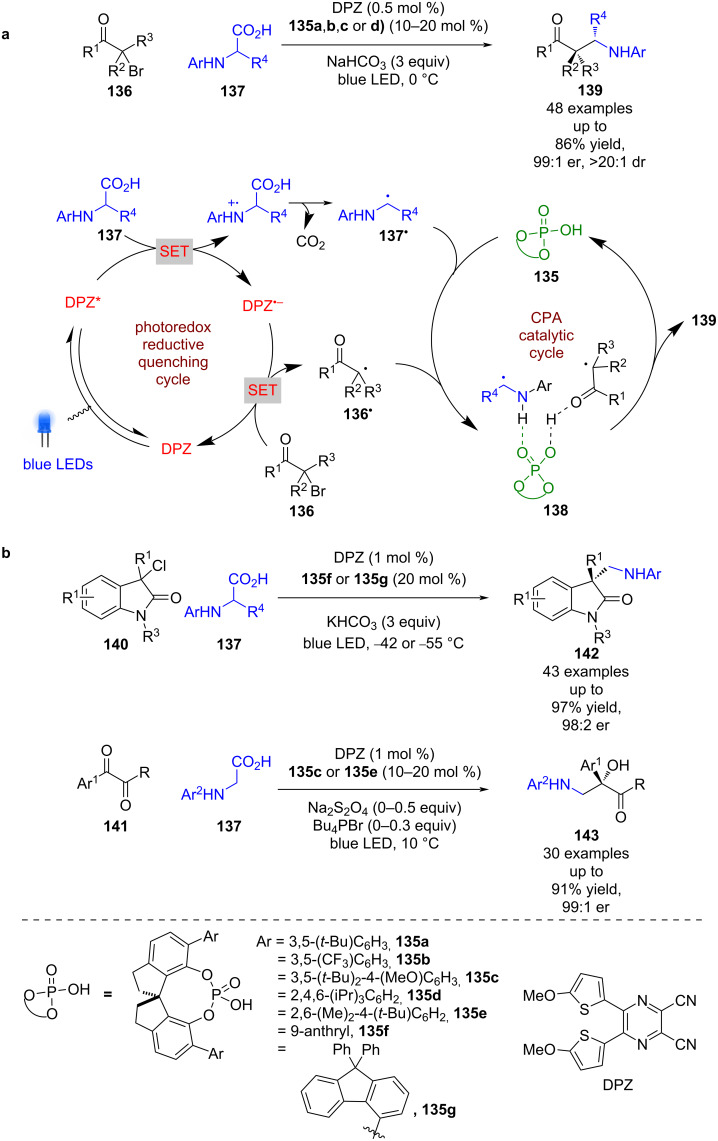
CPA/photoredox-catalysed radical coupling reactions of α-amino radicals with α-carbonyl radicals. (a) Using α-bromoketones. (b) Using α-chloro-2-oxindoles or 1,2-diketones.

Jiang et al. also reported a series of radical coupling reactions using different CPAs **135a–g** and DPZ, with the first examples using α-bromoketones **136** and α-amino acids **137** as radical precursors (Scheme 19a) [66]. The proposed mechanism proceeds through a reductive quenching cycle to generate α-amino radical **137^•^** and DPZ^•−^, which can reduce **136** to give α-carbonyl radical **136^•^**. The CPA is then proposed to form a ternary hydrogen-bonded complex **138** to mediate enantioselective radical coupling that furnishes the desired products **139** in good yields and excellent enantioselectivities (48 examples, up to 99:1 er). This methodology was later expanded to 2-oxindoles **140** [67] and 1,2-diketones **141** [68] to form the corresponding radical coupling products **142** (43 examples, up to 98:2 er) and **143** (30 examples, up to 99:1 er), respectively, in comparable yields and enantioselectivities to those of **139** (Scheme 19b).

**Scheme 20:**
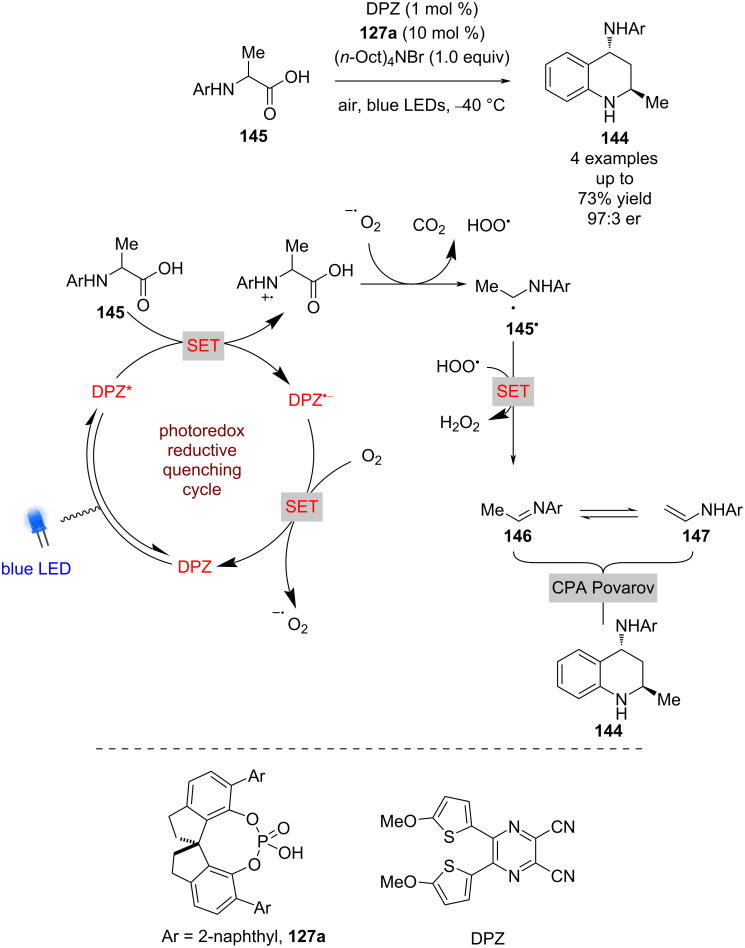
CPA/photoredox-catalysed Povarov reaction.

As previously mentioned, photoredox catalysis has been widely used for the generation of imines. The Jiang group applied this to the synthesis of substituted tetrahydroquinolines (THQs) **144** from α-amino acids **145** to give racemic products in good yields and found that the addition of a CPA provided good enantioselectivity (Scheme 20) [69]. The putative mechanism proceeds via a reductive quenching cycle to give α-amino radical **145^•^** after decarboxylation, which is then oxidised further to the imine **146** in the presence of oxygen. Imine **146** is in equilibrium with the enamine tautomer **147**, and the CPA-catalysed Povarov reaction between them gives the enantioenriched THQ **144** in good yields and enantioselectivities (4 examples, up to 97:3 er).

**Scheme 21:**
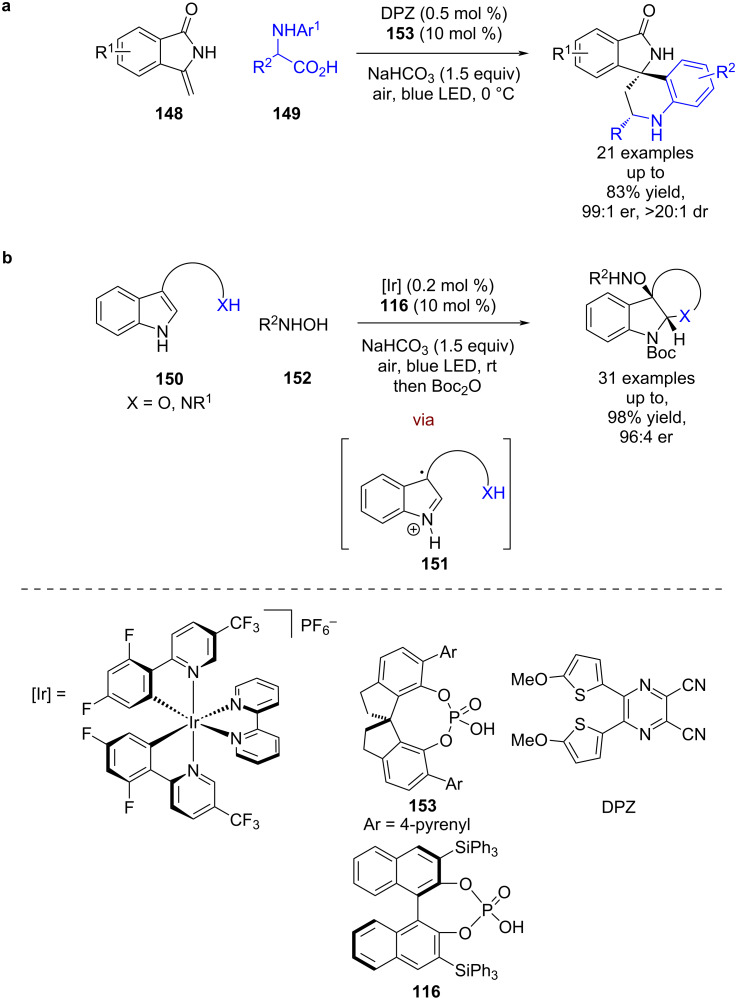
CPA/photoredox-catalysed reactions with imines. (a) Decarboxylative imine generation followed by Povarov reaction with an enamide. (b) Imine trapped by intramolecular nucleophile and intermolecular nucleophile.

This reactivity has been extended to enamides **148**, with the imine generated from the α-amino acid **149** now reacting with **148** in a CPA-catalysed Povarov reaction rather than with its own tautomer (Scheme 21a) [70]. Zhang and You developed a catalytic dearomatisation reaction of indoles **150** using similar chemistry, where the generated imine **151** now reacts intramolecularly with a pendant nucleophile and is further oxidised to a carbocation that is trapped intermolecularly by *N*-hydroxycarbamates **152** (Scheme 21b) [71].

**Scheme 22:**
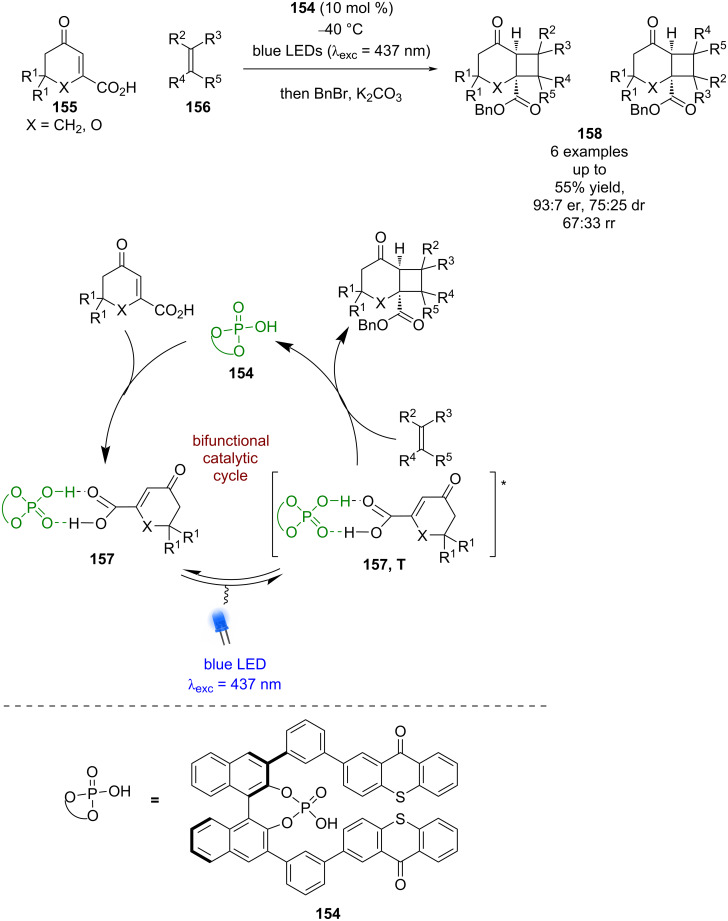
Bifunctional CPA/photocatalysed [2 + 2] photocycloadditions.

Bach et al. has recently reported a bifunctional catalyst **154**, which contains both a photoactive thioxanthone unit and a CPA (Scheme 22) [72]. They have applied this photocatalyst to the [2 + 2] photocycloaddition of carboxylic acids **155** with alkenes **156**. A low yielding benzylation reaction was required for determination of enantioselectivities and a large excess of alkene was required for the reaction. The reaction is proposed to proceed via hydrogen-bonded complex **157**, that lowers the triplet energy of the carboxylic acid so that a Dexter energy transfer process is possible from the photocatalyst to the substrate to promote **155** into its triplet state, which can then cyclise with **156** to give enantioenriched cycloaddition products **158**. The selectivity of this reaction is generally low (6 examples, up to 93:7 er, 67:33 rr) but this example does demonstrate an interesting proof of concept with potential for the development of processes using alternative bifunctional catalysts.

#### Phase-transfer catalysis

**Scheme 23:**
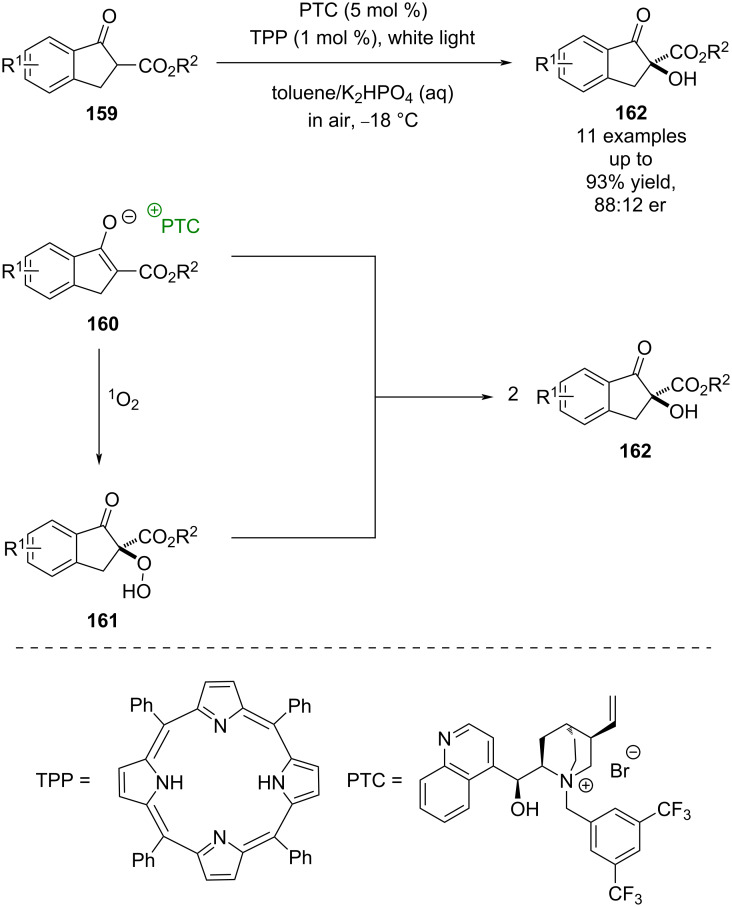
PTC/photocatalysed oxygenation of 1-indanone-derived β-keto esters.

Phase-transfer catalysis (PTC) is another non-covalent mode of catalysis that has been combined with photocatalysis. The first example of PTC being used in combination with photocatalysis was developed by Gao towards the oxygenation of 1-indanone derived β-keto esters **159** (Scheme 23) [73]. Gao proposes tetraphenylporphyrin (TPP) acting as a sensitiser to form ^1^O_2_ via photoinduced energy transfer, which is then trapped by enolate **160** in a chiral environment, provided by the PTC, to form a α-hydroperoxyl intermediate **161**. Subsequent deoxygenation by another molecule of **160** forms two molecules of the α-hydroxylated products **162** in excellent yields and moderate enantioselectivities (11 examples, up to 88:12 er). The selectivity has been improved further with the development of new PTCs [74–76], although the scope of the reaction remains limited.

**Scheme 24:**
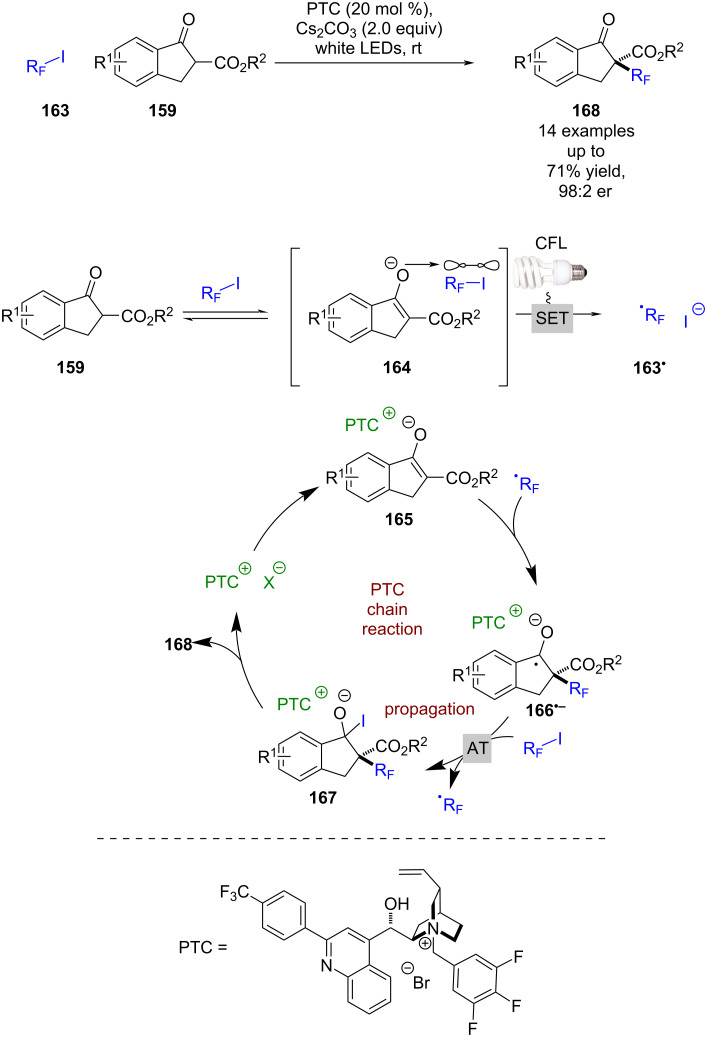
PTC/photoredox-catalysed perfluoroalkylation of 1-indanone-derived β-keto esters via a radical chain reaction initiated by an EDA complex.

Building on these initial reports, Melchiorre et al. developed a perfluoroalkylation reaction of the same starting materials **159** using alkyl iodides **163** (Scheme 24) [77]. Interestingly, this takes place without the need for an external photocatalyst and is proposed to proceed via an intermediate EDA complex **164**, which, upon excitation, forms perfluoroalkyl radicals **163^•^** that add to the enolate substrate that is coordinated to a chiral counterion **165** to give ketyl radical anion **166^•^**^−^. The latter intermediate **166^•^**^−^ can then abstract an iodine atom from another molecule of **163** to propagate the chain reaction and generate alkyl iodide **167**, which collapses to give the desired enantioenriched alkylation products **168** in moderate yields and excellent enantioselectivities (14 examples, up to 98:2 er). The quantum yield of the enantioselective reaction could not be ascertained due to the reaction being heterogenous, but the quantum yield for the racemic variant using an achiral base was >1 (Φ = 1.2), supporting the proposed radical chain process. While these results are promising for this mode of catalysis, more work is required to address the limited substrate scope.

#### Hydrogen bonding

**Scheme 25:**
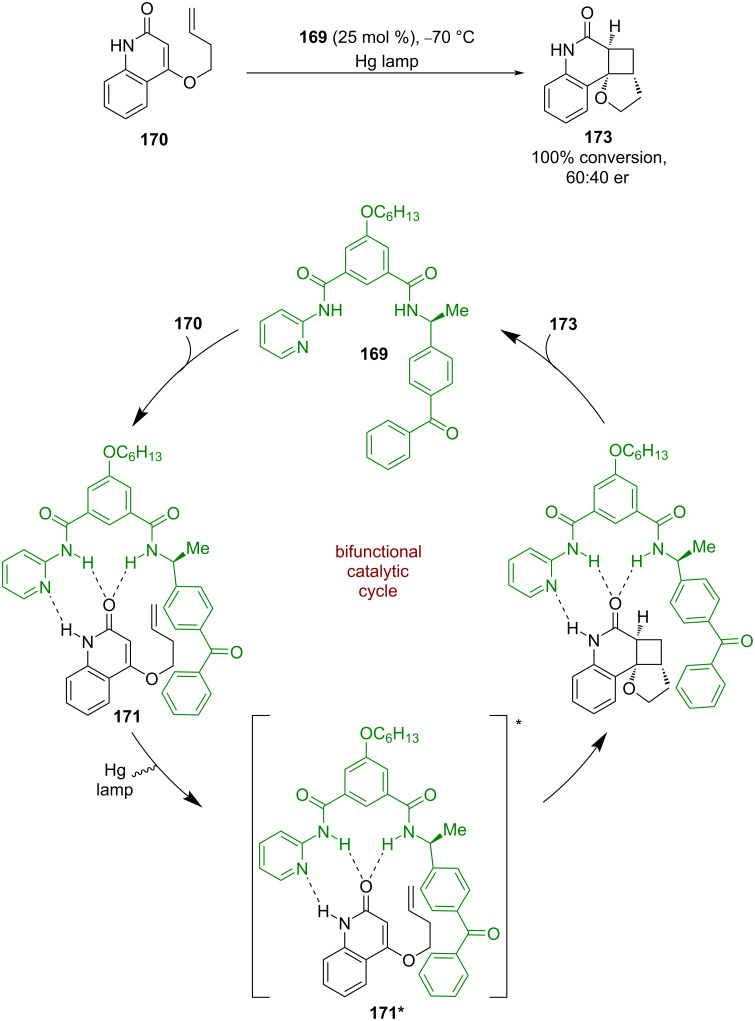
Bifunctional hydrogen bonding/photocatalysed intramolecular [2 + 2] photocycloadditions of quinolones.

Krische et al. were the first to develop a bifunctional hydrogen bonding photocatalyst **169** (Scheme 25) [78] that was used in an intramolecular enantioselective [2 + 2] photocycloaddition of quinolone **170**. The proposed mechanism proceeds via hydrogen-bonded complex **171**, which is sensitised by the pendant benzophenone to its triplet excited state 171*. The following cycloaddition completes the cycle and generates the desired cyclobutane product **173** in excellent conversion but poor enantioselectivity (60:40 er). While the enantioselectivity was low, this reaction represented an interesting proof of concept that would be later expanded by others.

**Scheme 26:**
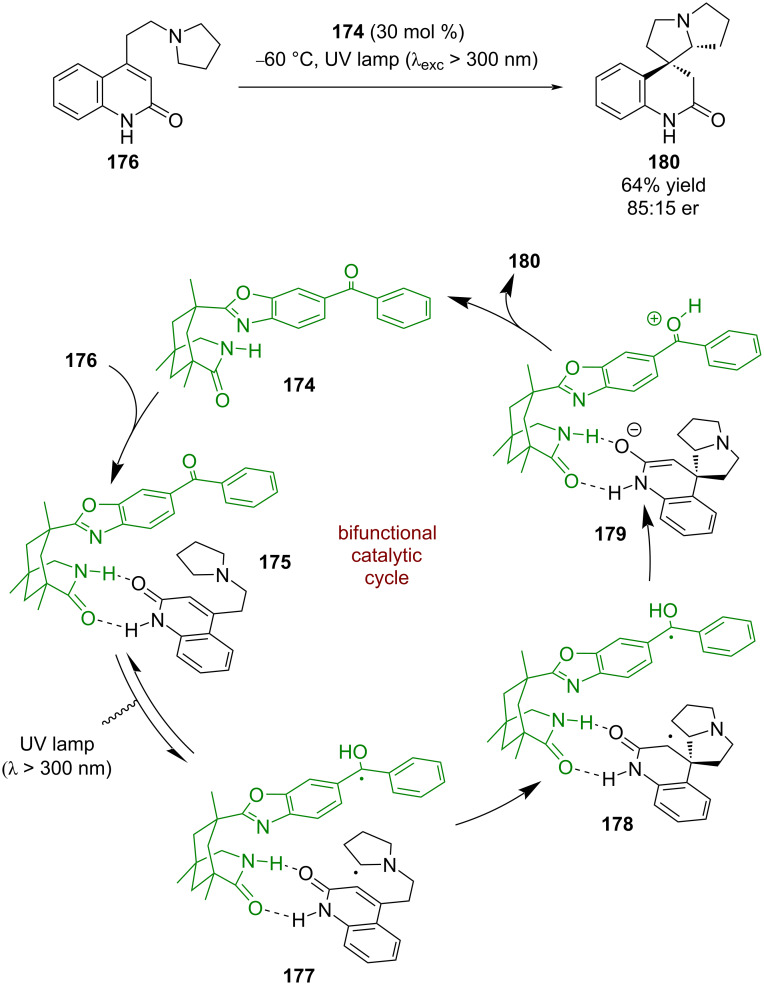
Bifunctional hydrogen bonding/photocatalysed intramolecular RCA cyclisation of a quinolone.

Later, Bach et al. developed a similar bifunctional hydrogen bonding photocatalyst **174**. Reactions using catalysts of this type are well covered in Bach’s recent review on the subject [13]. Photocatalyst **174** was first used in a cyclisation reaction where the putative mechanism involves a hydrogen bonding complex **175** between the catalyst and quinolone substrate **176** (Scheme 26) [2]. Subsequent photoexcitation promotes a photoinduced electron transfer to generate diradical **177** that then adds to the alkene to form diradical **178**. A SET between the ketyl radical and the α-carbonyl radical generates enolate intermediate **179**, which after proton transfer regenerates the catalyst and releases the desired cyclisation product **180** in a moderate yield and enantioselectivity (85:15 er).

**Scheme 27:**
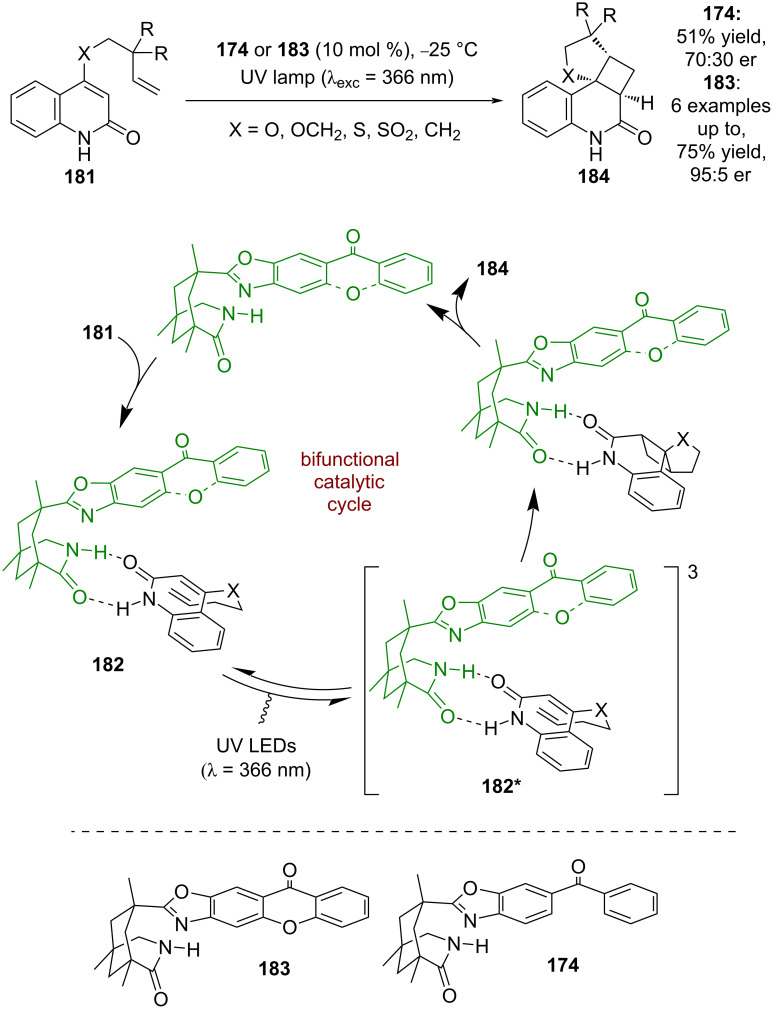
Bifunctional hydrogen bonding/photocatalysed intramolecular [2 + 2] photocycloadditions of quinolones.

Photocatalyst **174** was next applied to the formal [2 + 2] photocycloaddition of quinolones **181**, which is analogous to the reaction developed by Krische et al. in Scheme 25. Similarly, the mechanism that is proposed proceeds through complex **182** that can be photosensitised into its triplet excited state 182* (Scheme 27) [79–80]. However, the enantioselectivities for this reaction were poor (70:30 er), which prompted further catalyst design by changing the photosensitising group from benzophenone to xanthone **183** to improve enantioselectivity [79]. Xanthone has a higher triplet energy than benzophenone (3.2 eV vs 3.0 eV) and the authors attribute this difference to the increased efficiency; however, the efficiency of energy transfer is governed by spectral overlap between donor and acceptor. The much higher enantioselectivities observed (95:5 er) are attributed to a reduced amount of background reaction and the more rigid xanthone structure acting as a superior stereo-directing group.

**Scheme 28:**
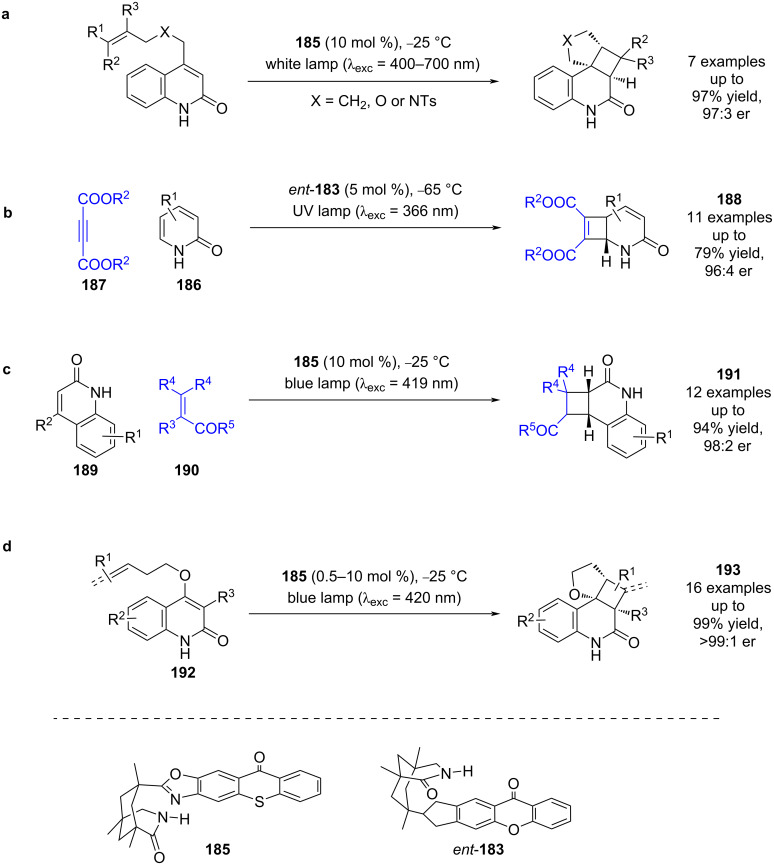
Bifunctional hydrogen bonding/photocatalysed [2 + 2] photocycloaddition reactions. (a) First use of the thioxanthone-based bifunctional catalyst for an intramolecular cycloaddition. (b) Intermolecular cycloaddition of pyridines and acetylenedicarboxylates using a xanthone-based photocatalyst. (c) Intermolecular cycloaddition of quinolones with electron-deficient alkenes using a thioxanthone-based photocatalyst. (d) Intramolecular cycloaddition of quinolones with attached alkenes or allenes using a thioxanthone-based photocatalyst.

Further variation to a thioxanthone unit **185**, which has a lower triplet energy (*E*_T_ = 2.7 eV), was used to investigate a similar [2 + 2] photocycloaddition, giving comparable yields and enantioselectivities (7 examples, up to 97:3 er) (Scheme 28a) [81]. A lower energy wavelength irradiation was able to be used, which limited the amount of background reaction as the absorption spectra of photocatalyst and substrate were more clearly resolved. The first intermolecular process using these catalysts was the [2 + 2] photocycloaddition of 2-pyridones **186** and acetylenedicarboxylates **187** catalysed by *ent-***183** to give cyclobutenes **188** (Scheme 28b) [82]. Another intermolecular reaction was later developed, this time using catalyst **185** for the [2 + 2] photocycloaddition of quinolones **189** and electron-deficient alkenes **190** to synthesise cyclobutanes **191** (Scheme 28c) [83]. Recently, Bach et al. also employed this methodology for the intramolecular [2 + 2] cycloaddition of quinolones **192** containing either a pendant alkene or allene to obtain cyclobutanes **193** (Scheme 28d) [84].

**Scheme 29:**
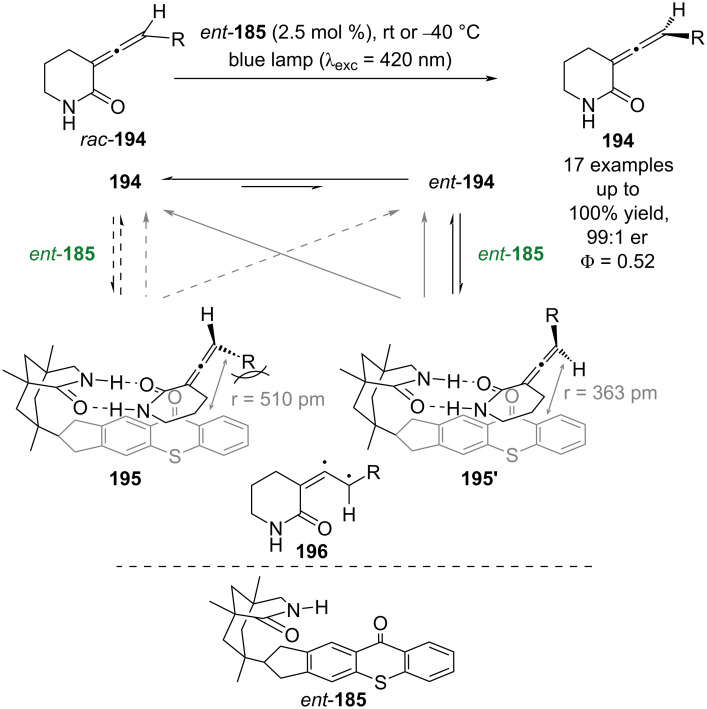
Bifunctional hydrogen bonding/photocatalysed deracemisation of allenes.

Recently, *ent*-**185** was applied by Bach to the deracemisation of allenes *rac-***194** (Scheme 29) [85]. The proposed mechanism proceeds through configurationally isomeric hydrogen bonding complexes **195** and **195’**, with subsequent photoexcitation of the thioxanthone chromophore leading to racemisation of the allene through a triplet state intermediate **196**. In **195** there is additional steric repulsion between the large R group and the thioxanthone that is not present in **195’**, which results in a smaller association constant and a larger calculated separation between the allene and the chromophore in **195** (r = 510 pm) relative to **195’** (r = 363 pm). As the efficiency of Dexter energy transfer decreases exponentially with distance, the larger gap results in lower sensitisation rates for **190**. This, in combination with the difference in association constants, leads to greater racemisation of *ent*-**194** and, therefore, a deracemisation with excellent enantioselectivities and yields (17 examples, up to 99:1 er). Interestingly, the quantum yield for the reaction was measured to be 0.52, which the authors assert is quantitative based on the complete deracemisation in the reaction. Bach et al. recently extended this reactivity further to include 5-membered rings [86].

**Scheme 30:**
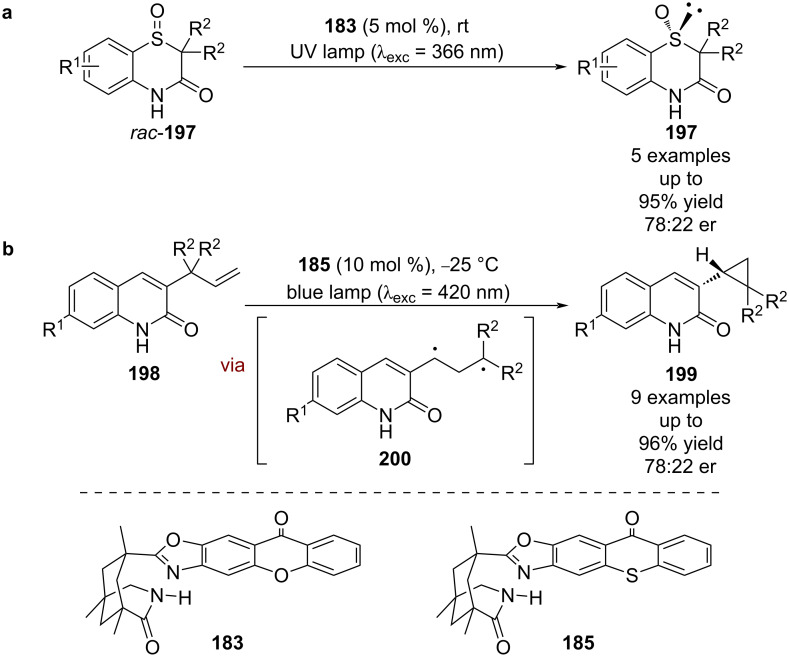
Bifunctional hydrogen bonding/photocatalysed deracemisation reactions. (a) Deracemisation of sulfoxides. (b) Photochemical rearrangement followed by photocatalysed deracemisation of the resulting cyclopropane products.

A similar mechanism is proposed for the deracemisation of sulfoxides *rac*-**197**; however, the enantioselectivities are lower (5 examples, up to 78:22 er), which the authors attribute to the catalyst differentiating between the sterics of an oxygen atom and a lone pair of electrons (Scheme 30a) [87]. Bach et al. also found that alkenes **198** can undergo a light-induced rearrangement to cyclopropane **199** in the presence of **185** (Scheme 30b) [88]. They discovered that **199** is configurationally unstable under the reaction conditions and propose that a similar deracemisation mechanism is responsible for the enantioselectivity via triplet state intermediate **200**, rather than proceeding via an enantioselective rearrangement.

**Scheme 31:**
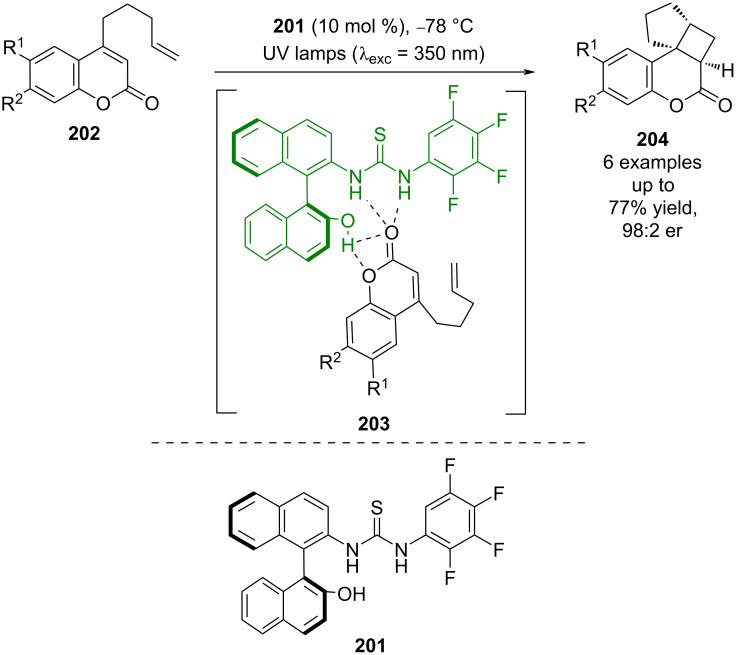
Bifunctional hydrogen bonding/photocatalysed intramolecular [2 + 2] photocycloaddition of coumarins.

Bifunctional hydrogen bonding photocatalysts have been developed by other groups as well. Sivaguru developed an atropisomeric thiourea-based catalyst **201** and used it for the intramolecular photocycloaddition of coumarins **202** (Scheme 31) [89]. The proposed mechanism for this reaction is similar to that proposed by Bach and Krische, proceeding via a key hydrogen bonding complex **203**. Interestingly, this catalyst allowed for reactivity with lactones, whereas Bach’s catalysts are limited to lactams.

**Scheme 32:**
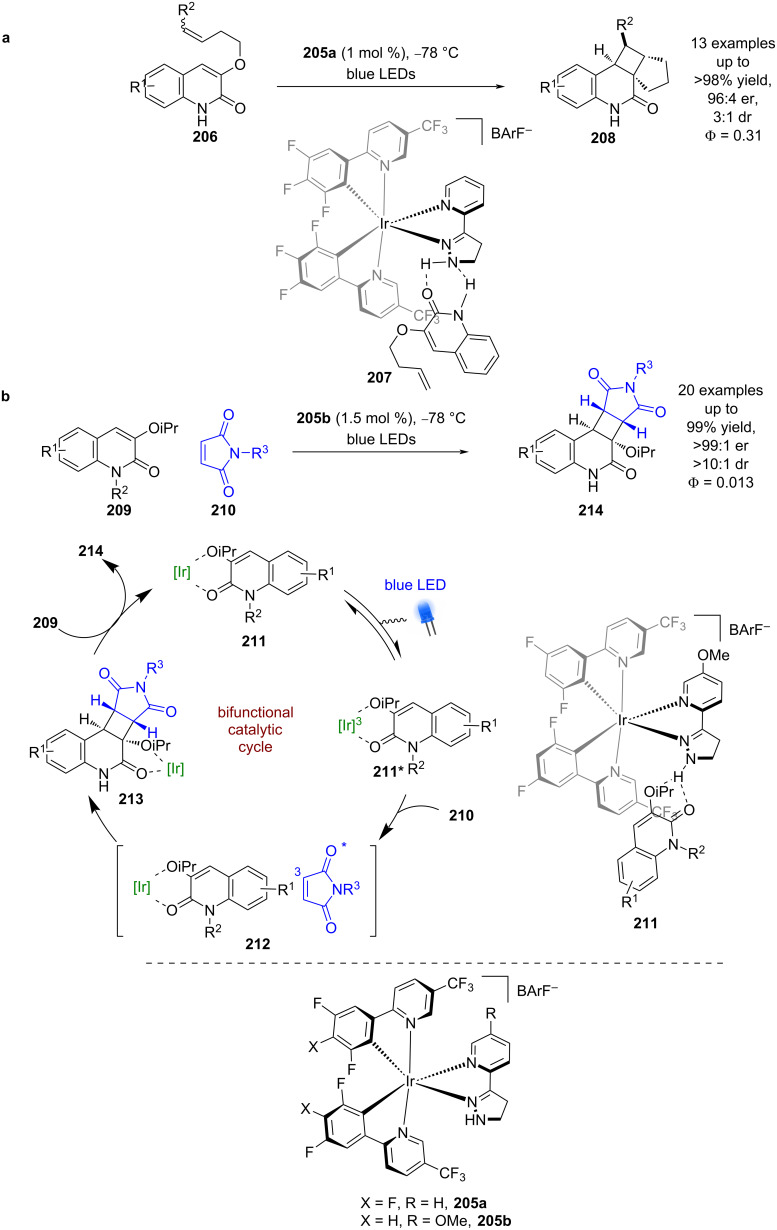
Bifunctional hydrogen bonding/photocatalysed [2 + 2] photocycloadditions of quinolones. (a) Intramolecular cycloaddition of quinolones. (b) Intermolecular cycloaddition of quinolones and maleimides.

Yoon et al. developed an iridium-based bifunctional hydrogen bonding photocatalyst **205**. To demonstrate the effectiveness of this photocatalyst system, they tested the intramolecular photocycloaddition of quinolones **206** (Scheme 32a) [90]. The proposed mechanism again proceeds via a triplet sensitisation of the substrate within a hydrogen bonding complex **207** that provides the desired products **208** in excellent yields and good enantioselectivities (13 examples, up to 96:4 er) and a quantum yield of 0.31 [91]. As with Bach’s catalysts, the scope is limited to lactams. Yoon et al. then applied a similar catalyst to an intermolecular photocycloaddition between quinolone **209** and maleimide **210** (Scheme 32b) [92]. After extensive mechanistic investigations, the proposed mechanism for this reaction is markedly different to the intramolecular example in Scheme 32a. Firstly, the hydrogen-bonded complex **211** that forms is proposed to involve the alkoxy group rather than the N–H bond that is usually invoked. Then, upon photoexcitation, Dexter energy transfer to the maleimide is dominant to generate ^3^maleimide rather than energy transfer to the quinolone. While some maleimide dimerisation is observed, which supports the presence of triplet maleimide, a rapid cycloaddition occurs with the now ground-state hydrogen-bonded quinolone–iridium complex within the solvent cage pair **212** to give complex **213**. Subsequent displacement by another substrate molecule releases the desired products **214** in excellent yields and enantioselectivities (20 examples, up to >99:1 er), with a quantum yield of 0.013.

**Scheme 33:**
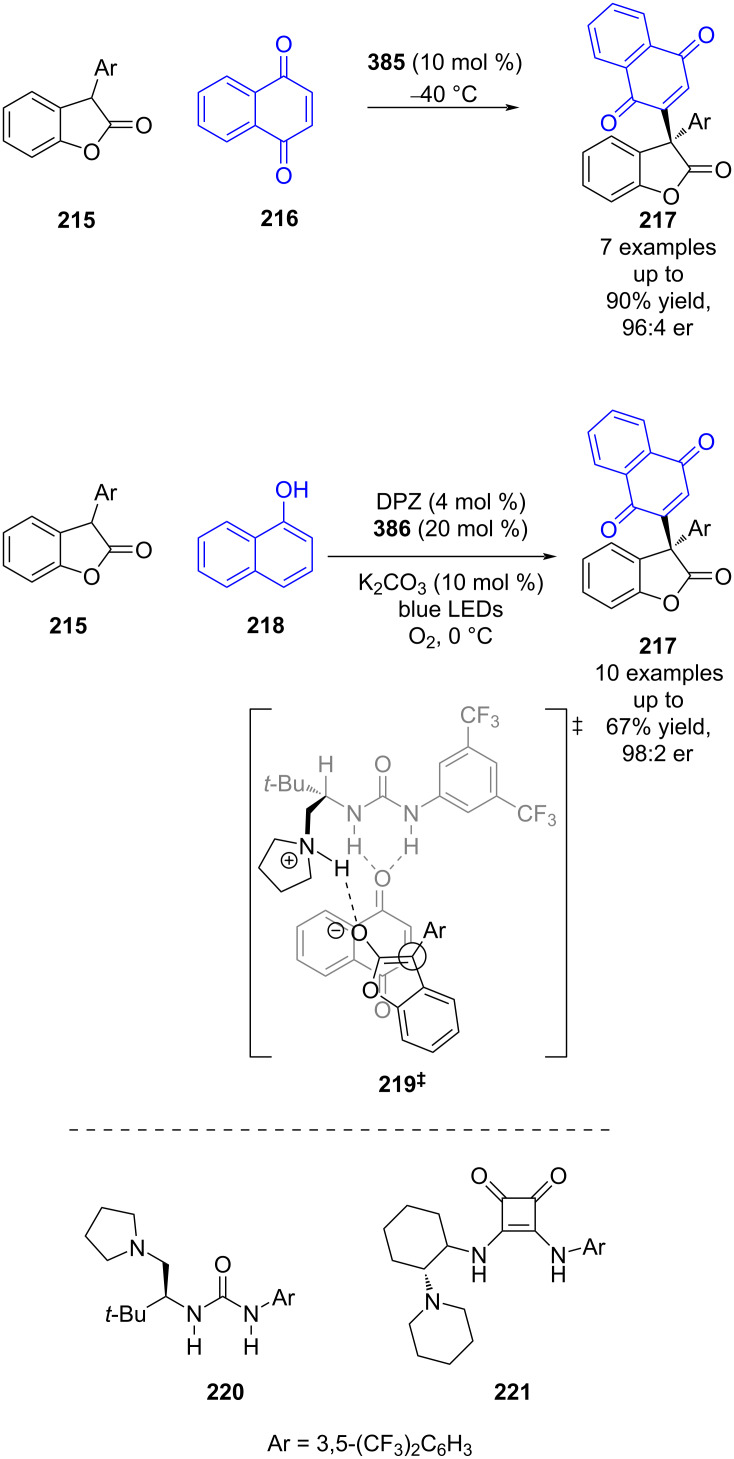
Hydrogen bonding/photocatalysed formal arylation of benzofuranones.

While there has been significant progress using bifunctional catalysts, dual catalytic systems can offer other modes of reactivity. For instance, Jiang et al. developed a urea-catalysed formal arylation of benzofuranones **215**, using naphthoquinones **216** to afford enantioenriched benzofuranones **217** (Scheme 33) [93]. They then expanded the scope of the naphthoquinone by coupling this reaction with a photocatalysed oxidation of naphthols **218** to generate **216** in situ. While no detailed mechanism has been proposed, based on prior work of Hawkins et al. [94] it is suggested that this reaction uses ^1^O_2_ for the oxidation, and proceeds through transition state **219^‡^** for the hydrogen-bonded catalysed nucleophilic addition step.

**Scheme 34:**
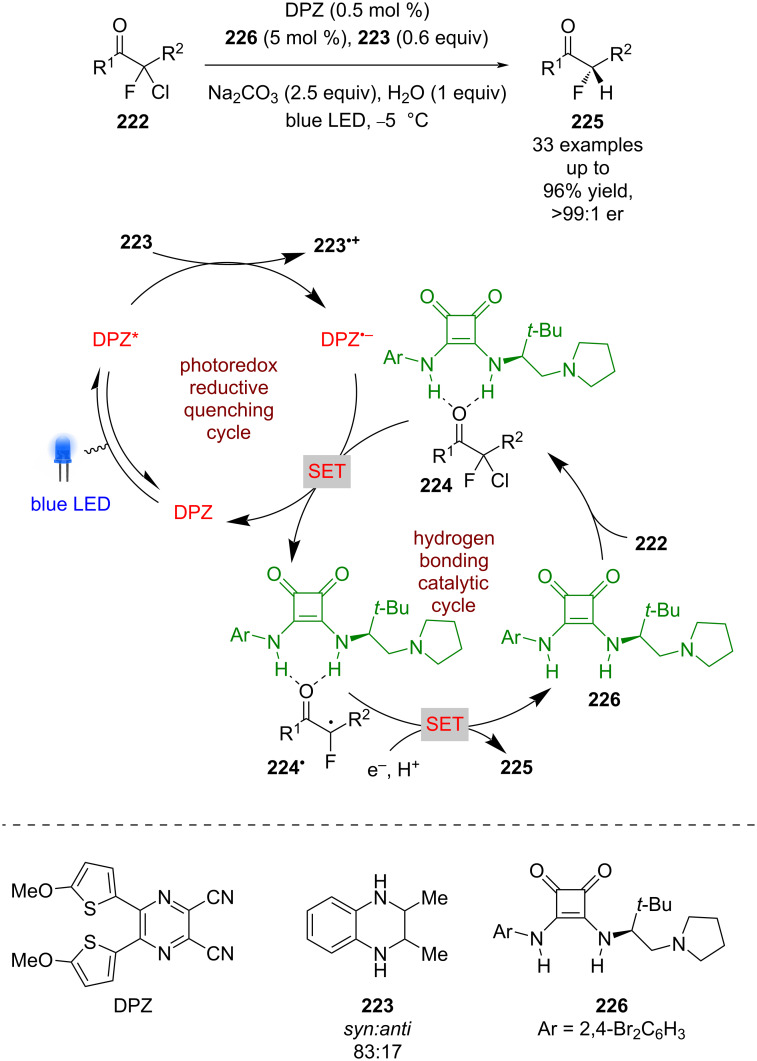
Hydrogen bonding/photoredox-catalysed dehalogenative protonation of α,α-chlorofluoro ketones.

Jiang et al. recently applied a similar dual catalytic system to the dehalogenative protonation of α,α-chlorofluoro ketones **222** (Scheme 34) [95]. A reductive quenching cycle is proposed using tetrahydroquinoxaline **223** as a sacrificial reductant to generate DPZ^•−^, which then reduces the organocatalyst-bound substrate **224** to give α-carbonyl radical **224^•^**. Further reduction to the corresponding enolate and enantioselective protonation furnished the desired enantioenriched α-fluoroketones **225** in excellent yields and enantioselectivities (33 examples, up to >99:1 er).

**Scheme 35:**
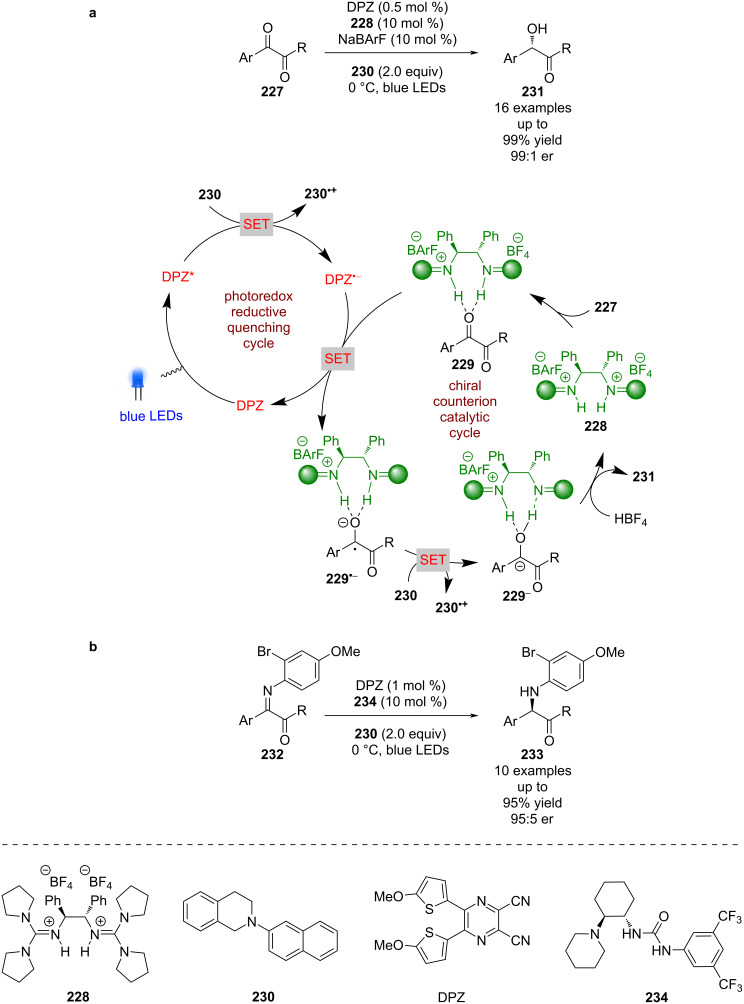
Hydrogen bonding/photoredox-catalysed reductions. (a) Reduction of 1,2-diketones. (b) Reduction of imines.

Jiang et al. used a related system for the enantioselective reduction of 1,2-diketones **227** (Scheme 35a) [96]. In this case the catalyst **228** is proposed to form hydrogen-bonded complex **229**. THIQ **230** is used as a sacrificial reductant to generate DPZ^•−^, which reduces **229** to give radical anion intermediate **229^•−^**. Further reduction by **230** results in carbanion **229^−^**, which is protonated enantioselectively to give the desired α-hydroxy ketones **231** in excellent yields and enantioselectivities (16 examples, up to 99:1 er). Within the same publication Jiang et al. expands this reactivity to activated imines **232**, using slightly modified conditions to obtain α-amino ketones **233** in excellent yields and good enantioselectivities (10 examples, up to 95:5 er) (Scheme 35b). It is also worth noting that this reaction works in the absence of DPZ (albeit with lower enantioselectivities for some examples), which is proposed to be due to the formation of an EDA complex between **230** and **229**.

**Scheme 36:**
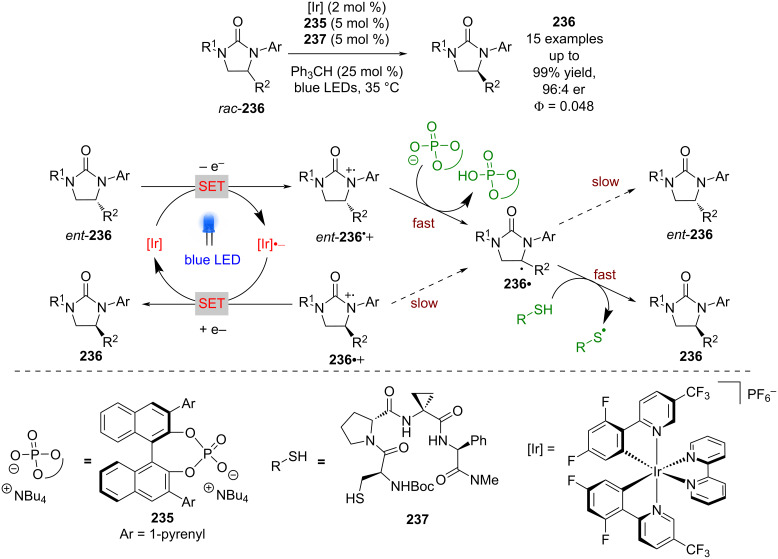
Hydrogen bonding/HAT/photocatalysed deracemisation of cyclic ureas.

CPAs were shown to be excellent partners for dual photoredox catalysis in the section "Brønsted acid catalysis", yet interestingly their conjugate bases can also be used as efficient hydrogen bonding catalysts. Knowles et al. showed that a tricatalytic system using chiral phosphate **235** can mediate the deracemisation of cyclic urea *rac*-**236** (Scheme 36) [97]. The proposed mechanism involves a reversible reductive quenching step to give two enantiomeric radical cations **236^•+^** and *ent*-**236^•+^**. The acidified adjacent proton can then be abstracted by a base to give the racemic radical **236^•^**, which then undergoes HAT with a thiol HAT catalyst to complete the racemisation. If an appropriate chiral base is used, then *ent*-**236^•+^** can be deprotonated, and therefore racemised faster than **236^•+^**, leading to a build-up of one enantiomer. This process initially achieved an er of 86:14, but with the inclusion of a complementary chiral HAT catalyst **237** and a radical scavenger (Ph_3_CH), the er could be improved to 96:4.

**Scheme 37:**
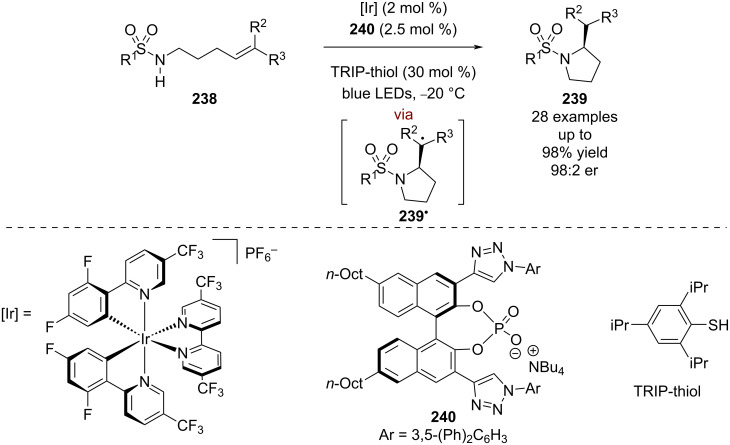
Hydrogen bonding/HAT/photoredox-catalysed synthesis of cyclic sulfonamides.

Recently, Knowles et al. used a similar tricatalytic system for the enantioselective cyclisation of sulfonamides **238** (Scheme 37) [98]. In this case, the proposed mechanism involves a PCET step to give an *N*-centred radical that then cyclises enantioselectively to give the alkyl radical intermediate **239^•^**, which abstracts a hydrogen atom from TRIP-thiol to produce enantioenriched cyclic sulfonamides **239** in excellent yields and enantioselectivities (28 examples, up to 98:2 er).

**Scheme 38:**
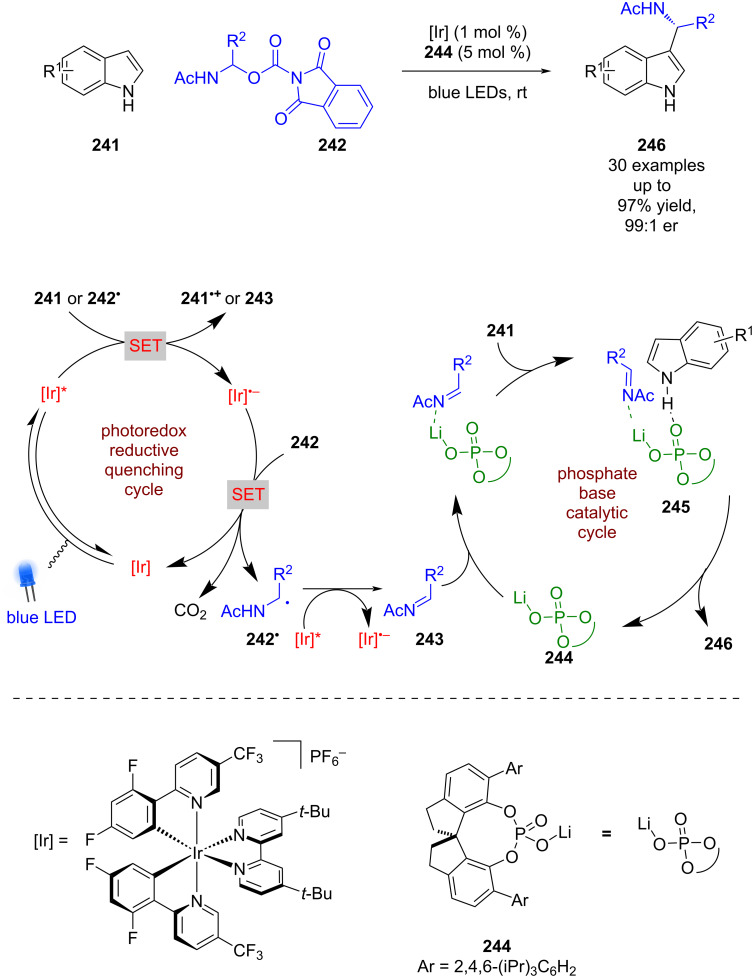
Hydrogen bonding/photoredox-catalysed reaction between imines and indoles.

Chiral phosphates have also been used to catalyse the enantioselective nucleophilic addition of indoles **241** to imines, which are photocatalytically generated in situ from redox-active esters **242** (Scheme 38) [99]. The mechanism advanced by Wang et al. proposes that **241** acts as a sacrificial reductant to generate the reduced photocatalyst, which can then reduce **242** in a second SET step to give α-amino radical **242^•^** after decarboxylation. The excited photocatalyst is reductively quenched by **242^•^** to give the imine intermediate **243**. Indoles **241** and **243** are then brought together by the chiral phosphate catalyst **244** and the lithium counterion in a hydrogen-bonded complex **245** to give the desired enantioenriched products **246** in excellent yields and enantioselectivities (30 examples, up to 99:1 er).

#### Ion pair

**Scheme 39:**
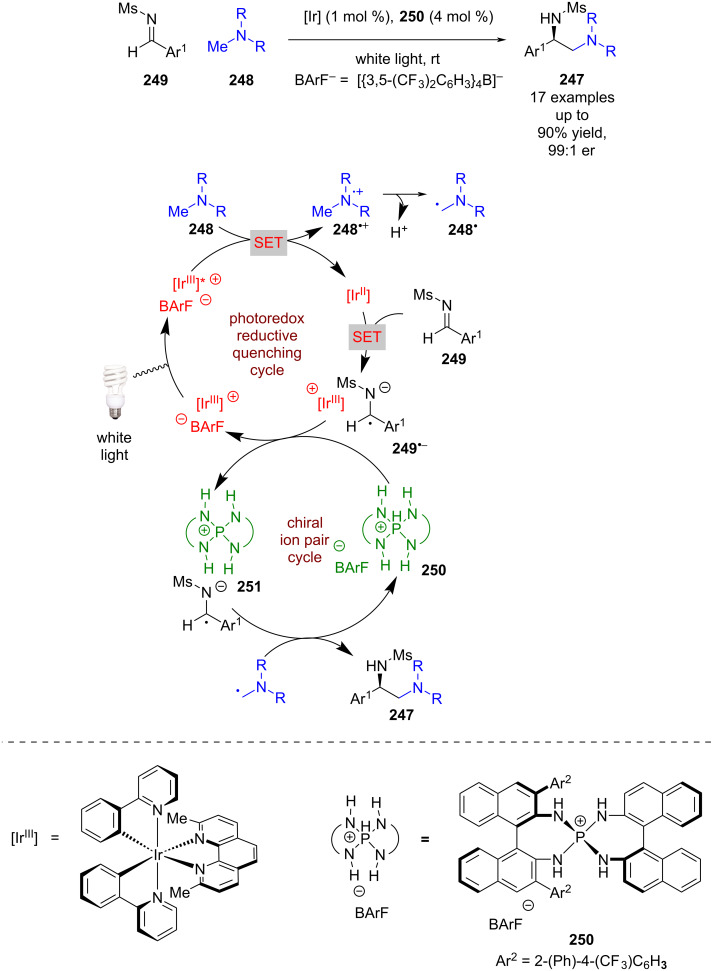
Chiral cation/photoredox-catalysed radical coupling of two α-amino radicals.

Ion pair catalysis has interesting potential in combination with photoredox catalysis considering that the catalytic intermediates are often radical cations or anions. Despite this, there are relatively few examples of this dual catalytic mode. Ooi et al. reported an enantioselective synthesis of 1,2 diamines **247** from tertiary amines **248** and aldimines **249** (Scheme 39) [100]. The proposed mechanism involves a reductive quenching pathway with **248** to produce radical cations **248**^•+^, which following deprotonation and a [1,2]-radical shift generates α-amino radicals **248^•^**. Simultaneously, **249** is reduced by the reduced photocatalyst to give radical anion **249^•−^** that can then undergo a cation exchange with chiral cationic acid **250** and form a chiral ion pair **251**. The two radical species then couple enantioselectively within the chiral environment, producing **247** in excellent yields and enantioselectivities (17 examples, up to 99:1 er). The authors noted that they cannot rule out the alternative mechanism whereby a radical addition of **248^•^** to an acid coordinated **249** is followed by a single electron reduction of the resulting *N*-centred radical.

**Scheme 40:**
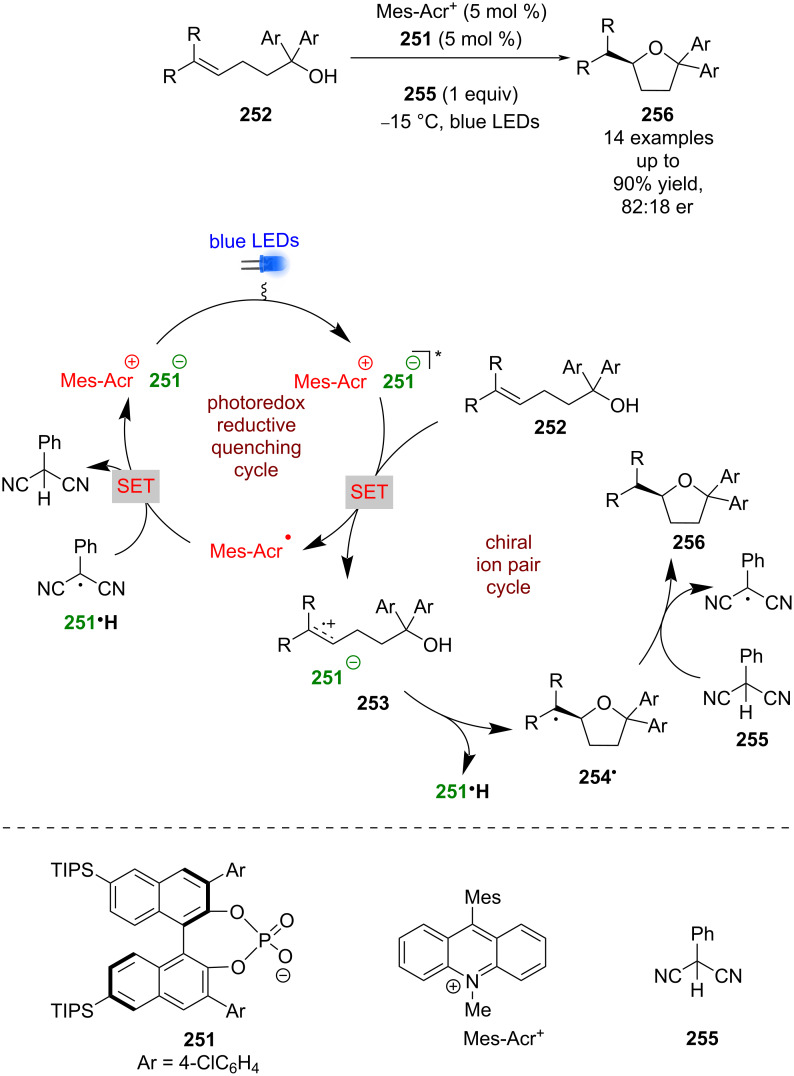
Chiral phosphate/photoredox-catalysed hydroetherfication of alkenols.

The previous example used a chiral cation to induce enantioselectivity, while Luo et al. used a chiral phosphate base **251** as a counterion to Mes-Acr^+^ (Scheme 40) [101]. With this combination, they successfully developed an enantioselective variant of Nicewicz’s hydroetherification reaction of alkenols **252** [102]. The proposed mechanism proceeds through a reductive quenching cycle that generates chiral ion pair **253**. Subsequent enantioselective cyclisation gives tertiary alkyl radical **254^•^**, which can abstract a hydrogen atom from 2-phenylmalonitrile (**255**) to afford the desired enantioenriched tetrahydrofuran **256** in excellent yields and moderate enantioselectivities (14 examples, up to 82:18 er).

**Scheme 41:**
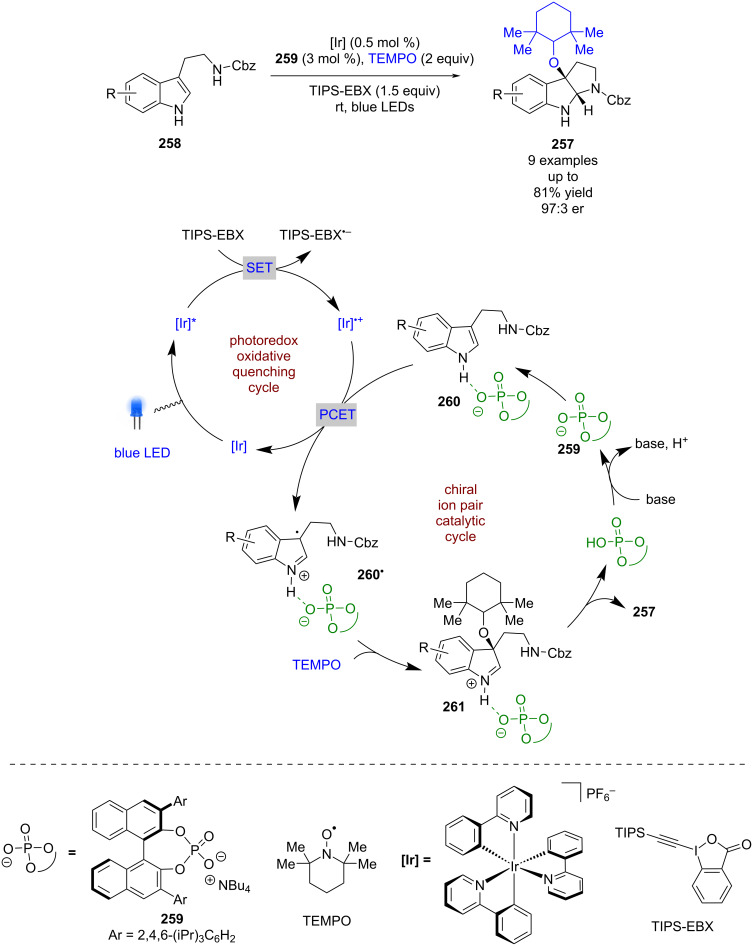
Chiral phosphate/photoredox-catalysed synthesis of pyrroloindolines.

Knowles et al. synthesised enantioenriched pyrroloindolines **257** from indoles **258** and TEMPO using an iridium-based photocatalyst and a similar chiral phosphate base **259** to that employed by Luo et al. (Scheme 41) [103]. The proposed mechanism implicates an oxidative quenching cycle using a sacrificial oxidant (TIPS-EBX), followed by a PCET step with hydrogen-bonded complex **260** to give chiral ion pair **260^•^**, which completes the photocatalytic cycle. Subsequent enantioselective radical coupling with TEMPO gives catalyst-bound iminium ion **261**, which then cyclises with the nearby amine to produce the desired product **257** in good yields and enantioselectivities (9 examples, up to 97:3 er).

**Scheme 42:**
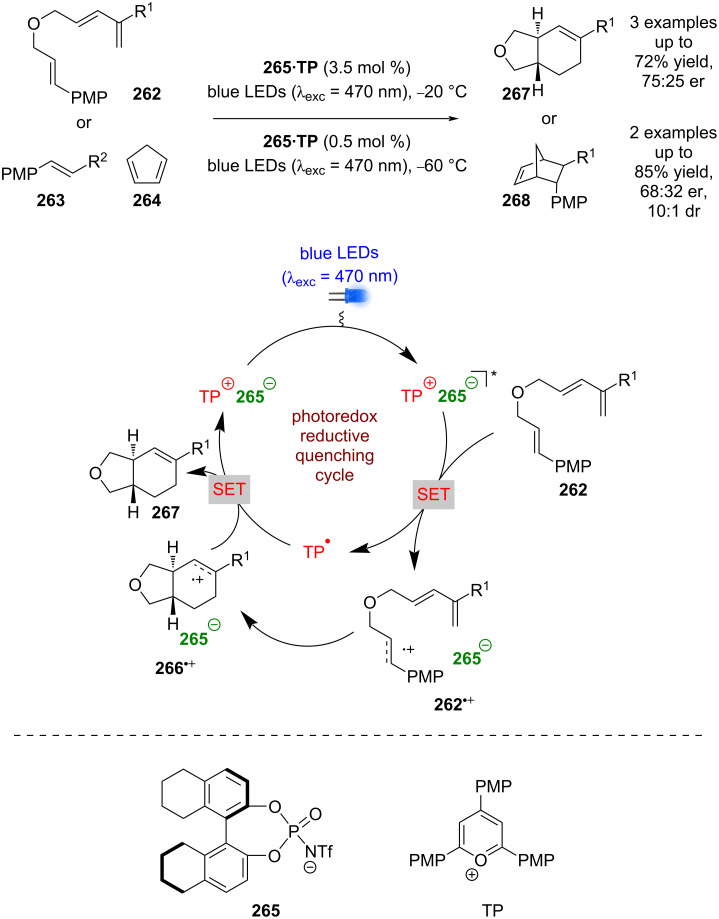
Chiral anion/photoredox-catalysed radical cation Diels–Alder reaction.

Nicewicz et al. developed an enantioselective radical cation Diels–Alder reaction in both an intramolecular fashion, using alkenes **262**, and an intermolecular fashion, using alkene **263** and cyclopentadiene **264** (Scheme 42) [104]. Using a similar strategy to Luo et al., Nicewicz et al. uses a preformed chiral photocatalyst composed of a cationic triaryl pyrillium, TP, twinned with a chiral counterion **265**. With an electron-rich alkene, the reaction is proposed to proceed via a reductive quenching cycle to generate chiral ion pair **262^•+^** and TP^•^. Subsequent enantioselective cycloaddition with a diene results in ion pair **266^•+^**, which is then reduced by TP^•^ to complete the catalytic cycle and affords the desired products **267** or **268** in moderate yields and enantioselectivities (3 examples, up to 75:25 er for **267** and 2 examples, up to 68:32 er for **268**).

#### Lewis acid catalysis

**Scheme 43:**
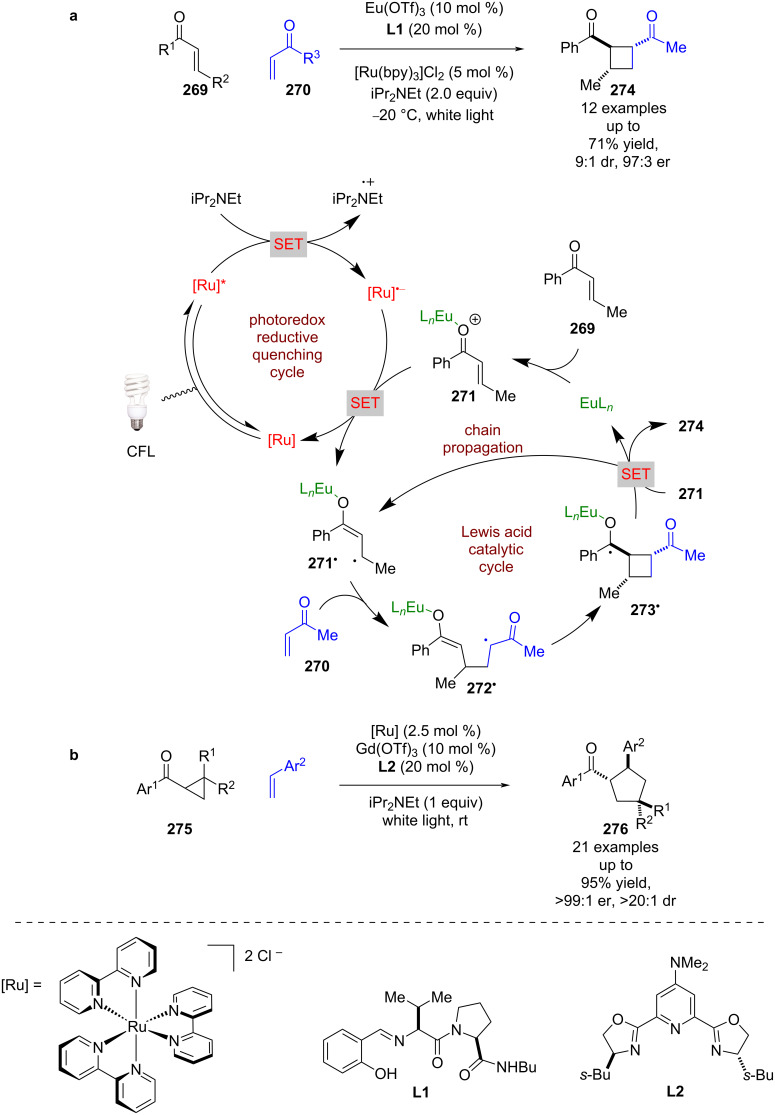
Lewis acid/photoredox-catalysed cycloadditions of carbonyls. (a) Formal [2 + 2] cycloaddition of enones using europium Lewis acid. (b) Formal [3 + 2] cycloaddition of cyclopropyl ketones with styrenes using a gadolinium Lewis acid catalyst.

Lewis acids have been known for decades to activate carbonyl compounds through the formation of coordination complexes that increases carbonyl electrophilicity [105]. The use of chiral Lewis acids can induce asymmetry [106]. Yoon et al. applied this well-known form of catalysis to a photocatalytic system using enones **269** and **270** (Scheme 43a) [107]. Mechanistic studies of a closely related achiral reaction [20], showed this reaction likely operates via a radical chain mechanism. Initiation begins with the reductive quenching of the photocatalyst using iPr_2_NEt as a sacrificial reductant to give [Ru]^•−^, which then reduces the Lewis acid-coordinated enone **271** to give alkyl radical **271^•^**. In the presence of a second enone **270** and chiral ligands **L1**, an initial RCA occurs to generate α-carbonyl radical **272^•^**, followed by cyclisation with the enolate to give ketyl radical **273^•^**. Radical **273^•^** can then reduce another molecule of **271** to propagate the chain reaction and generate the desired formal photocycloaddition products **274** in good yields and enantioselectivities (12 examples, up to 97:3 er). Yoon et al. later expanded the scope of this reaction to cyclopropyl ketones **275** for the synthesis of formal [3 + 2] cycloaddition products **276** in excellent yields and enantioselectivities (21 examples, up to >99:1 er), this time using a gadolinium catalyst and chiral ligand **L2** (Scheme 43b) [108].

**Scheme 44:**
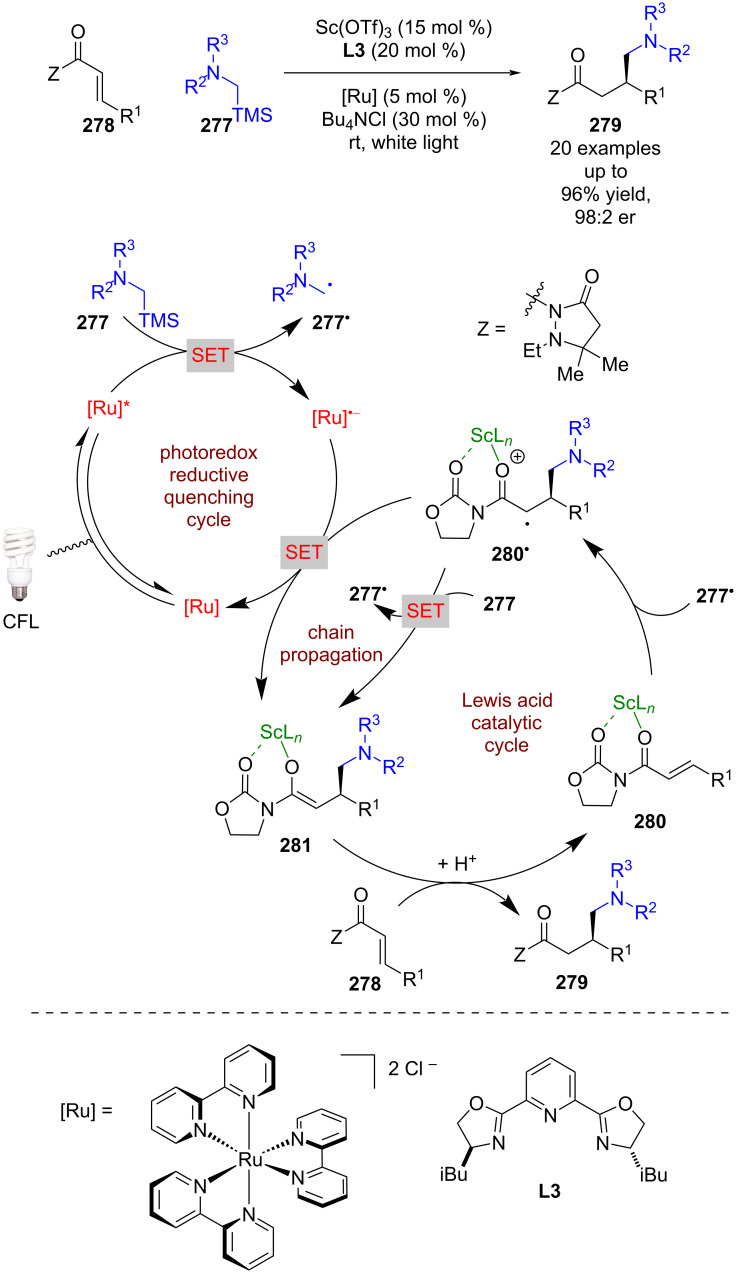
Lewis acid/photoredox-catalysed RCA reaction using a scandium Lewis acid between α-amino radicals and α,β-unsaturated amides containing an oxazolidinone auxiliary.

By altering the radical precursor to α-silyl amines **277** and using α,β-unsaturated amides **278**, Yoon et al. found that the reactions could be stopped at the RCA step to give enantioenriched 1,4-addition products **279** using a scandium catalyst and chiral ligand **L3** (Scheme 44a) [109]. The putative mechanism proceeds via a reductive quenching cycle to give nucleophilic α-amino radicals **277^•^**, which can add to the β-position of Lewis acid complex **280** to give the α-carbonyl radical **280^•^**. Instead of a cyclisation, this radical is then reduced by the reduced photocatalyst to give the corresponding enolate **281**, which is then protonated to produce **279**. Alternatively, **280^•^** could be reduced by another molecule of **277**, propagating a radical chain process, as was determined to be the case in a previously developed achiral reaction variant [110]. To improve complexation to the Lewis acid in RCA reactions, an auxiliary (Z) is required; this auxiliary can be easily removed and recovered.

**Scheme 45:**
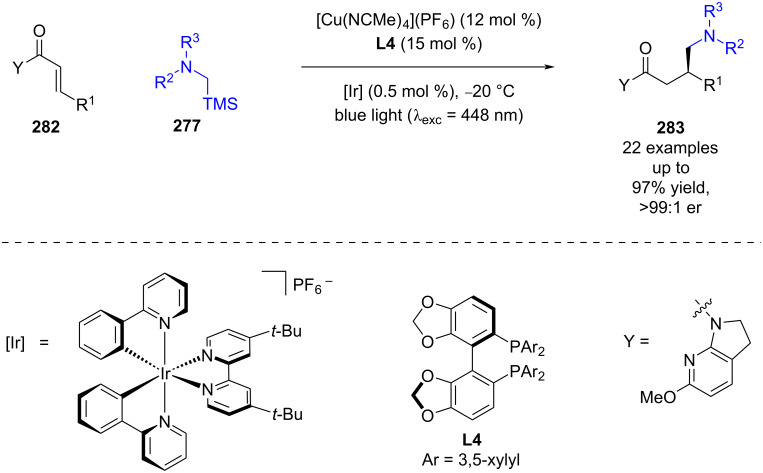
Lewis acid/photoredox-catalysed RCA reaction using a copper Lewis acid between α-amino radicals and α,β-unsaturated amides containing an azaindoline auxiliary.

Shibasaki and Kumagai very recently developed a similar reaction using a copper Lewis acid catalyst and a different auxiliary containing amide **282** with α-silyl amines **277** to synthesise the corresponding RCA products **283** in excellent yields and enantioselectivities (22 examples, up to >99:1 er) (Scheme 45) [111].

**Scheme 46:**
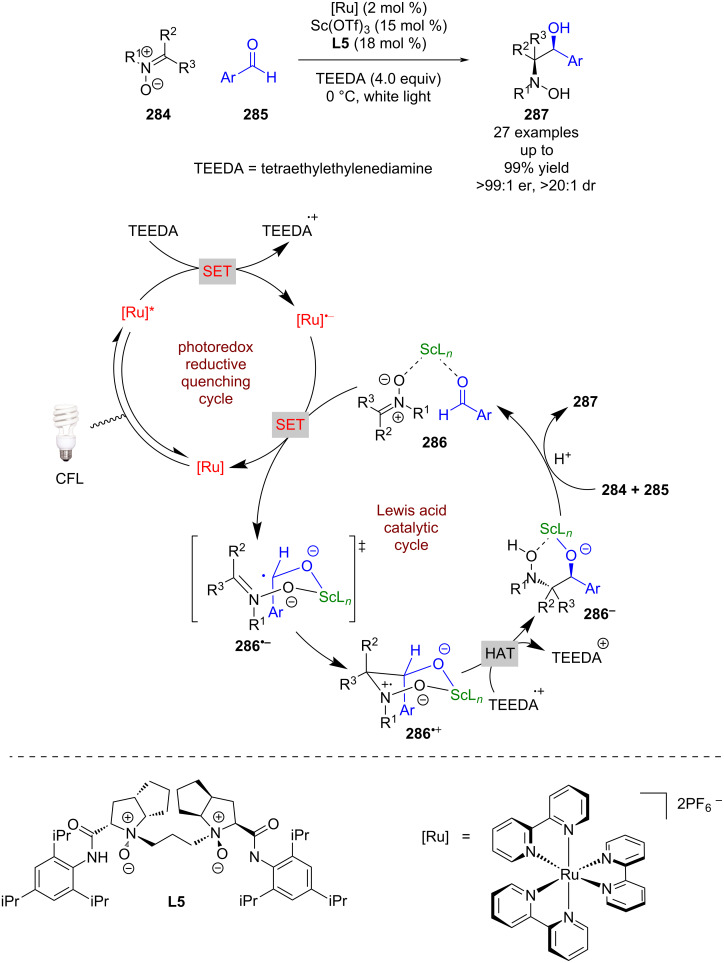
Lewis acid/photoredox-catalysed synthesis of 1,2-amino alcohols from aldehydes and nitrones using a scandium Lewis acid.

Huang et al. applied a similar catalytic system to that reported by Yoon et al. to a different radical addition reaction between nitrones **284** and aldehydes **285** (Scheme 46) [112]. The proposed mechanism involves a reductive quenching cycle using TEEDA as a sacrificial reductant to generate [Ru]^•−^. Simultaneously the chiral Lewis acid catalyst forms complex **286** with both starting materials. [Ru]^•−^ then reduces the activated aldehyde to give ketyl radical anion **286^•−^**, which adds to the nitrone via a proposed 6-membered transition state to afford radical cation **286^•+^**. Subsequent hydrogen atom abstraction from TEEDA^•+^ generates complex **286^−^**. Protonation and displacement by other substrate molecules releases the desired 1,2-amino alcohol products **287** in excellent yields and enantioselectivities (27 examples, up to >99:1 er).

**Scheme 47:**
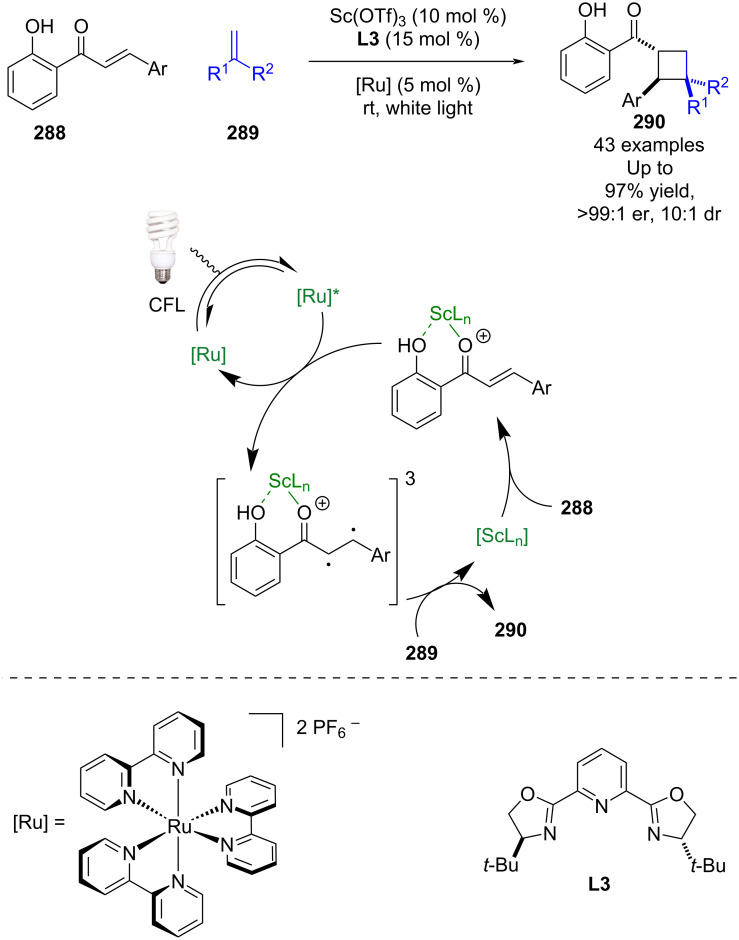
Lewis acid/photocatalysed [2 + 2] photocycloadditions of enones and alkenes.

Yoon et al. have also shown that these types of Lewis acid complexes can be used in an energy transfer process for the [2 + 2] cycloaddition of enones **288** with alkenes **289** (Scheme 47) [113–114]. The triplet energy of **288**, when complexed to the scandium Lewis acid (*E*_T_ = 1.43 eV), is significantly lower relative to the unbound substrate (*E*_T_ = 2.34 eV). They propose that Lewis acid coordination permits discrimination between bound and unbound substrate as it allows for selective triplet sensitisation of the bound substrate by the excited state ruthenium photocatalyst. The subsequent enantioselective [2 + 2] photocycloaddition gives cyclobutane products **290** in excellent yields and enantioselectivities (43 examples, up to >99:1 er).

**Scheme 48:**
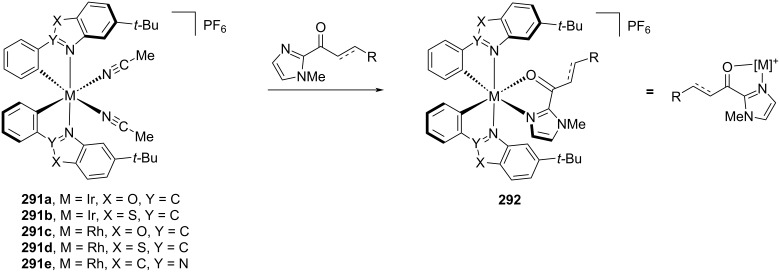
Meggers’s chiral-at-metal catalysts.

Meggers et al. has contributed significantly to the field of enantioselective photocatalysis, introducing unique transition metal Lewis acids **291a–e** that can coordinate to ketone substrates and form chiral photoactive complexes **292**, which in many cases act as the in situ generated photocatalyst (Scheme 48) [115]. They have recently developed an example using an indazole-based ligand [116] to add to their well-established benzoxazole and benzothiazole ligands. Such complexes have then been used for α-functionalisations [117], RCAs [118], and cycloaddition reactions [119]. As much of Meggers work has been summarised previously [120], here we will include only recent examples from each reaction class.

**Scheme 49:**
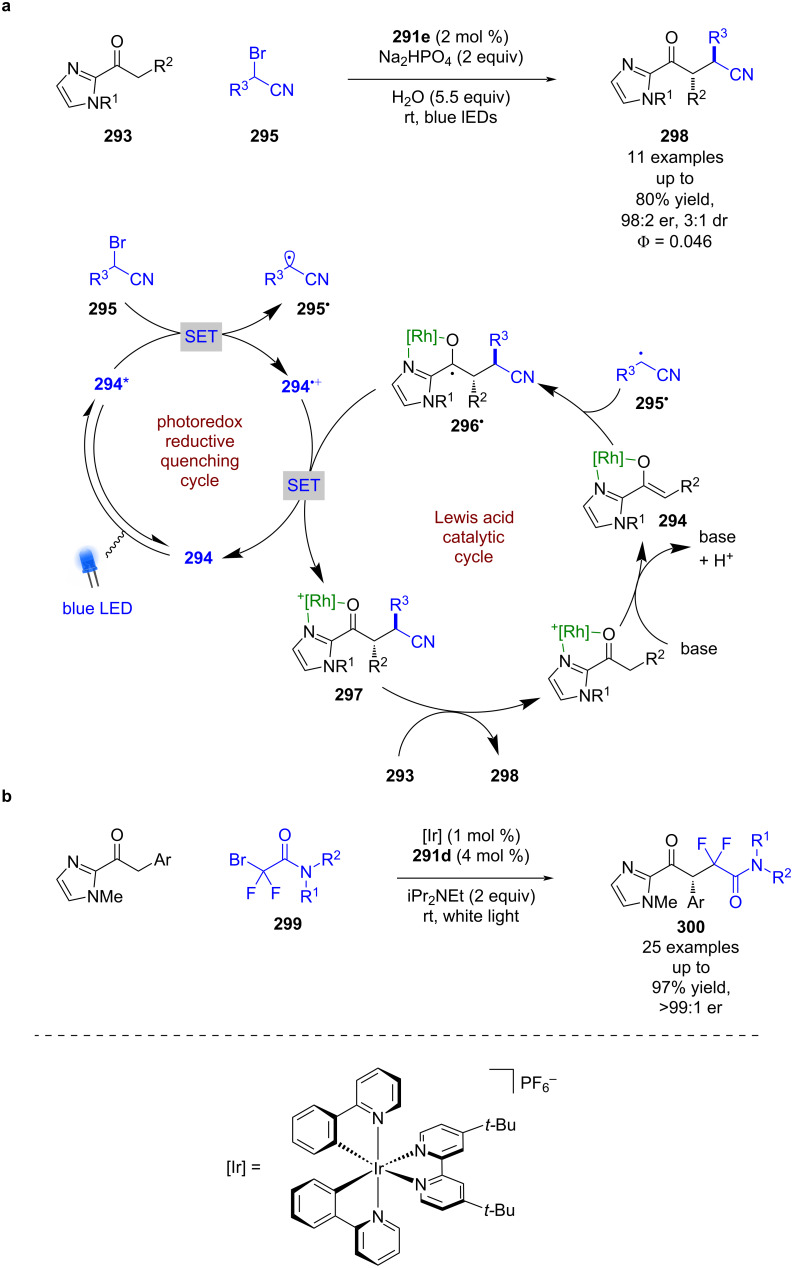
Lewis acid/photoredox-catalysed α-functionalisation of ketones with alkyl bromides bearing electron-withdrawing groups. (a) Using bifunctional Lewis acid/photoredox catalyst. (b) Using a dual catalytic system.

If an enolisable ketone **293** is used, enolate complex **294** can be formed in the presence of base (Scheme 49a) [116]. The complex in this example is then proposed to proceed via an oxidative quenching cycle with bromo nitrile **295** to form α-cyano radicals **295^•^** that then add to another molecule of **294** enantioselectively to give ketyl radical **296^•^**. These radicals are then oxidised by the oxidised photocatalyst to generate the metal-bound α-functionalised product **297**, which can be displaced by another molecule of **293** to finish the catalytic cycle and furnish the desired product **298** in good yields and excellent enantioselectivities (11 examples, up to 98:2 er). While a chain propagation mechanism is possible, the quantum yield of the reaction is <1 (Φ = 0.046), so a closed cycle is likely the dominant mechanism in this case. Another recent example of this type of reactivity was developed by Xu et al. using amides **299** in a difluoroalkylation reaction; however, this reaction did require the use of an external photocatalyst (Scheme 49b) [121].

**Scheme 50:**
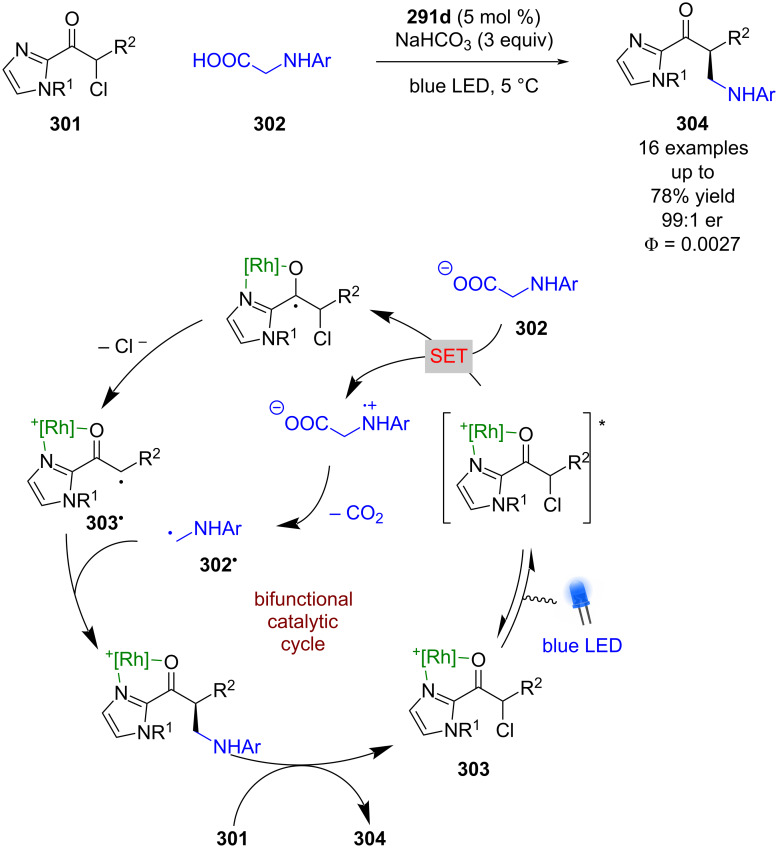
Bifunctional Lewis acid/photoredox-catalysed radical coupling reaction using α-chloroketones and α-aminocarboxylic acids.

A limitation of this strategy is that only electrophilic radicals can be added to the nucleophilic enolate complex. Recently, Meggers showed that this type of reactivity can be reversed if an α-chloro ketone **301** is used with α-aminocarboxylic acids **302** (Scheme 50) [122]. In this reaction, **301** now forms coordination complex **303**, which upon excitation is proposed to initiate a SET with **302** to form an electrophilic α-carbonyl radical **303^•^** after loss of chloride, and a nucleophilic α-amino radical **302^•^** after decarboxylation. Enantioselective radical coupling gives the metal-bound product, which can be displaced by another molecule of **301** to complete the cycle and release ketone **304** in good yields and excellent enantioselectivities (16 examples, up to 99:1 er). In this case the quantum yield was measured to be <1 (Φ = 0.0027), which suggests that a chain mechanism is unlikely.

**Scheme 51:**
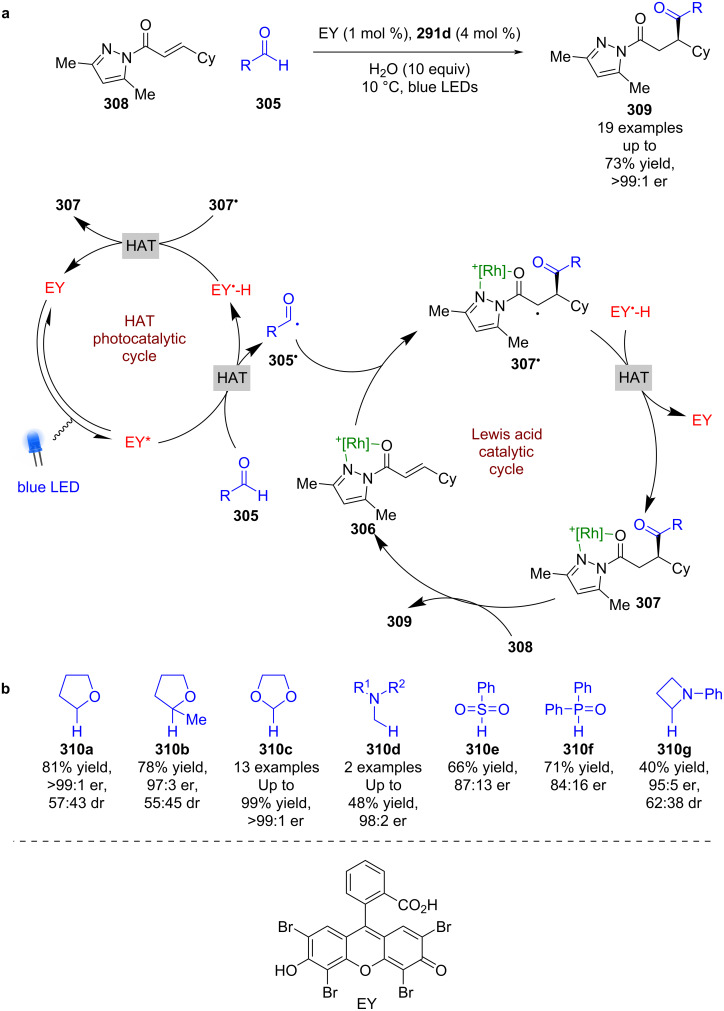
Lewis acid/photocatalysed RCA of enones. (a) Using aldehydes as acyl radical precursors. (b) Other substrates used as radical precursors.

A recent example of these catalysts being used for RCAs exploited Eosin Y as an external HAT photocatalyst to generate acyl radicals **305^•^** from aldehydes **305**, which then add to the Lewis acid complex **306** enantioselectively to form α-carbonyl radicals **307^•^** (Scheme 51a) [118]. The reverse HAT step completes the photocatalytic cycle and produces the complexed RCA product **307**, which can be displaced by another substrate molecule **308** to finish the cycle and release the desired enantioenriched products **309** in moderate yields and excellent enantioselectivities (19 examples, up to >99:1 er). In the same work, Meggers et al. also used compounds **310a**–**g** as radical precursors, with a focus on 1,3-dioxolane **310c** as a formyl radical surrogate (Scheme 51b).

**Scheme 52:**
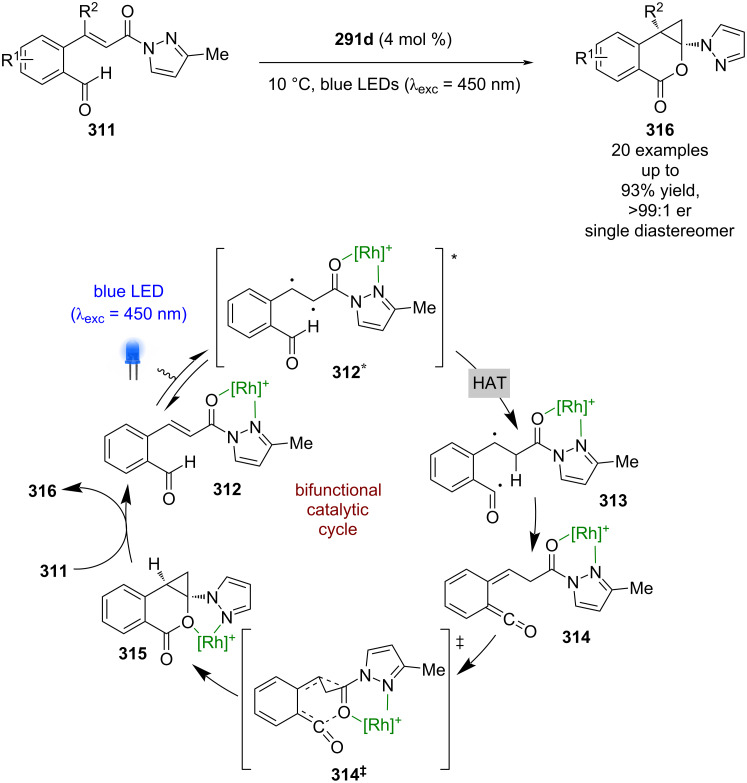
Bifunctional Lewis acid/photocatalysis for a photocycloaddition of enones.

Meggers’ complexes can also be used for photocycloadditions (Scheme 52) [123]. A recent example used enone **311** to form the corresponding metal complex **312**, which upon photoexcitation is proposed to behave like diradical 312*. HAT from the nearby aldehyde to the α-position produces acyl radical **313**, which undergoes intersystem crossing to the singlet state ketene **314** supported by DFT calculations. This intermediate then reacts through an enantioselective [4 + 2] cycloaddition via transition state **314^‡^** to give the complexed cyclisation product **315**. This product is then displaced by another substrate molecule to finish the catalytic cycle and produce the cycloaddition products **316** as a single diastereomer with excellent yields and enantioselectivities (20 examples, up to >99:1 er). In this case, the auxiliary is not easily removed, which is a limitation of this mode of catalysis.

**Scheme 53:**
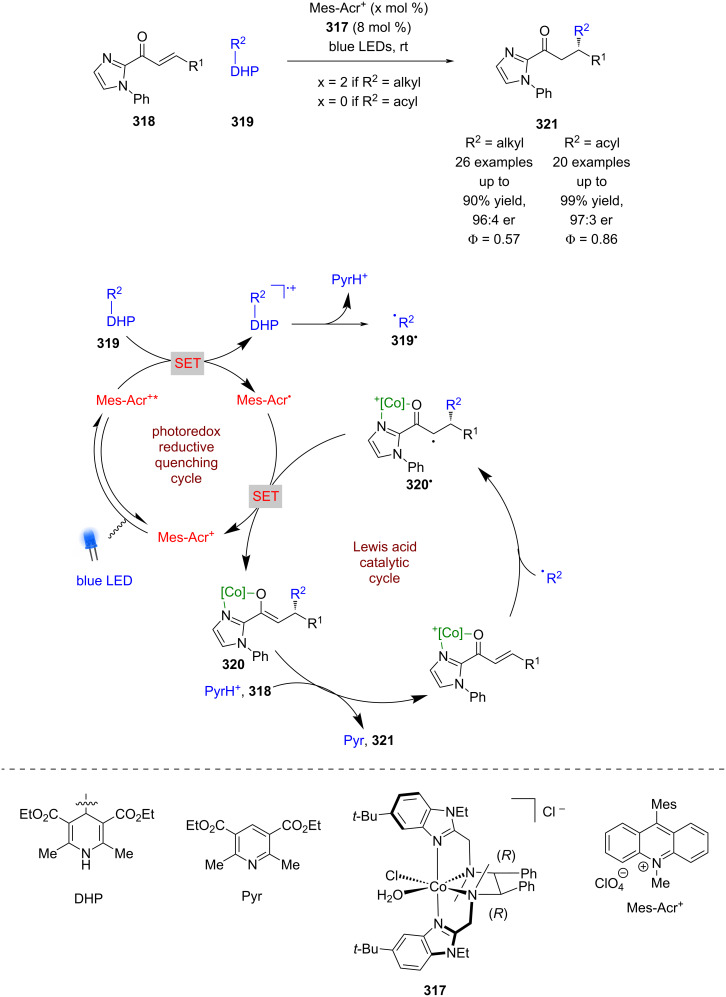
Lewis acid/photoredox-catalysed RCA reactions of enones using DHPs as radical precursors.

Recently, Xiao et al. developed a similar catalyst **317**, using a cobalt-based system where the chirality was conferred by the use of chiral ligands rather than metal-centred chirality, which is the modus operandi of Meggers’ technology (Scheme 53) [124]. They applied **317** to the enantioselective RCA reaction of enones **318** using DHPs **319** as radical precursors. If an alkyl DHP is used, then Fukuzumi’s acridinium photocatalyst Mes-Acr^+^ is required. The proposed mechanism proceeds via a reductive quenching cycle to generate alkyl radicals **319^•^** and Mes-Acr^•^. The radical **319^•^** then adds enantioselectively to the complex formed between **317** and **318** to give α-carbonyl radical **320^•^**, which is then reduced to the corresponding enolate **320** by Mes-Acr^•^ to turn over the photocatalytic cycle. Protonation of **320** and displacement by another molecule of **318** completes the Lewis acid cycle and affords the desired enantioenriched RCA products **321** in excellent yields and good enantioselectivities (26 examples, up to 96:4 er). If acyl DHPs are used, then no external photocatalyst is required, which is proposed to be due to direct excitation of the substrate as demonstrated previously by Melchiorre et al. [45]. The complexes generated do not seem able to act as photocatalysts, but the reactions do demonstrate high quantum yields for both alkyl (Φ = 0.57) and acyl (Φ = 0.86) substituents, suggesting efficient photocatalytic cycles.

**Scheme 54:**
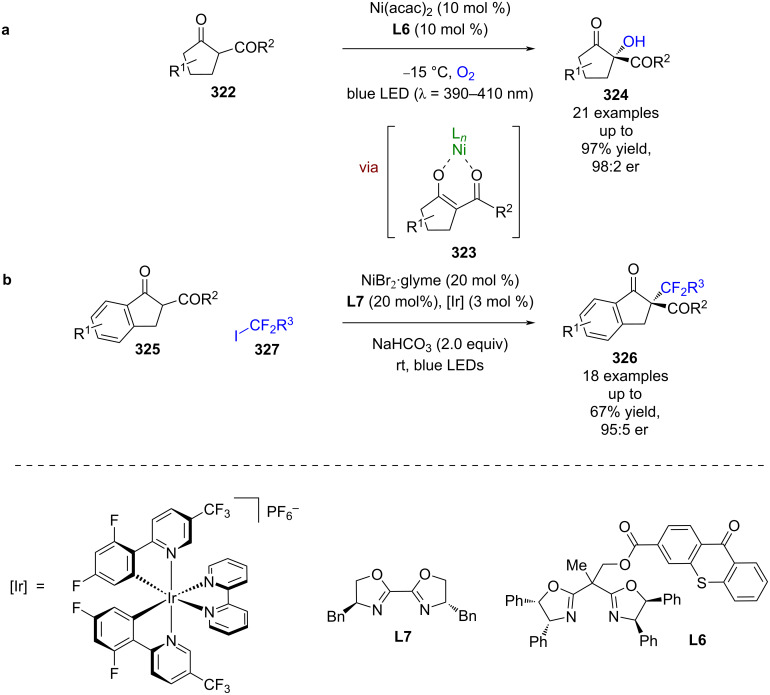
Lewis acid/photoredox-catalysed functionalisation of β-ketoesters. (a) Hydroxylation reaction catalysed by a bifunctional nickel/thioxanthone catalyst. (b) Alkylation reaction catalysed by a nickel/iridium dual catalysis system.

Other bifunctional Lewis acid/photoredox catalysts have also been reported. Xiao et al. developed a chiral ligand with a thioxanthone photoactive moiety **L6**, which when used in combination with Ni(acac)_2_ forms a bifunctional catalyst in situ (Scheme 54a) [125]. This system was then used for the oxygenation of β-ketoesters **322**, via Lewis acid complex **323** in a similar mechanism to that proposed by Gao et al. in their PTC reaction shown in Scheme 23 [73], to give α-hydroxy-β-ketoesters **324** in excellent yields and enantioselectivities (21 examples, up to 98:2 er). A similar system was then used for the alkylation of 1-indanone-derived substrates **325** although in this instance an external photocatalyst is required to obtain the desired products **326** in moderate yields and good enantioselectivities (18 examples, up to 95:5 er) (Scheme 54b) [126]. The putative mechanism is similar to that proposed by Melchiorre et al. for their PTC perfluoroalkylation process shown in Scheme 24 [77].

**Scheme 55:**
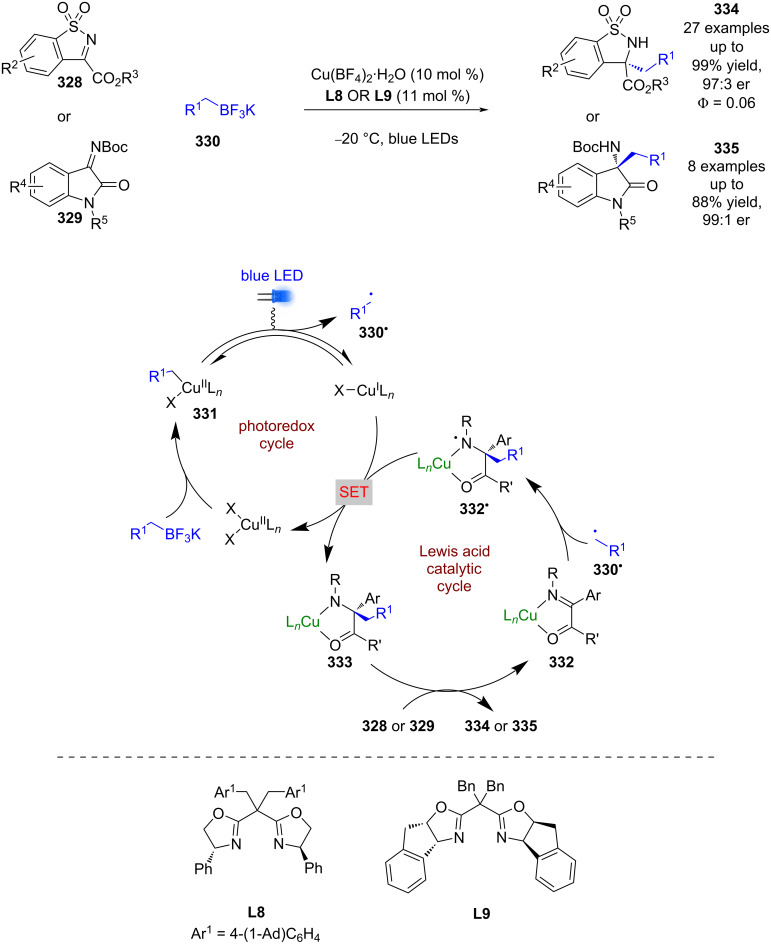
Bifunctional copper-photocatalysed alkylation of imines.

Gong et al. used a Cu(II) catalyst with chiral ligands **L8** or **L9** as a bifunctional catalyst for the enantioselective alkylation of two classes of cyclic imines **328** and **329** using trifluoroborate salts **330** (Scheme 55) [127]. Trifluoroborate salts are commonly used in photoredox catalysis as alkyl/aryl radical precursors, and generally undergo a single electron oxidation. However, Gong et al. proposes an alternative mechanism for this reaction involving a ligand exchange process to give alkyl copper(II) complex **331**, followed by a light-induced homolysis of the Cu–C bond to give alkyl radicals **330^•^** and a reduced Cu(I) species. The alkyl radical **330^•^** then adds to the copper-bound imine **332** enantioselectively to give *N*-centred radical **332^•^**, which is then reduced by Cu(I) to give the alkylation products bound to the copper catalyst **333**. Displacement by another molecule of substrate completes the cycle and releases **334** or **335** in excellent yields and enantioselectivities (27 examples, up to 97:3 er for **334** and 8 examples, up to 99:1 er for **335**). The quantum yield was measured to be <1 (Φ = 0.06), so a radical chain mechanism is unlikely to be dominant.

**Scheme 56:**
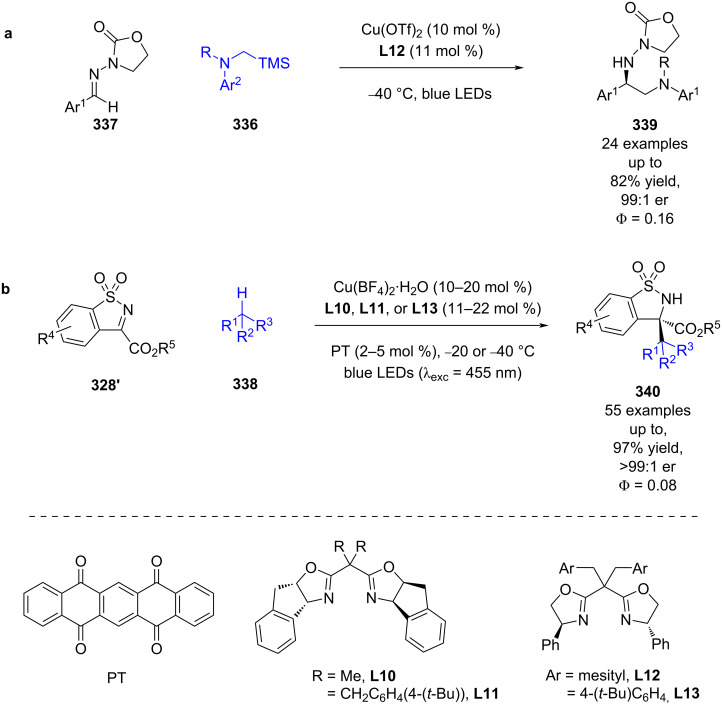
Copper/photocatalysed alkylation of imines. (a) Bifunctional copper catalysis using α-silyl amines. (b) Dual copper/HAT-catalysed C–H activation of alkanes.

Gong et al. later expanded the scope of this system to α-silyl amines **336** as radical precursors and acyclic hydrazones **337** and found that in this case an oxazolidinone auxiliary was required to aid with complexation (Scheme 56a) [128]. 5,7,12,14-Pentacenetetrone (PT) can act as both a HAT catalyst and a photocatalyst so that the alkyl radicals can be generated via C–H activation of simple alkanes **338** rather than using a redox-active group (Scheme 56b) [129]. Each reaction also had a quantum yield of <1 (Φ = 0.16 for **a** and Φ = 0.08 for **b**), so a radical chain mechanism is unlikely to be dominant.

**Scheme 57:**
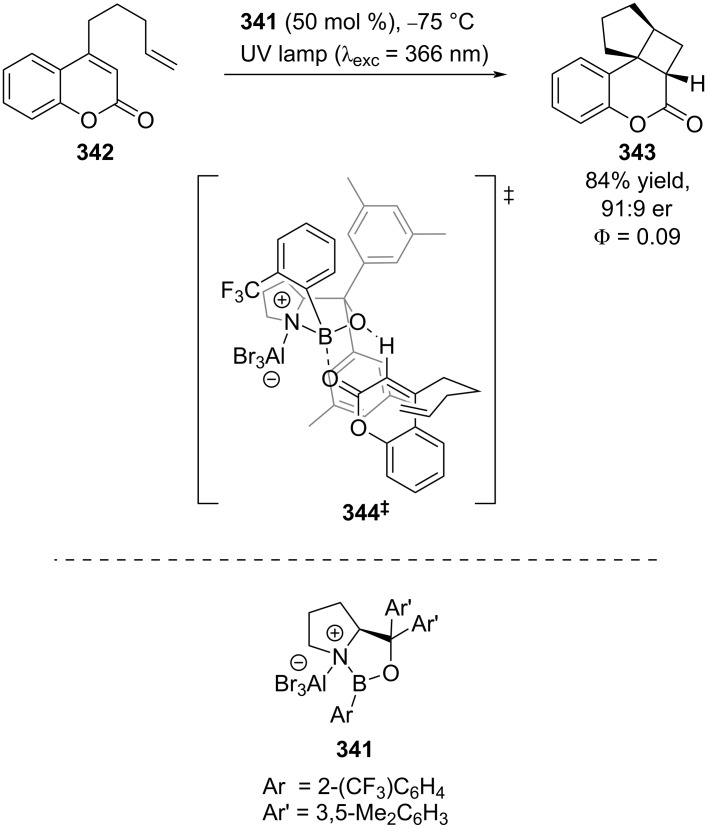
Bifunctional Lewis acid/photocatalysed intramolecular [2 + 2] photocycloaddition.

Guo and Bach took a different approach to enantioselective Lewis acid/photocatalysis, building upon work by Lewis [130] and Barancyk [131] who showed that Lewis acids could catalyse photocycloadditions. A recognised significant challenge in making these reactions enantioselective, is outcompeting the racemic background pathway. Bach et al. found that AlBr_3_-activated oxazaborolidine-based catalysts **341** could induce a bathochromic shift in the absorption spectrum of the coordination complex relative to the uncoordinated coumarin derivative **342**, allowing for selective excitation of the former (Scheme 57) [132]. With a combination of low temperatures, high catalyst loading, and tuning the excitation wavelength, the cycloaddition product **343** could be obtained with high enantioselectivity (91:9 er) via the proposed transition state **344^‡^**. Interestingly, the quantum yield of the catalysed reaction was found to be much higher (Φ = 0.09) than the uncatalysed reaction (Φ ≥ 0.002), which is proposed to be due to both increased ISC rates and increased absorption at the excitation wavelength (λ_exc_ = 366 nm) [133].

**Scheme 58:**
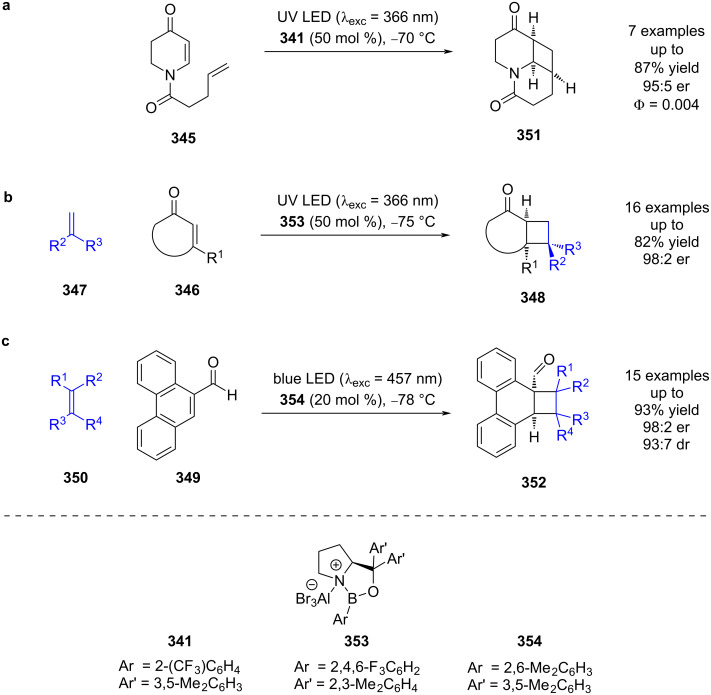
Bifunctional Lewis acid/photocatalysed [2 + 2] photocycloadditions (a) Intramolecular cycloaddition of quinolones. (b) Intermolecular cycloaddition of cyclic enones with terminal alkenes. (c) Intermolecular cycloaddition of phenanthrene-derived aldehydes and tetrasubstituted alkenes.

Bach et al. later exploited this reactivity using quinolone **345** (Scheme 58a) [134]. Interestingly, this reaction had the opposite trend for quantum yields compared to **342**, with a higher quantum yield for the background reaction (Φ ≥ 0.23) than for the Lewis acid-catalysed reaction (Φ = 0.004), which is proposed to be due to a decrease in ISC rate when coordinated to the Lewis acid. The first intermolecular example using this catalyst system used cyclic ketones **346** with alkenes **347** to synthesise bicyclic compounds **348** (Scheme 58b) [135]. A similar reaction was also later developed for the cycloaddition of phenanthrene-derived aldehydes **349** with alkenes **350**, using 457 nm excitation, which was possible due to the increased conjugation of the substrate, and a lower catalyst loading (Scheme 58c) [136].

**Scheme 59:**
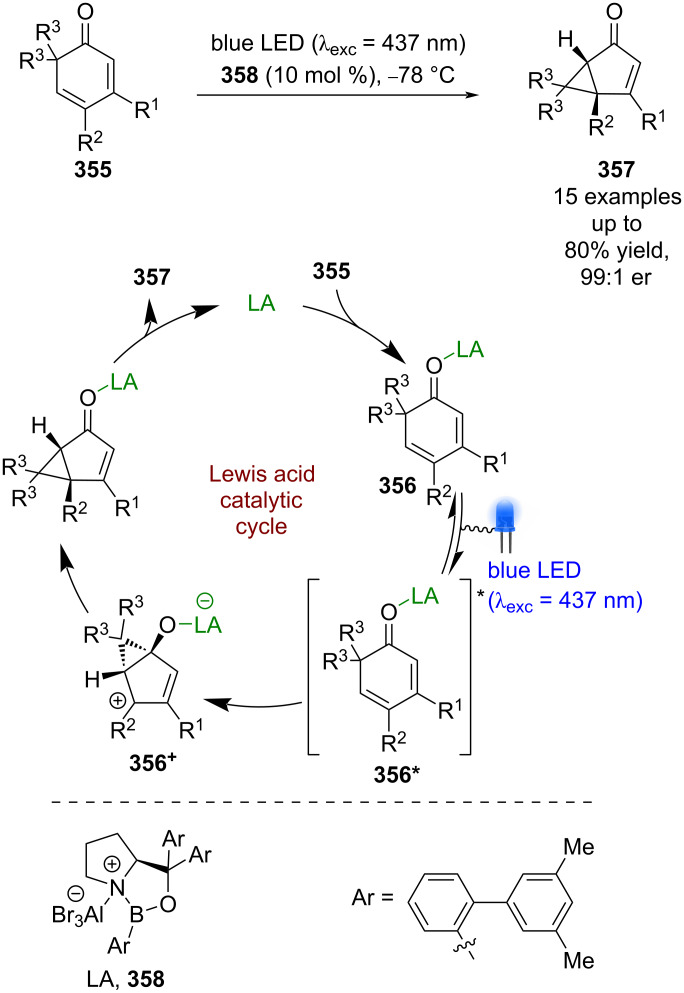
Bifunctional Lewis acid/photocatalysed rearrangement of 2,4-dieneones.

Recently, Bach et al. showed that these catalysts can also be used for photochemical rearrangements using 2,4-dienones **355** (Scheme 59) [137]. The proposed mechanism involves Lewis acid coordination to give complex **356**, which can be selectively excited in the presence of the unbound substrate. 356* then rearranges enantioselectively to generate cationic intermediate **356^+^**, which then undergoes further rearrangement to furnish bicyclic products **357** in good yields and excellent enantioselectivities (15 examples, up to 99:1 er).

**Scheme 60:**
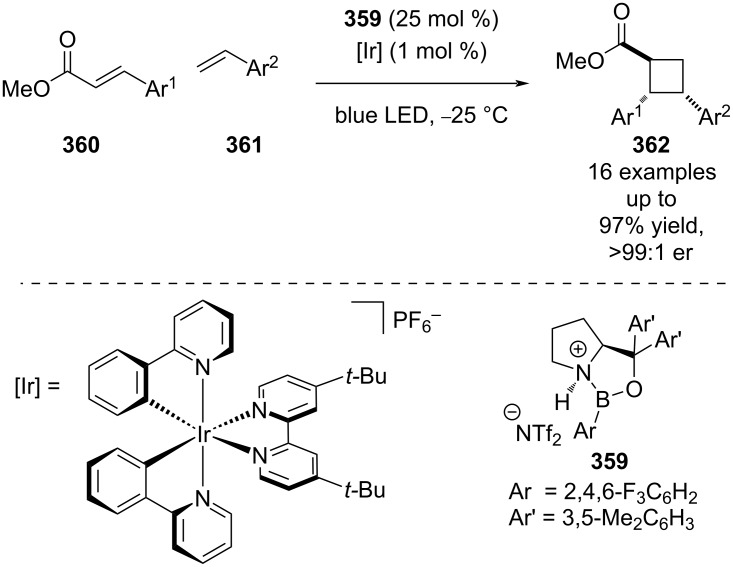
Lewis acid/photocatalysed [2 + 2] cycloadditions of cinnamate esters and styrenes.

Previous work on photocycloadditions by Yoon et al. relied on adjacent phenols to form Lewis acid complexes, which is a significant synthetic limitation. To generalise the reaction and expand the substrate scope, Yoon et al. found that a combination of an oxazaborilidine Lewis acid **359** with an external photocatalyst allowed for the cycloaddition of simple cinnamate esters **360** with styrenes **361** (Scheme 60) [138]. Instead of exploiting the red-shift in absorption wavelength to confer photochemoselectivity, Yoon et al. proposed that complexation of the Lewis acid lowers the triplet energy of the coordination complex to such an extent that selective triplet sensitization via Dexter energy transfer from the photocatalyst and subsequent enantioselective [2 + 2] cycloaddition becomes operative as was proposed with the scandium catalysts in Scheme 47 [113–114].

This section has clearly demonstrated the immense diversity in types of Lewis acids that are compatible with photocatalysis, varying from heavy transition metal catalysts and lanthanoid catalysts to boron-centred ones. With this diversity in LA catalyst, comes a broad scope of reactivity that has been made possible by this class of dual mode catalysis.

#### Transition metal catalysis

The previous section included some examples of transition metals acting as Lewis acids, whereas this section focuses more on reactivity that is unique to transition metals, including inner sphere electron transfer events within the putative mechanisms. Transition metal complexes with achiral ligands have been widely used in combination with photocatalysts in racemic dual catalytic reactions [139–140], with nickel/photoredox catalysis becoming a commonly used combination [141–142]. Transition metal complexes with chiral ligands have also been used extensively in enantioselective catalysis. A recent review on enantioselective metallaphotoredox catalysis summarises the combination of these two bodies of work well [15]. This section is further categorised into subsections by the transition metal used.

**Scheme 61:**
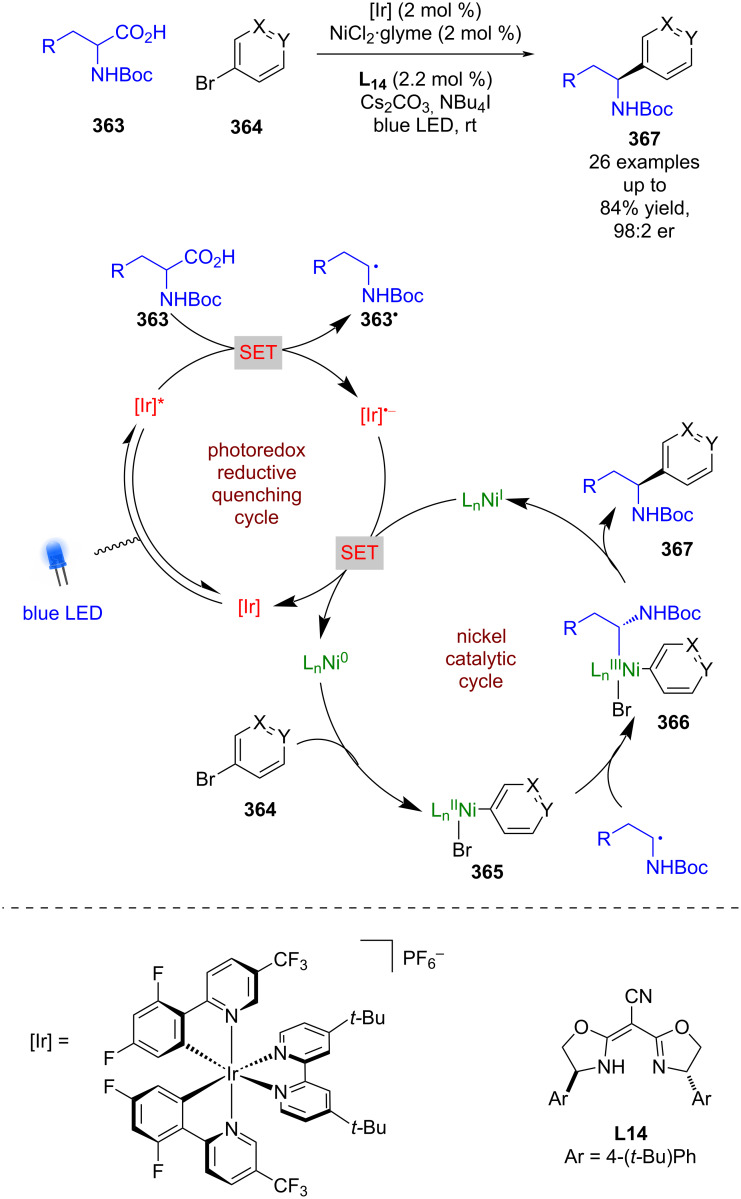
Nickel/photoredox-catalysed arylation of α-amino acids using aryl bromides.

**Nickel catalysis:** The first enantioselective example of this dual mode catalysis reported was a decarboxylative arylation of α-amino acids **363** with aryl bromides **364** bearing electron-withdrawing groups, developed by Fu and MacMillan using NiCl_2_, chiral ligand **L14**, and a heteroleptic iridium-based photocatalyst (Scheme 61) [143]. The proposed mechanism involves a reductive quenching cycle resulting in α-amino radicals **363^•^**, and a nickel catalytic cycle that starts with oxidative addition of **364** onto a Ni^0^ complex to give Ni^II^ intermediate **365**. **365** is then intercepted by **363^•^** to give a Ni^III^ intermediate **366**, which upon reductive elimination releases enantioenriched arylated products **367** in good yields and excellent enantioselectivities (26 examples, up to 98:2 er). Both catalytic cycles are completed by a SET step between Ni^I^ and [Ir]^•−^.

**Scheme 62:**
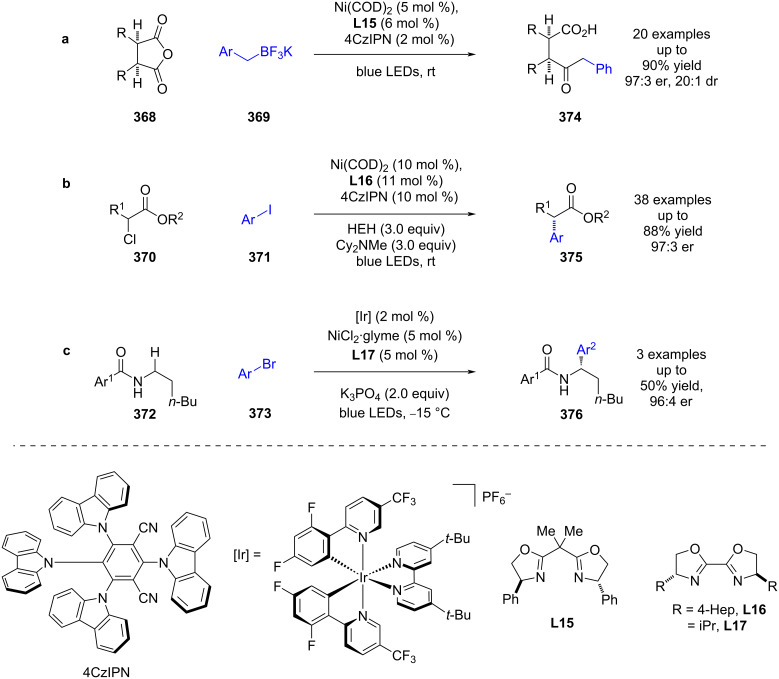
Nickel/photoredox catalysis. (a) Desymmetrisation of cyclic *meso*-anhydrides using benzyl trifluoroborate salts. (b) Cross-electrophile coupling of α-chloro esters with aryl iodides, using Hantzsch ester as a sacrificial reductant. (c) C–H Arylation of benzamides with aryl bromides.

Following this report, a large array of reactions has been developed using chiral ligands with nickel/photoredox catalysis [144–146]. For example, Rovis and Doyle developed a desymmetrisation of cyclic *meso*-anhydrides **368** using benzyl trifluoroborate salts **369**, chiral ligand **L15**, Ni(COD)_2_, and the photocatalyst 4CzIPN (Scheme 62a) [147]. More recently, Walsh and Mao used a similar dual catalyst system for the enantioselective cross-electrophile coupling of α-chloro esters **370** and aryl iodides **371** using Hantzsch ester as a sacrificial reductant (Scheme 62b) [148]. Montgomery and Martin achieved similar reactivity via a C–H arylation of benzamides **372** using aryl bromides **373**, although only a limited number of enantioselective examples were reported (Scheme 62c) [149].

**Scheme 63:**
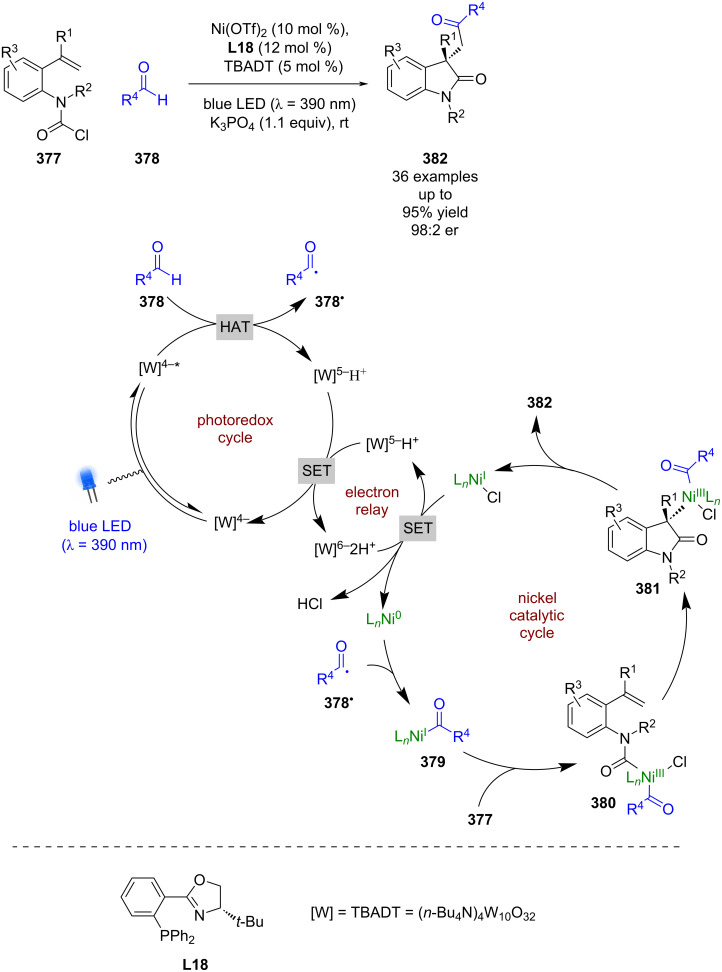
Nickel/photoredox catalysis for the acyl-carbamoylation of alkenes with aldehydes using TBADT as a HAT catalyst.

Expansion to a system using TBADT by Wang, which is known to generate acyl radicals from aldehydes [150], allowed for the enantioselective acyl-carbamoylation of alkenes **377** with aldehydes **378** (Scheme 63) [151]. The putative mechanism in this case, based on prior work by MacMillan et al. [152], proceeds via HAT from **378** to the excited state photocatalyst to generate acyl radical **378^•^** and [W]^5−^H^+^. Subsequent addition of **378^•^** to Ni^0^ affords Ni^I^ intermediate **379**, which oxidatively adds into **377** to give the Ni^III^ species **380**. **380** then undergoes an enantioselective migratory insertion to generate complex **381**, which can reductively eliminate to furnish the desired products **382** in excellent yields and enantioselectivities (36 examples, up to 98:2 er). Concomitantly [W]^5−^H^+^ undergoes a disproportionation reaction to generate [W]^4−^ and [W]^6−^2H^+^, which reduces the Ni^I^ species and completes the cycle. This nickel catalytic cycle [Ni(0), Ni(I), Ni(III)] is different to that shown in Scheme 61 [Ni(0), Ni(I), Ni(II), Ni(III)], but both are plausible and it is difficult to determine which is in operation. Towards this, Molander and Gutierrez recently reported an interesting computational investigation that assessed the feasibility of different possible mechanisms for tertiary radicals in nickel/photoredox dual catalysis and concluded that multiple mechanisms are plausible, and which one is in operation is both substrate and ligand dependant [153].

**Scheme 64:**
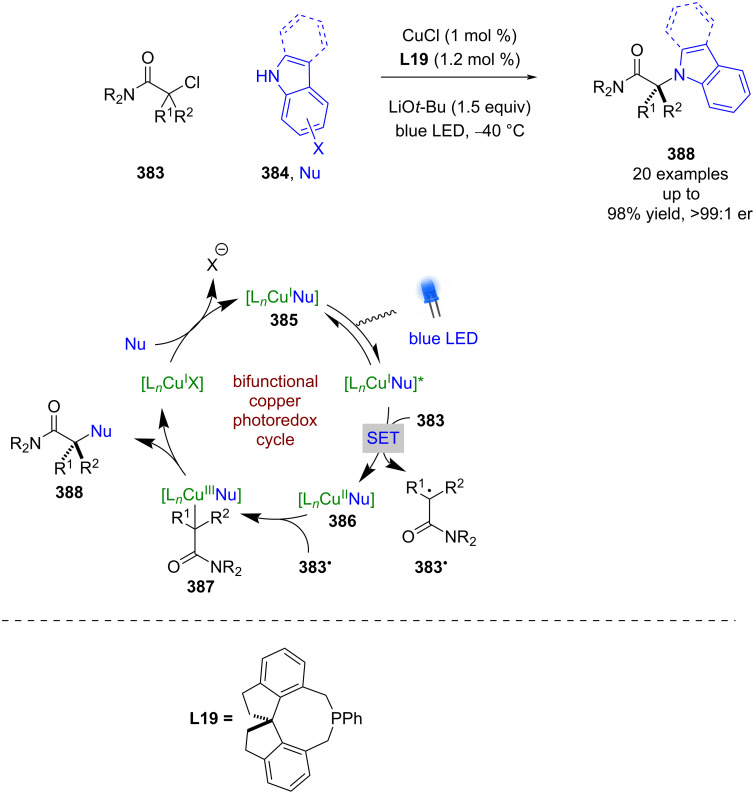
Bifunctional copper/photoredox-catalysed C–N coupling between α-chloro amides and carbazoles or indoles.

**Copper catalysis:** Other metal complexes have also been used in combination with photocatalysis. For example, Peters and Fu found that copper catalysts can mediate the C–N cross coupling of α-chloro amides **383** (Scheme 64) [154]. They propose that following ligand substitution with an indole or carbazole **384**, the resulting photoactive complex **385** is excited upon light absorption. The reaction then proceeds via an oxidative quenching pathway by **383** resulting in the Cu^II^ complex **386** and an alkyl radical **383^•^**, which then adds to copper to give a Cu^III^ intermediate **387** that reductively eliminates to complete the cycle and release enantioenriched C–N coupling product **388** in excellent yields and enantioselectivities (20 examples, up to >99:1 er).

**Scheme 65:**
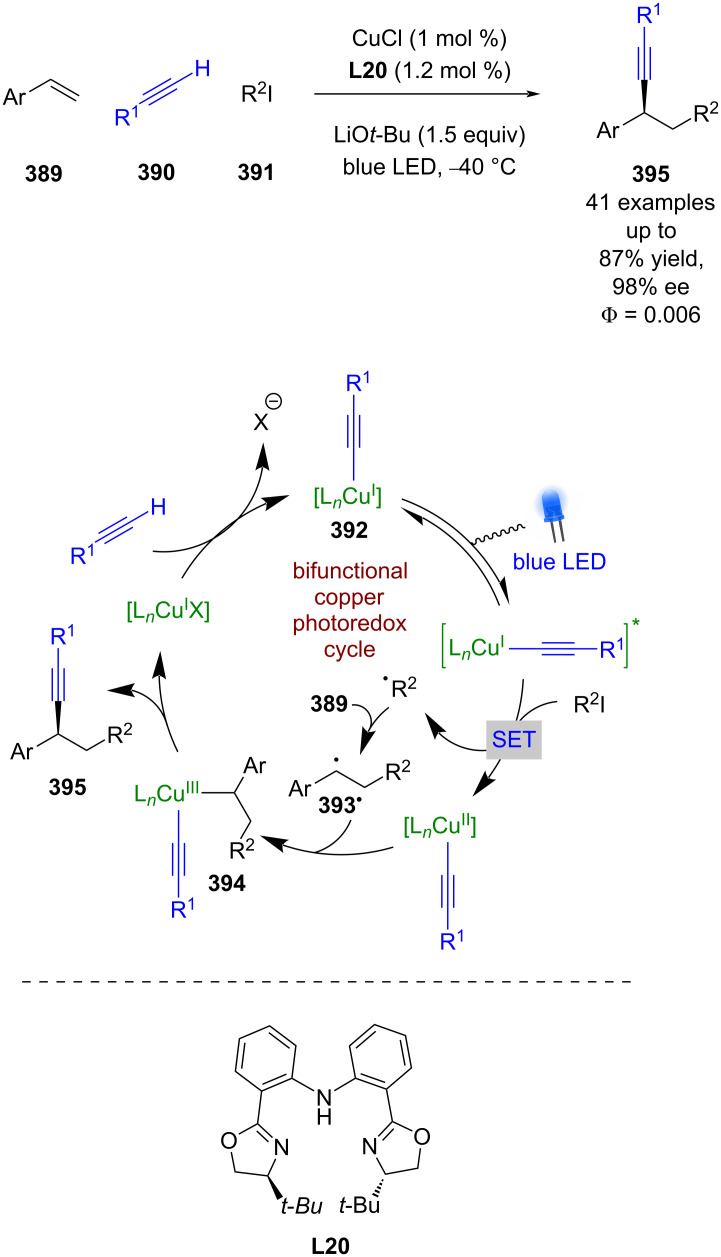
Bifunctional copper/photoredox-catalysed difunctionalisation of alkenes with alkynes and alkyl or aryl iodides.

Since this report, chiral copper complexes have been used in other enantioselective bifunctional photocatalysis reactions. Zhang et al., developed an enantioselective difunctionalisation of alkenes **389** using alkynes **390** and alkyl or aryl iodides **391** that proceeds via a radical cascade reaction (Scheme 65) [155]. They propose that the copper acetylide intermediate **392** is photoactive and photocatalyses the reaction, proceeding via an oxidative quenching pathway. The formed radicals then add to **389** to afford alkyl radical **393^•^**, which then adds to the Cu^II^ centre to give a Cu^III^ intermediate **394**, which upon enantioselective reductive elimination completes the cycle and generates the desired products **395** in good yields and excellent enantioselectivities (41 examples, up to 99:1 er). The quantum yield was measured to be <1 (Φ = 0.006), suggesting a radical chain mechanism is unlikely to be dominant.

**Scheme 66:**
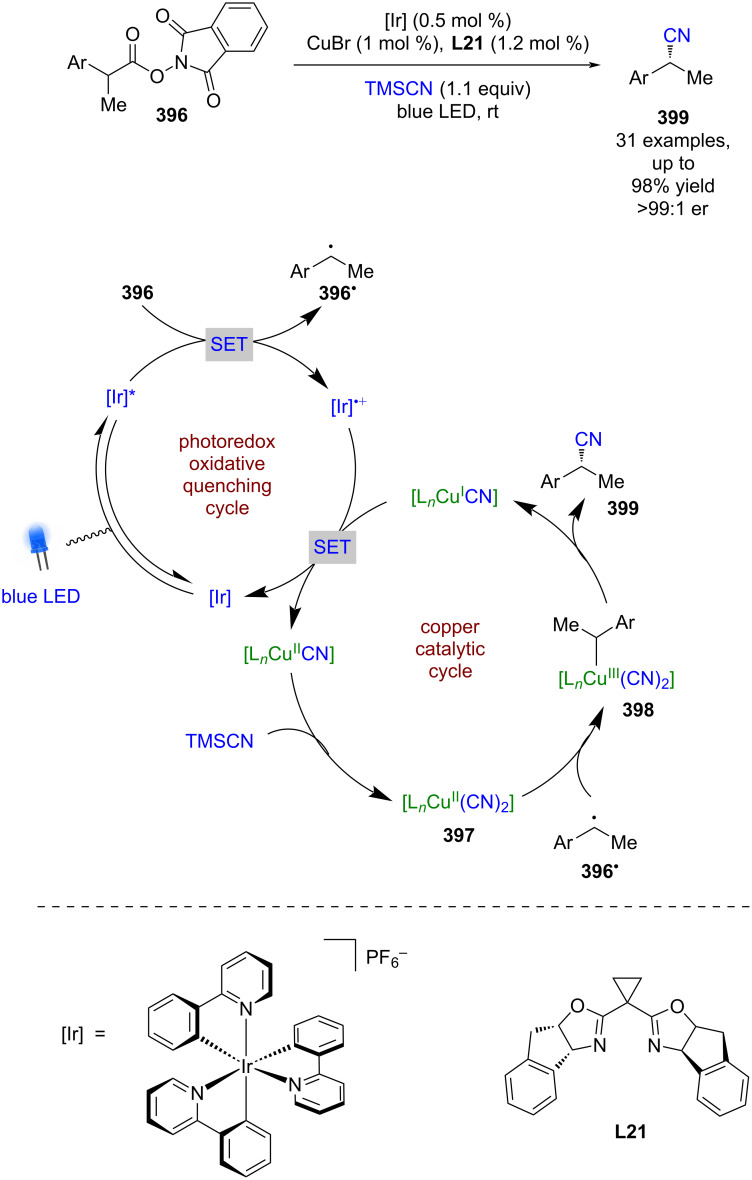
Copper/photoredox-catalysed decarboxylative cyanation of benzyl phthalimide esters.

Enantioselective copper catalysis has also been used in combination with an external photocatalyst. For instance, Liu et al. reported a decarboxylative cyanation of phthalimide esters **396** using a combination of CuBr, TMSCN, an iridium-based photocatalyst, and chiral ligand **L21** (Scheme 66) [156]. Based on previous cyanation reactions [157], they propose an oxidative quenching cycle where benzyl radicals **396^•^** are formed from **396** after a reductive decarboxylation, [Ir]^•+^ then oxidises the Cu^I^ catalyst to Cu^II^ which accepts a second cyanide ion to generate the active dicyano species **397**. Radical addition of **396^•^** to **397** generates Cu^III^ intermediate **398**, which after reductive elimination regenerates the catalyst and produces enantioenriched nitriles **399** in excellent yields and enantioselectivities (31 examples, up to >99:1 er).

**Scheme 67:**
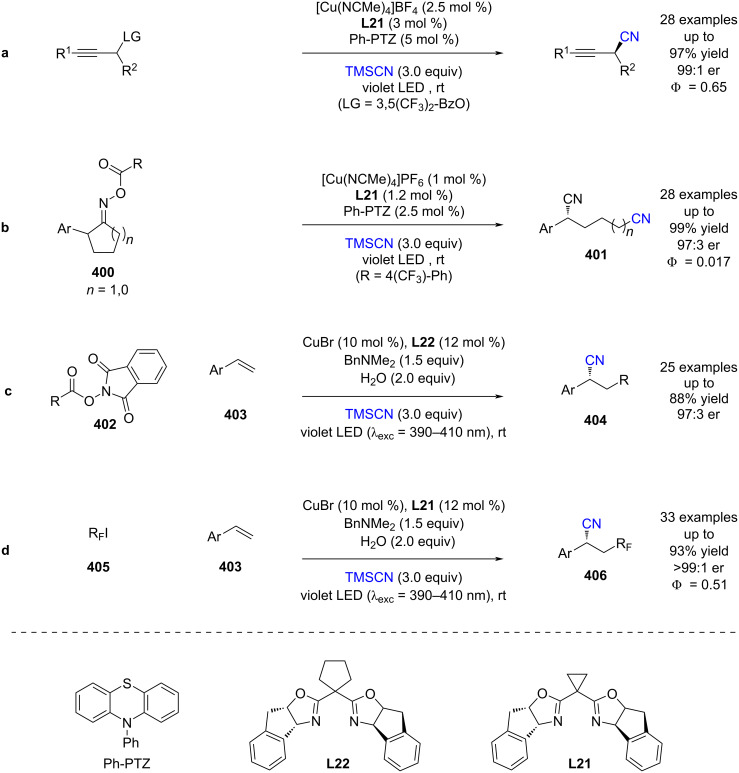
Copper/photoredox-catalysed cyanation reactions using TMSCN. (a) Propargylic cyanation (b) Ring opening cyanation of oxime esters. (c) Radical relay reaction using phthalimide esters and styrenes. (d) Bifunctional catalyst for the difunctionalisation of alkenes using perfluoroalkyl iodides and styrenes.

A series of cyanation reactions have subsequently been developed using similar reactivity. For example, propargyl radicals were successfully used in concert with the more strongly reducing Ph-PTZ photocatalyst, and with a different leaving group to achieve the first enantioselective propargylic radical cyanation (Scheme 67a) [158]. Employing oxime esters **400** as radical precursors afforded enantioenriched dicyano alkanes **401** (Scheme 67b) [159]. Han and Mei developed a radical cascade system using phthalimide esters **402**, styrenes **403**, and TMSCN to obtain enantioenriched difunctionalised products **404** (Scheme 67c) [160]. Wang and Xu reported a similar radical cascade process using a single catalyst system with perfluorinated alkyl iodides **405** and TMSCN to afford enantioenriched difunctionalised alkenes **406** via a similar mechanism to that proposed by Fu [154] in Scheme 64 (Scheme 67d) [161]. Notably, the quantum yields that were measured for these reactions were all <1, suggesting chain mechanisms may not be dominant but also showing reactions Scheme 67a (Φ = 0.65) and Scheme 67d (Φ = 0.51) are quite efficient processes.

**Scheme 68:**
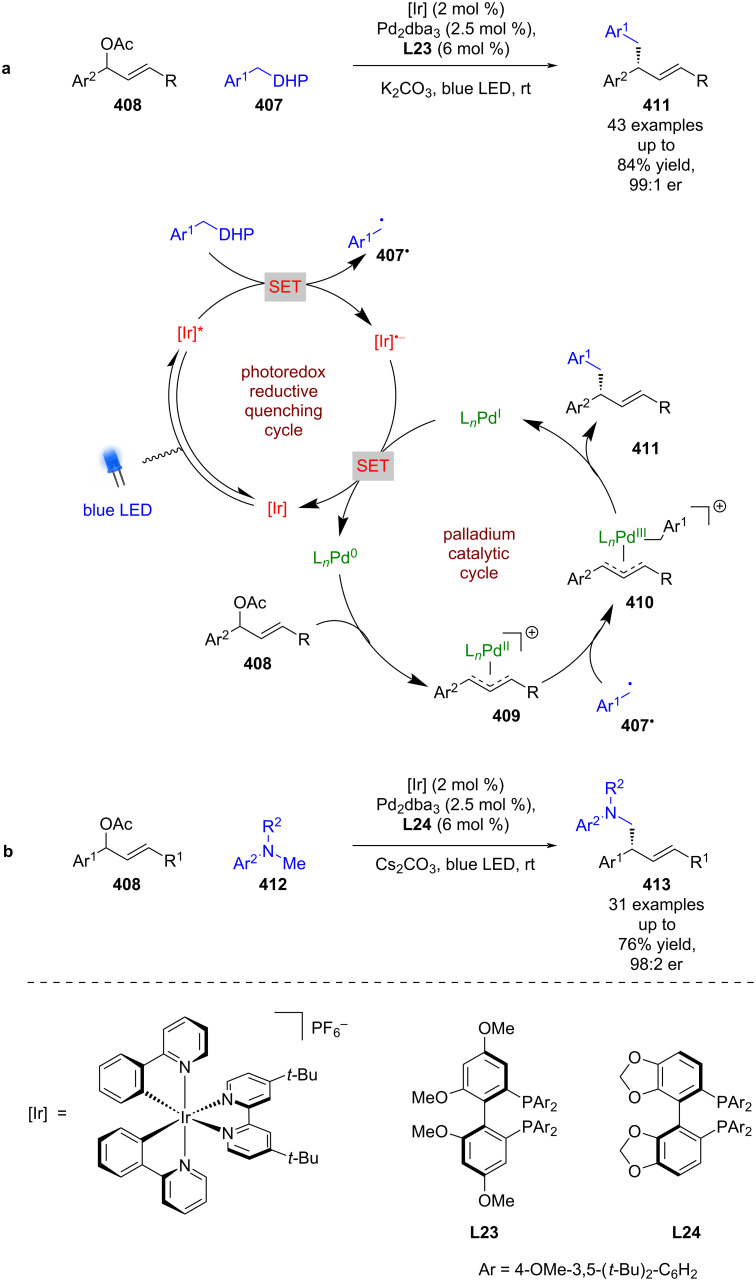
Palladium/photoredox-catalysed allylic alkylation reactions. (a) Using alkyl DHPs as radical precursors. (b) Using tertiary amines as radical precursors.

**Palladium catalysis:** While there have now been many examples of dual palladium catalysis merged with photocatalysis [140], there are relatively few enantioselective variations. A highly enantioselective reaction using palladium and photoredox catalysis was reported by Yu et al. using chiral ligand **L23** in an allylic alkylation reaction (Scheme 68a) [162]. Using DHPs **407** as radical precursors, they propose an analogous set of catalytic cycles to those proposed for nickel catalysis. The iridium-based photocatalyst proceeds via a reductive quenching cycle to form benzylic radicals **407^•^**; simultaneously Pd^0^ reacts with alkene **408** to form a π-allyl palladium(II) complex **409**, which is then intercepted by **407^•^** to form a Pd^III^ intermediate **410**. Subsequent reductive elimination releases enantioenriched allylation products **411** in good yields and excellent enantioselectivities (43 examples, up to 99:1 er). A SET event between the resulting Pd^I^ and [Ir]^•−^ then completes both catalytic cycles. Yu et al. recently extended this reactivity to anilines **412** that can form α-amino radicals under similar reaction conditions to obtain the corresponding products **413** in comparable yields and enantioselectivities (31 examples, up to 98:2 er) (Scheme 68b) [163].

**Scheme 69:**
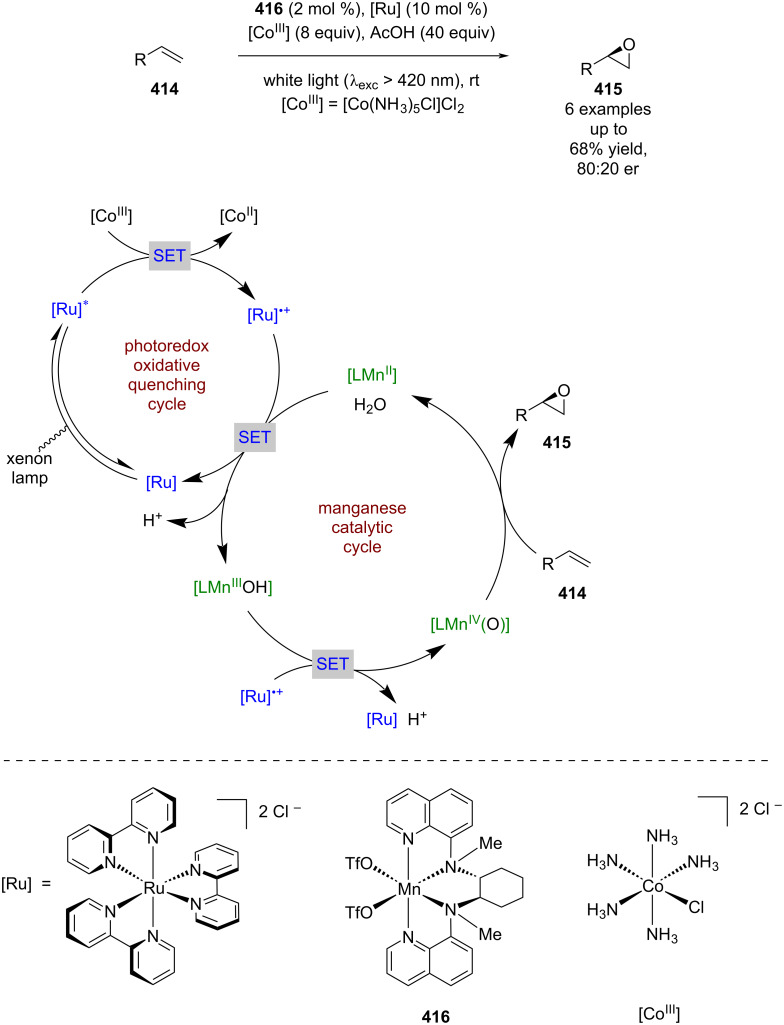
Manganese/photoredox-catalysed epoxidation of terminal alkenes.

**Manganese catalysis:** Dual catalysis involving a manganese catalyst in combination with photoredox catalysis is far less explored. An example was reported by Nam et al. for the enantioselective epoxidation of terminal alkenes **414** using H_2_O as the oxygen source (Scheme 69) [164]. The proposed mechanism uses a stoichiometric cobalt reagent as a sacrificial oxidant in an oxidative quenching cycle to generate [Ru]^•+^, which then oxidises Mn^II^ in the presence of water to Mn^III^OH and turn over the photocatalytic cycle. Another similar SET event generates the active Mn^IV^O species, which catalyses the epoxidation of **414** to give epoxides **415** in moderate yields and enantioselectivities (6 examples, up to 80:20 er). The two SET events required imply a two-photon mechanism is in operation; however, this is not discussed further by the authors. Interestingly, acetic acid seems vital for both yield and enantioselectivity, although the reason for this remains unclear.

**Scheme 70:**
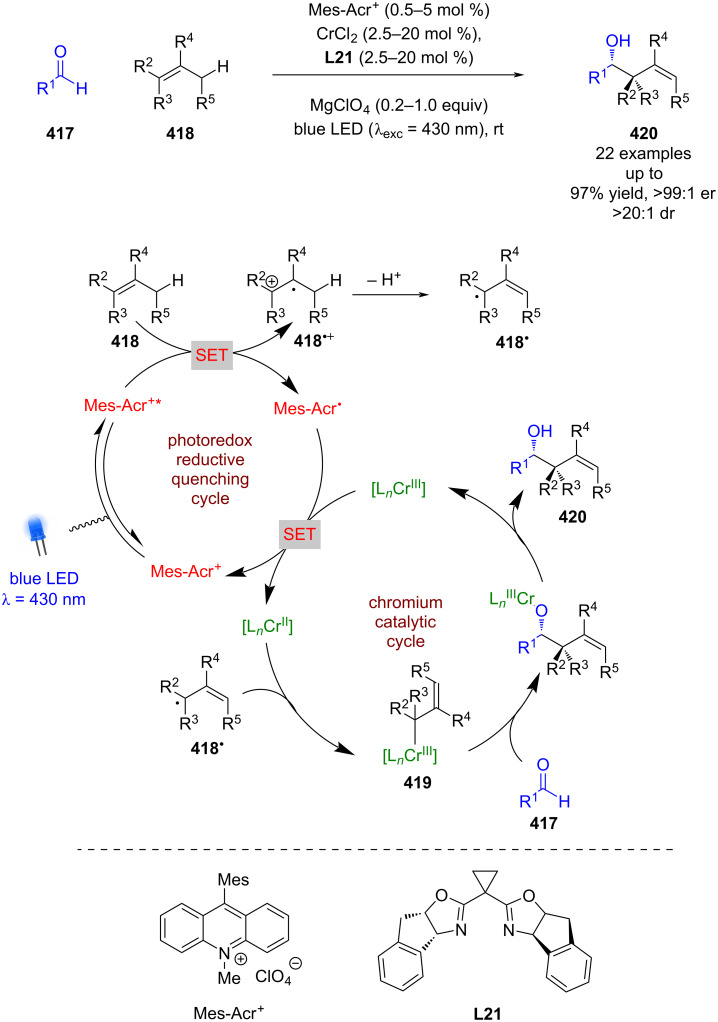
Chromium/photoredox-catalysed allylation of aldehydes.

**Chromium catalysis:** Kanai et al. combined chromium catalysis with Mes-Acr^+^ for the enantioselective allylation of aldehydes **417** in the presence of chiral ligand **L21** (Scheme 70) [165]. They proposed that the reaction proceeds via a reductive quenching cycle with unactivated alkenes **418** to form radical cations **418^•+^** that can be deprotonated to give allylic radicals **418^•^**. Radicals **418^•^** can then be intercepted by a Cr^II^ catalyst to form a Cr^III^ intermediate **419**, which can then nucleophilically add to the aldehyde to give enantioenriched alcohols **420** after protonation in excellent yields and enantioselectivities (22 examples, up to >99:1 er). The resulting Cr^III^ species can then undergo a SET event with PC^•−^ to complete both catalytic cycles.

#### Enzyme catalysis

**Scheme 71:**
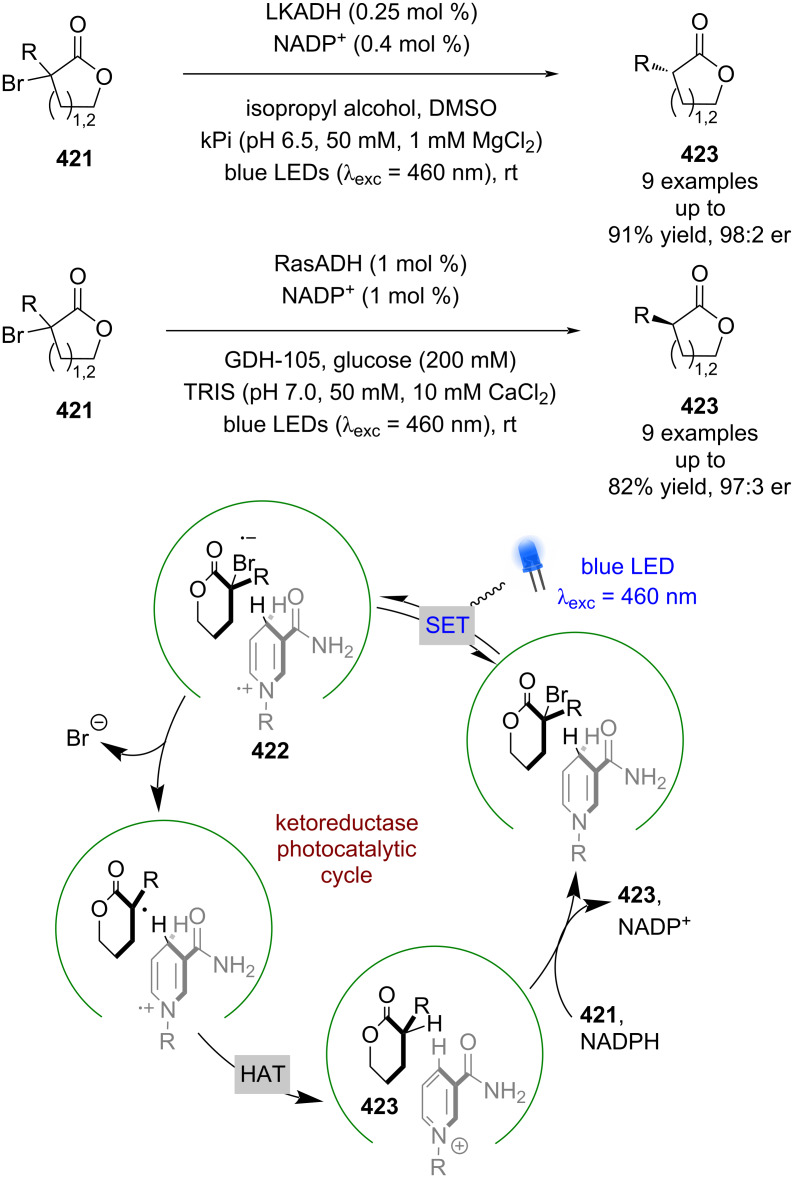
Enzyme/photoredox-catalysed dehalogenation of halolactones.

Enzymes in nature use their bespoke binding environments to catalyse reactions with extreme selectivity; however, there are only a limited number of reactions that occur in nature where this is possible [166]. One method to extend their reactivity is to use them in combination with photocatalysis. There are now many examples that employ this strategy for both racemic and enantioselective transformations and these are well covered in Gulder’s review [7]. One of the earliest enantioselective examples was reported by Hyster et al. for the dehalogenation of halolactones **421** in the presence of ketoreductases (KREDs) and NADPH (Scheme 71) [167]. The proposed mechanism implicates an enzyme active site containing **421** and NADPH that forms an EDA complex, which upon photoexcitation gives intermediate **422**. Loss of bromide followed by enantioselective HAT from NADPH^•+^ gives enzyme-bound lactone **423** and NADP^+^. Both compounds are then displaced by **421** and NADPH to complete the cycle, with NADP^+^ being reduced either by isopropyl alcohol or glucose dehydrogenase (GDH). Different conditions can also be used to synthesise either enantiomer in excellent yields and enantioselectivities (9 examples, up to 98:2 and 97:3 er), which is important for enzymatic reactions as one cannot simply use the opposite enantiomer.

**Scheme 72:**
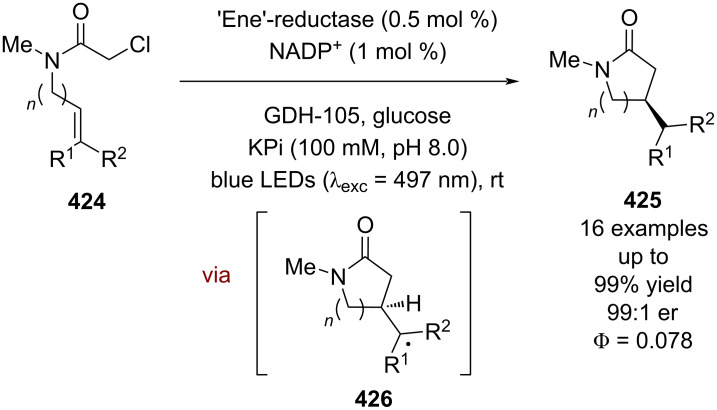
Enzyme/photoredox-catalysed dehalogenative cyclisation.

Hyster et al. expanded this methodology to the cyclisation reactions of α-chloro amides **424** (Scheme 72) [168]. In this instance, ‘ene’-reductases (ERs) were found to be optimal and HAT occurs after radical addition to the pendant alkene to give enantioenriched lactams **425** via tertiary radical **426** in excellent yields and enantioselectivities (16 examples, up to 99:1 er). The quantum yield of the reaction was determined to be <1 (Φ = 0.078), so a radical chain reaction is unlikely.

**Scheme 73:**
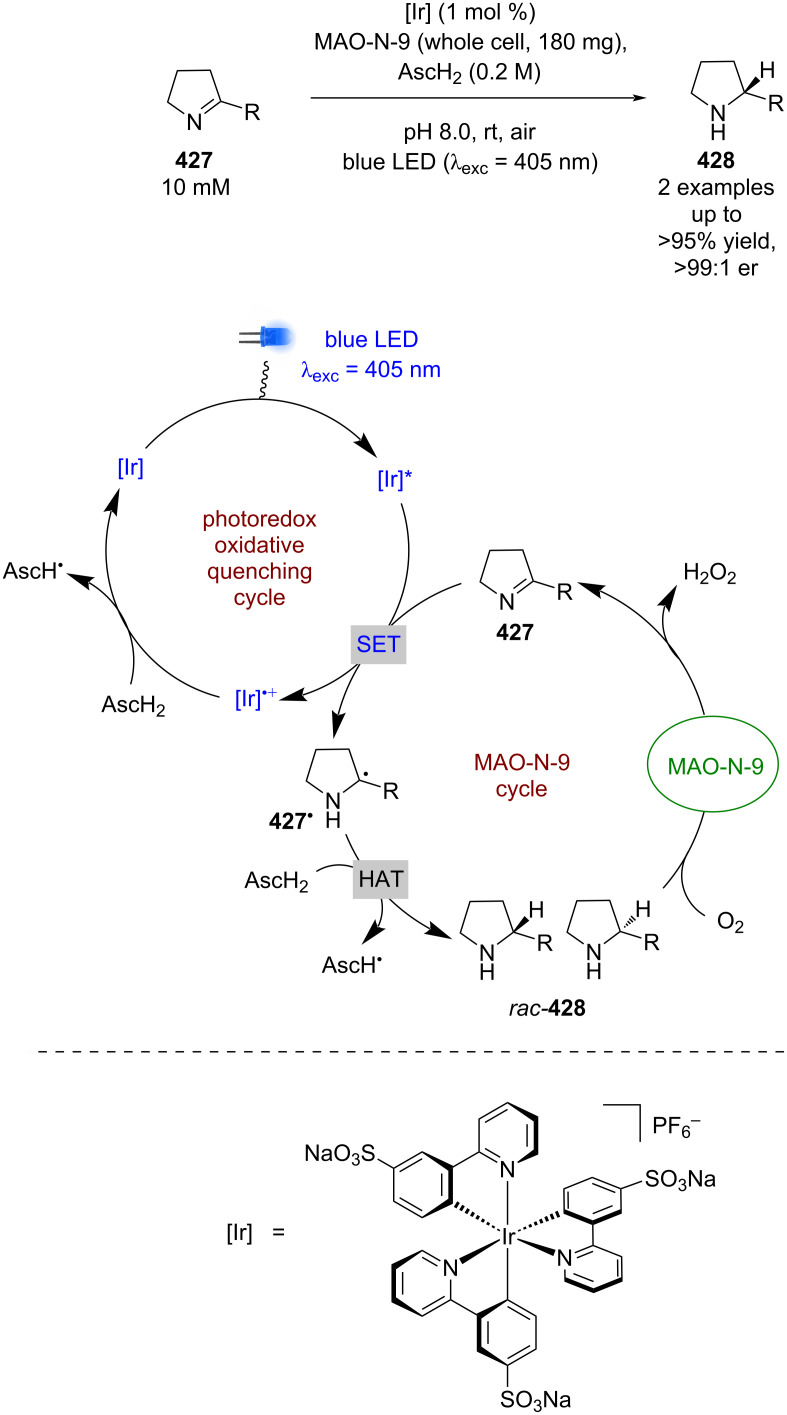
Enzyme/photoredox-catalysed reduction of cyclic imines.

An example of enzymes being used in combination with an external photocatalyst was developed by Ward and Wenger using a water-soluble iridium photocatalyst for the enantioselective reduction of cyclic imines **427** (Scheme 73) [169]. The reaction is proposed to proceed via an oxidative quenching cycle to give alkyl radical **427^•^**, which is then trapped by ascorbic acid (AscH_2_) in a HAT process to give a racemic mixture of cyclic amine **428**. When coupled with a highly selective enzyme-mediated oxidation using a monoamine oxidase (MAO-N-9), the (*S*)-enantiomer can be selectively removed and recycled to give the near enantiopure (*R*)-amine. The scope of this transformation was found to be limited to two substrates, allowing for little variation in structure or substituents.

**Scheme 74:**
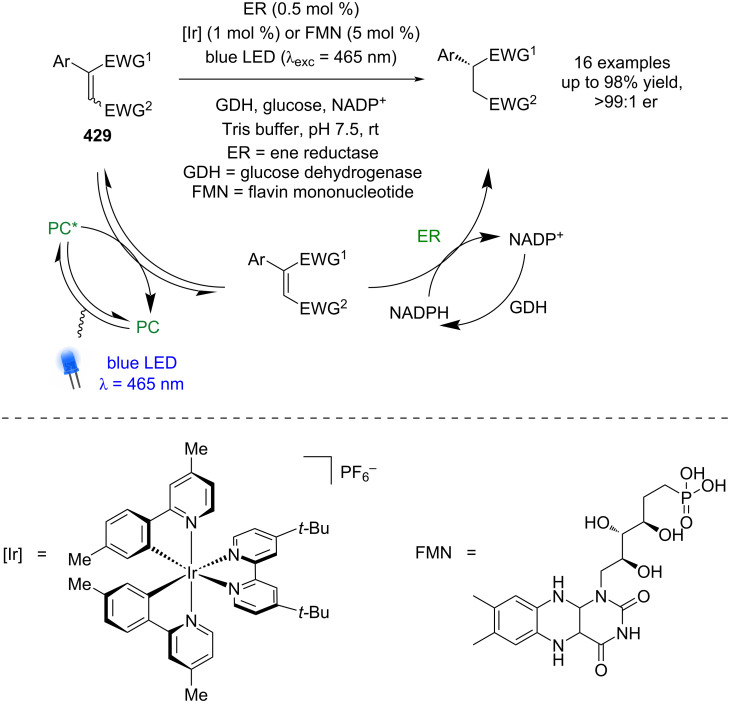
Enzyme/photocatalysed enantioselective reduction of electron-deficient alkenes as mixtures of (*E*)/(*Z*)-isomers.

Zhao and Hartwig reported an enantioselective reduction of electron-deficient alkenes (Scheme 74) [170]. To do this, the authors assembled a collection of electron-deficient alkenes **429** that interacted with ERs for the selective reduction of either the (*E*) or (*Z*)-isomers. Separately, they optimised photoisomerisation conditions using either a flavin (FMN) or iridium-based photocatalyst, which proceeds through an energy transfer process. These reactions were combined for the enantioselective reduction of either the mismatched isomer of the alkene or mixtures of isomers where separation is not possible, in excellent yields and enantioselectivities (16 examples, up to >99:1 er).

**Scheme 75:**
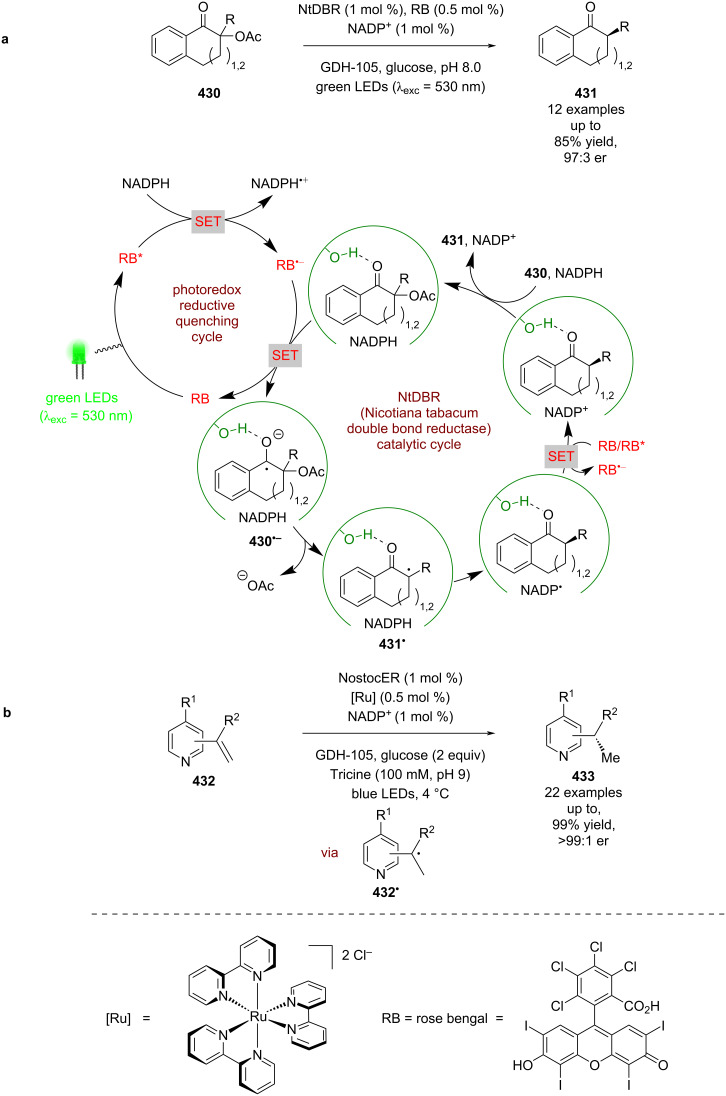
Enzyme/photoredox catalysis. (a) Deacetoxylation of cyclic ketones. (b) Reduction of heteroaromatic alkenes.

A significant challenge in many enantioselective photocatalysed reactions is minimising the amount of racemic background reaction. Hyster et al. proposed that the binding of a substrate by an enzyme could alter its redox properties so that the photoredox step only occurs within the enzyme binding site. They applied this hypothesis to a deacetoxylation reaction of tetralones **430** (Scheme 75a) [171]. The authors propose that Rose Bengal (RB) proceeds through a reductive quenching cycle in the presence of NADPH to give RB^•−^, which then reduces enzyme-bound **430** preferentially over free **430** to give radical anion **430^•−^**. Deacetoxylation generates tertiary radical **431^•^** and subsequent enantioselective HAT with NADPH releases enantioenriched tetralone **431** after oxidation of NADP^•^ in good yields and enantioselectivities (12 examples, up to 97:3 er). This methodology was recently expanded to include heteroaromatic alkenes **432** using a similar single electron reduction to alkyl radical **432^•^** followed by ER-mediated enantioselective HAT to give reduced products **433** in excellent yields and enantioselectivities (22 examples, up to >99:1 er) (Scheme 75b) [172].

**Scheme 76:**
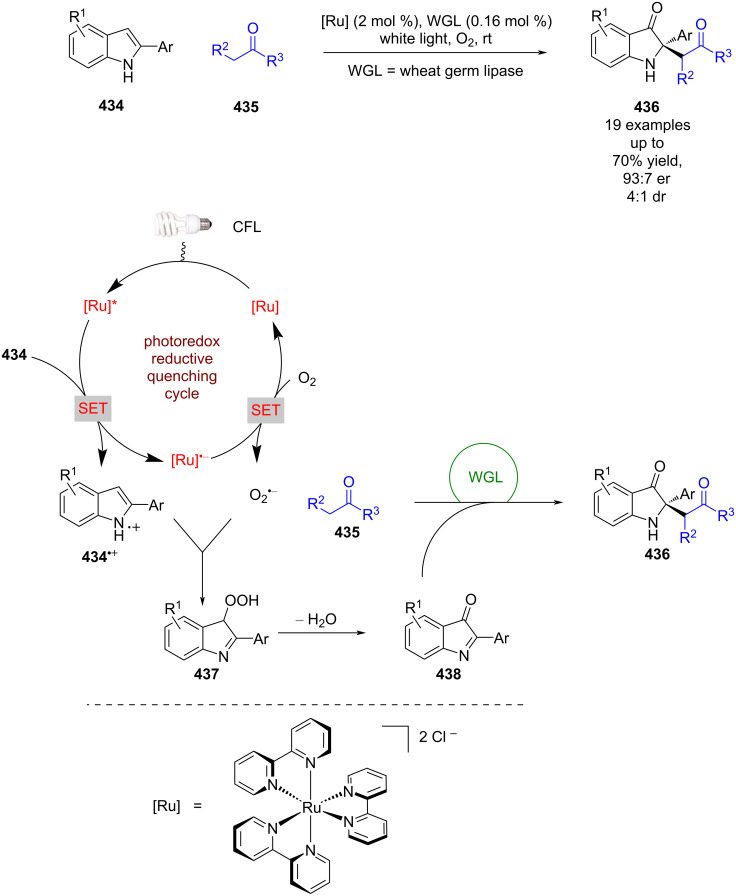
Enzyme/photoredox-catalysed synthesis of indole-3-ones from 2-arylindoles.

Guan and He developed a concurrent photooxidation and enzyme-mediated alkylation of indoles **434** with ketones **435** to obtain enantioenriched indole-3-ones **436** using wheat germ lipase (WGL) (Scheme 76) [173]. This reaction is proposed to proceed via a reductive quenching cycle, producing radical cation **434^•+^** and [Ru]^•−^, which is then oxidised by O_2_ to complete the cycle and release O_2_^•−^. Radical cation **434^•+^** can then trap O_2_^•−^ to form hydroperoxyl intermediate **437**, that upon loss of water, is further oxidised to the indole-3-one **438**. Within the enzyme active site, **435** can nucleophilically add to **438** to give enantioenriched **436** in good yields and enantioselectivities (19 examples, up to 93:7 er).

**Scheme 77:**
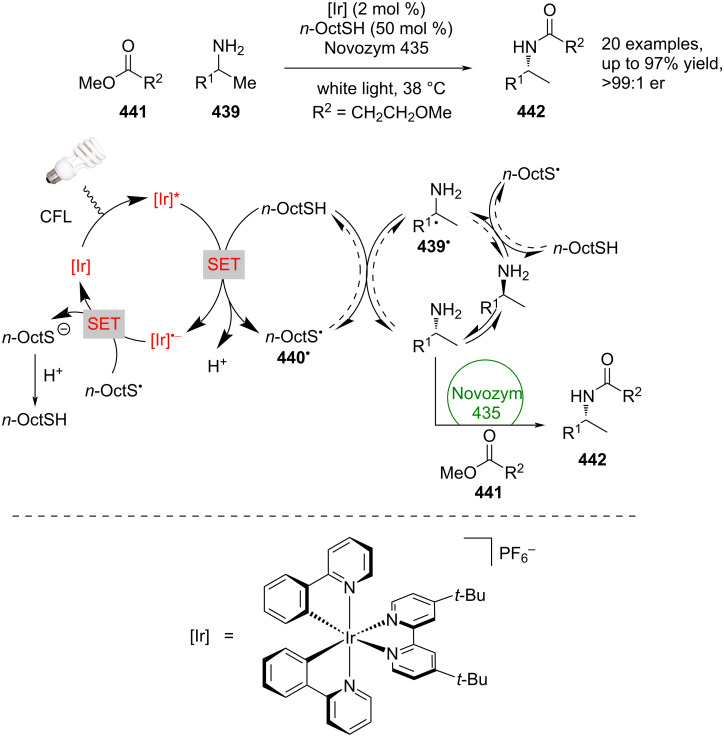
Enzyme/HAT/photoredox catalysis for the DKR of primary amines.

Enzymes are commonly used for kinetic resolutions of primary amines [174]. However, by nature they are limited to a theoretical 50% yield; dynamic kinetic resolutions (DKRs) are a common adaptation that can increase the theoretical yield to 100% but require a mechanism for racemisation of the disfavoured enantiomer. Zhou et al. developed a dual catalytic system for the racemisation of amines **439** using HAT and photoredox catalysis [175]. They then combined this with an enzyme-mediated acylation to achieve a DKR of primary amines using Novozym 435 (Scheme 77). The proposed mechanism for this process involves a reductive quenching cycle with *n*-octylthiol to generate thiyl radicals **440^•^** and release of H^+^. The thiyl radical **440^•^** is then implicated in either the turnover of the photocatalyst to regenerate the thiol or abstraction of a hydrogen atom from **439** to give α-amino radical **439^•^**, which in turn can abstract a hydrogen atom from the thiol to give either enantiomer of **439**, setting up a series of equilibria. When used in the presence of the appropriate enzyme and acylating reagent **441**, a single enantiomer is preferentially acylated to give enantioenriched amides **442** in excellent yields and enantioselectivities (20 examples, up to >99:1 er).

**Scheme 78:**
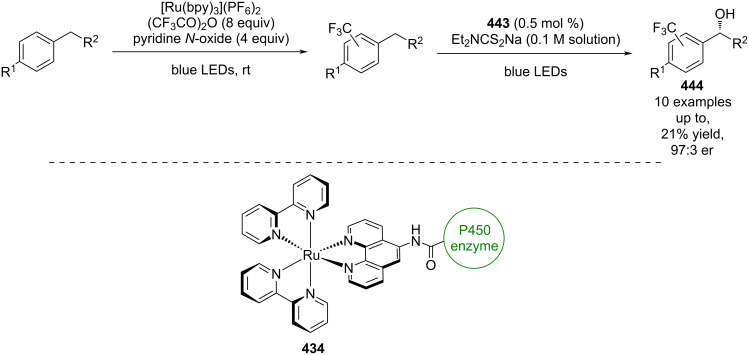
Bifunctional enzyme/photoredox-catalysed benzylic C–H hydroxylation of trifluoromethylated arenes.

Cheruzel et al. has synthesised a series of hybrid metalloenzymes to include ruthenium photosensitising units [176–177] and have recently applied them as bifunctional photocatalysts (Scheme 78) [178]. They combined a known trifluoromethylation reaction, with a hydroxylation catalysed by bifunctional photocatalyst **443** to synthesise alcohols **444**. Notably, the initial trifluoromethylation gives a mixture of isomers and the enzyme used can selectively oxidise different isomers, which explains the low yields.

## Conclusion

Over the last 15 years enantioselective photocatalysis has seen tremendous growth. Well-established modes of enantioselective catalysis have been revolutionised by the introduction of photochemical processes, be that altering the behaviour of enzyme systems or utilising familiar catalytic intermediates, such as enamines or metal complexes, with photoredox-generated radicals.

There are certain photochemical processes that are still underutilised in the context of enantioselective photocatalysis. EnT reactions in particular are investigated far less than photoredox catalysis, leading to a field largely dominated by [2 + 2] cycloadditions with significantly less reaction diversity, even though the works of Bach and Meggers show the potential for greater reaction scope. Comparatively, enantioselective photoredox catalysis is well-established; however, a somewhat limited series of functional groups have been documented as radical precursors in this review, such as tertiary amines, carboxylic acids, and phthalimide esters. Recent developments in photoredox catalysis have allowed for the generation of radicals from much more challenging substrates by increasing the reducing power of the photocatalyst. It is expected that this will carry through to asymmetric catalysis and allow for a wider scope of enantioselective transformations.

Analogously, combinations of photocatalysts with new modes of enantioselective catalysis will also allow for new reactivity, for example, expanding transition metal dual catalysis systems to other metals or further developing existing systems such as NHC dual catalysis. Additionally, while there has been a considerable amount of research carried out using carbonyl-derived substrates, less functionalised starting materials generally have not been investigated to the same degree within enantioselective photocatalysis. Illustrative of this point, of the 114 reactions shown in this review, 92 of them exploit a carbonyl or carbonyl derivative for enantioselectivity.

Most photocatalytic transformations rely on external photocatalysts; however, as exemplified by the works of Melchiorre, Alemán, and others, recognising that substrates or intermediates can be themselves photoactive allows for the simplification of reaction procedures and also for new reactivity. It would be interesting to see further developments in this field by exploiting other potentially photoactive reaction intermediates in the same way.

As previously mentioned, a deeper underpinning understanding of the mechanisms and fundamental photophysics of enantioselective photocatalysis will significantly accelerate the development of new reactions. Therefore, both experimental and computational mechanistic investigations of existing reactions are crucial for the future of this field.
